# In vitro construction of the COQ metabolon unveils the molecular determinants of coenzyme Q biosynthesis

**DOI:** 10.1038/s41929-023-01087-z

**Published:** 2024-01-03

**Authors:** Callum R. Nicoll, Laura Alvigini, Andrea Gottinger, Domiziana Cecchini, Barbara Mannucci, Federica Corana, María Laura Mascotti, Andrea Mattevi

**Affiliations:** 1 Department of Biology and Biotechnology ‘Lazzaro Spallanzani’, University of Pavia, Pavia, Italy; 2 ’Centro Grandi Strumenti’, University of Pavia, Pavia, Italy; 3 Molecular Enzymology Group, Groningen Biomolecular Sciences and Biotechnology Institute, University of Groningen, Groningen, the Netherlands; 4 IMIBIO-SL CONICET, Facultad de Química Bioquímica y Farmacia, Universidad Nacional de San Luis, San Luis, Argentina

## Abstract

Metabolons are protein assemblies that perform a series of reactions in a metabolic pathway. However, the general importance and aptitude of metabolons for enzyme catalysis remain poorly understood. In animals, biosynthesis of coenzyme Q is currently attributed to ten different proteins, with COQ3, COQ4, COQ5, COQ6, COQ7 and COQ9 forming the iconic COQ metabolon. Yet several reaction steps conducted by the metabolon remain enigmatic. To elucidate the prerequisites for animal coenzyme Q biosynthesis, we sought to construct the entire metabolon in vitro. Here we show that this approach, rooted in ancestral sequence reconstruction, reveals the enzymes responsible for the uncharacterized steps and captures the biosynthetic pathway in vitro. We demonstrate that COQ8, a kinase, increases and streamlines coenzyme Q production. Our findings provide crucial insight into how biocatalytic efficiency is regulated and enhanced by these biosynthetic engines in the context of the cell.

Coenzyme Q (CoQ) or, more commonly, ubiquinone is renowned for its function as an electron shuttle and conduit in the electron respiratory chain^1–3^. CoQ also governs a whole host of different reactions and is tied to many metabolic pathways including fatty acid oxidation, mitochondrial uridine biosynthesis and more recently, of mounting interest, ferroptosis^4–6^. Indeed, primary CoQ deficiency is associated to several pathologies including cerebellar ataxia, cardiomyopathy and nephropathy, to name a few^7,8^.

CoQ is considered one of the most hydrophobic molecules known in nature^9^. It is synthesized in the mitochondria at the interface between the inner mitochondrial membrane and the matrix and comprises two juxtaposing ends: a highly hydrophobic poly-isoprenoid tail that anchors the antioxidant within phospholipid bilayers and a fully substituted aromatic head group responsible for its redox properties (Fig. 1a)^10–12^. The human ubiquinone is named coenzyme Q_10_, after its 10-isoprene unit tail. The polyisoprene moiety is first constructed by the heterodimeric PDSS1−PDSS2 prenyldiphosphate synthase and then ligated to the C_3_ position of benzoic acid by COQ2 (ref. 13). The benzoic acid moiety is then extensively modified through an elaborate series of enzymatic steps (Fig. 1b). The enzymes responsible for these reactions are similar but not identical among the kingdoms of life. However, besides this diversity, a conserved feature is that they collectively operate as a protein assembly, forming an iconic metabolon, often referred to as Complex Q or the CoQ synthome^12^. As defined by Schere, metabolons are a collection of proteins that form supramolecular assemblies to facilitate a series of enzymatic steps in a metabolic pathway^14,15^. Regarding the eukaryotic COQ metabolons, a composite of COQ proteins bands together on the matrix side of the inner mitochondrial membrane to build the final aromatic ring of CoQ. These include COQ3, COQ4, COQ5, COQ6, COQ7 and COQ9 (refs. 12,16–19).

The formation and regulation of the COQ metabolon is postulated to be facilitated by COQ8, which in humans is present as two paralogues, COQ8A and COQ8B (ref. 19).

The functional roles behind several of the animal COQ proteins remain bereft in our biochemical understanding. Indeed, the molecular determinants essential to C_1_ modification of the CoQ precursor and physiological roles of COQ8 in CoQ biosynthesis are unclear. Further questions include the following: Is the formation of the COQ metabolon, or subcomplexes, essential for enzymatic activity and does further protein complexation bolster enzymatic activity? Additionally, how is CoQ biosynthesis regulated? Major challenges associated to these questions are due to the extreme hydrophobicity and instability of the intermediates of the biosynthetic pathway, as well as the poor stability of the COQ partners as stand-alone systems. As such, building and investigating metabolons, in general, in vitro is arduous.

In this Article, to address these questions and uncover the roles of the COQ proteins at the helm of animal CoQ biosynthesis within the COQ metabolon, we employ an innovative evolution-engineering approach to build the COQ metabolon in vitro. We integrate ancestral sequence reconstruction (ASR) as a methodological backbone to our biochemical study as a means to build experimentally tractable systems with heightened stability^20,21^. To overcome issues with substrate solubility, we turn to CoQ precursors possessing a single isoprenoid unit. Our approach, based on the reconstruction of the full-scale biosynthetic pathway, unveils all the enzymatic steps in CoQ biosynthesis and demonstrates the functional coupling among the enzymes forming the metabolon.

## Results

### ASR provides experimentally tractable COQ proteins

**Extended Data Fig. 1 |:**
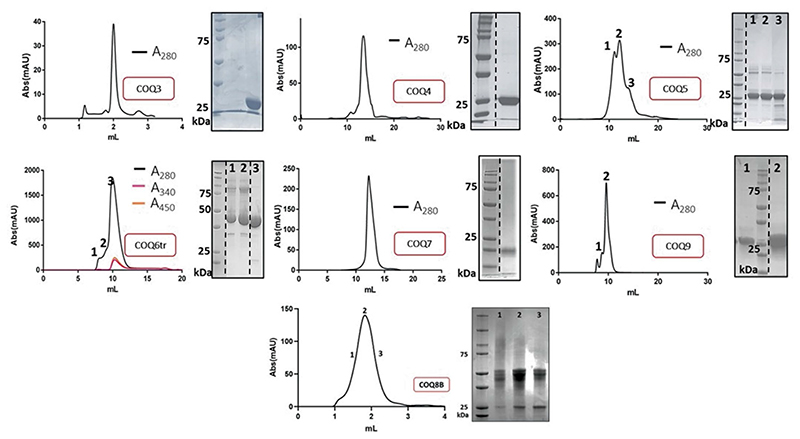
Ancestral COQ proteins show good purity and no aggregation in gel-filtration. Analytical (COQ3, COQ8B) or preparative (COQ4, COQ5, COQ6, COQ7, COQ9) Size Exclusion Chromatography profiles and SDS-PAGE analysis of purified COQ proteins (Supplementary Fig. 16). Size Exclusion Chromatography and SDS-PAGE analyses were repeated in n = 3 independent experiments for each purified COQ protein. Dashed lines indicate where lanes have been moved and rearranged in the gel to ease visual comparison; original gels can be found in Supplementary Fig. 16.

Phylogenetic analyses across the chordate class within the metazoan kingdom were performed for all COQs (Supplementary Fig. 1). Each COQ protein exhibited phylogenetic trees in accordance with the species tree and ancestral proteins corresponding to the tetrapod ancestor (origin of four-limbed creatures, ≈350 Mya) were generated. The following ancestral COQs were resurrected: COQ3, COQ4, COQ5, COQ6, COQ7 and COQ9. The COQ8 paralogues, COQ8A and COQ8B, duplicated at the emergence of chordates (Supplementary Fig. 1) and therefore were both reconstructed. Each COQ sequence (3, 4, 5, 6, 7, 8A, 8B and 9) possesses an N-terminal mitochondria-targeting sequence that is cleaved once imported into the mitochondria. Mitofates was used for guidance to determine the site of N-terminal cleavage for expression in *Escherichia coli*^22^. Taking advantage of the Alphafold2 models, N-terminal truncations were further extended should large N-terminally disordered peptide remain. Indeed, COQ9 was truncated extensively (removal of 79 residues) in line with the construct used by Pagliarini and colleagues^23^. Each protein was successfully expressed and purified in *E. coli* (Extended Data Fig. 1). Unless otherwise stated, all the experimental work was performed using the tetrapod ancestral COQ proteins. They share >75% sequence identities with the corresponding extant human COQ proteins. Representative multiple sequence alignments for each COQ against a range of sequences corresponding to human, reptile and amphibia, to illustrate the extent of sequence conservation, are shown in Supplementary Fig. 2.

### C_5_ transformation requires a ferredoxin couple

**Extended Data Fig. 2 |:**
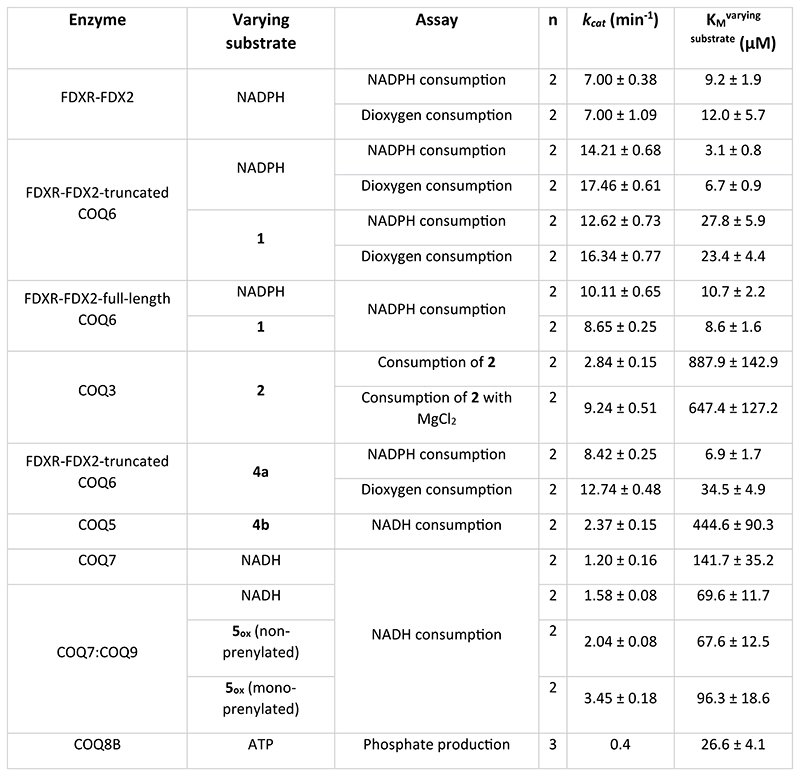
Kinetics ofthe COQ enzymes. Enzymatic parameters and the spectrophotometric assay employed, including the substrate measured, are reported in the table. K_M_ and *k_cat_* values have been determined by non-linear regression of n independent measurements with the Michaelis-Menten equation. n = 2 for all the experiments, except for the kinetics of COQ8B, where n = 3. All data are presented as best-fit value ± S.E.

**Fig. 1 |:**
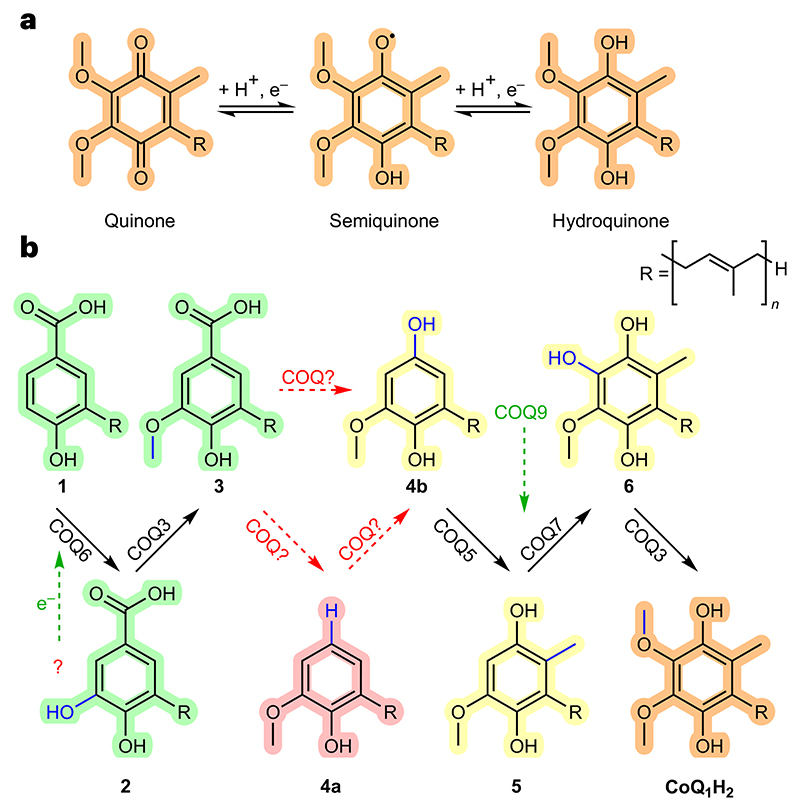
CoQ is a redox mediator. **a**, CoQ functions as one- and two-electron carrier. **b**, Its biosynthesis occurs at the interface between the inner mitochondrial membrane and the mitochondrial matrix through a series of head-group decorations. Unknown reactions in metazoan CoQ biosynthesis are depicted in red. Partners of COQ proteins are depicted in green. *p*-Hydroxybenzoic acid derivatives are highlighted in green, immature hydroquinones in yellow and the final product in orange. The hypothetic decarboxylated intermediate is highlighted in pink. 4-Hydroxy-3-(3-methylbut-2-en-1-yl)benzoic acid (also referred to as (poly)prenyl-hydroxybenzoic acid−(P) PHB) is named intermediate **1**; 3,4-dihydroxy-5-(3-methylbut-2-en-1-yl)benzoic acid (also referred to as (poly)prenyl-dihydroxybenzoic acid−(P)PDHB) is named intermediate **2**; 4-hydroxy-3-methoxy-5-(3-methylbut-2-en-1-yl)benzoic acid (also referred to as (poly)prenyl-vanillic acid−(P)PVA) is named intermediate **3**; 2-methoxy-6-(3-methylbut-2-en-1-yl)phenol is named intermediate **4a**; 2-methoxy-6-(3-methylbut-2-en-1-yl)benzene-1,4-diol (also referred to as demethyl-demethoxy-hydroquinone−DDMQ_n_H_2_) is named intermediate **4b**; 5-methoxy-2-methyl-3-(3-methylbut-2-en-1-yl)benzene-1,4-diol (also referred to as demethoxy-hydroquinone−DMQ_*n*_H_2_) is named intermediate **5**; 3-methoxy-6-methyl-5-(3-methylbut-2-en-1-yl)benzene-1,2,4-triol (also referred to as demethyl-hydroquinone−DMeQ_n_H_2_) is named intermediate **6**; 2,3-dimethoxy-5-methyl-6-(3-methylbut-2-en-1-yl)benzene-1,4-diol (also referred to as hydroquinone) is named CoQ_1_H_2_. The panel shows the intermediates in their reduced form. Several of them can adopt an oxidized quinone state (**a**) that can be generated enzymatically, by reaction with oxygen, or by spontaneous dismutation. The number of isoprene units *(n)* is 10 in humans, 8 in *E. coli*, 6 in *Saccharomyces cerevisiae* and 9 in *Arabidopsis thaliana*. In the present study the mono-prenylated versions of the intermediates were employed for reasons of solubility (*n* = 1).

COQ6, a class A flavin-dependent monooxygenase, represents the first protein in the CoQ head group biosynthetic pathway and performs C_5_ hydroxylation converting **1** to **2** (Fig. 1b)^24^. Full-length COQ6 was purified as a flavin adenine dinucleotide (FAD)-bound protein. However, it displayed low yields and poor FAD retention. We performed limited proteolysis using trypsin digestion to evaluate whether a more experimentally tractable construct could be obtained. Our analysis revealed that COQ6 was susceptible to a 35-residue C-terminal truncation that, when expressed, displayed higher yields, purified as a soluble protein, and retained the FAD cofactor (Supplementary Fig. 3). Importantly, the truncated enzyme exhibited similar kinetic properties to the full-length COQ6, suggesting that the C-terminus is not essential for activity (Extended Data Fig. 2). In the next paragraphs, we refer to the data obtained with this experimentally more tractable truncated COQ6.

**Extended Data Fig. 3 |:**
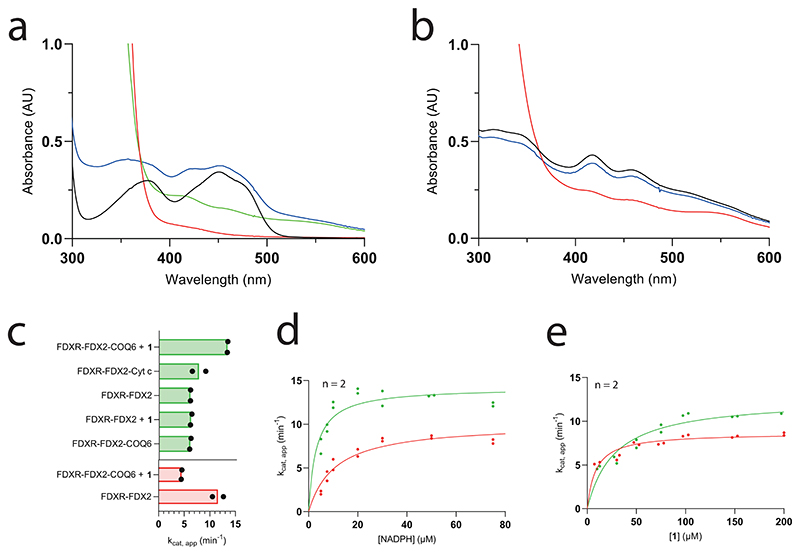
FDXR and FDX2 are required for COQ6 activity. **a**. Reduction-reoxidation assay of FDXR. UV/Vis spectra are shown in black at t = 0, in red after reduction with 10-fold excess of sodium dithionite, in green after the addition of an equimolar concentration of FDX2, in blue after 10 minutes of incubation. **b**. Reduction-reoxidation assay of FDX2. FDX2 UV/Vis spectra are shown in black at t = 0, in red after reduction with 10-fold excess of sodium dithionite, in blue after reoxidation by molecular oxygen recorded after 10 minutes of incubation. **c**. NADPH consumption (green) and cytochrome c reduction (red) assays probe the activities of functionally coupled FDXR, FDX2 and COQ6 combinations (mean of 2 independent experiments, individually plotted as dots). **d**. Comparing enzyme rates between full length COQ6 and C-terminally truncated COQ6: Michaelis-Menten kinetics of the FDXR-FDX2-COQ6 system in presence of an excess of **1** with NADPH as varying substrate (red) compared to the one obtained for FDXR-FDX2-C-terminally truncated COQ6 (green). **e**. Michaelis-Menten kinetics of the FDXR-FDX2-full length COQ6 system in presence of saturating concentration of NADPH with **1** as varying substrate (red) compared to FDXR-FDX2-C-terminally truncated COQ6 (green). Data points were collected using an NADPH spectrofluorimetric assay (Supplementary Fig. 4). Individual data points corresponding to n = 2 independent measurements are shown in panels d-e.

**Fig. 2 |:**
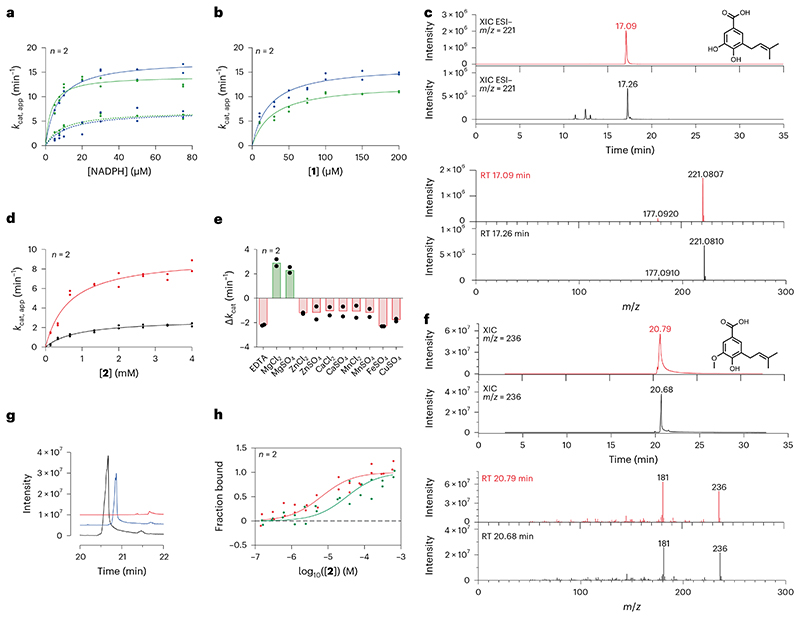
C_5_ decoration is catalysed by COQ6 and COQ3. **a**, Michaelis−Menten kinetics of the FDXR−FDX2 (dashed lines) and FDXR−FDX2−COQ6 (continuous lines) systems in presence of an excess of **1** and increasing NADPH concentrations. Rates were measured with both NADPH (green; Supplementary Fig. 4) and dioxygen (blue) consumption assays. **b**, Michaelis−Menten kinetics of the FDXR−FDX2−COQ6 system in the presence of saturating NADPH and increasing concentrations of **1**. **c**, UHPLC/HRMS analysis of the overnight COQ6 conversion of **1** to **2**. The XICs and ESI−full-scan mass spectra of **2** recorded after the injection of 500 ppm analytical standard and the reaction mixture are shown in red and black, respectively. The theoretical mass of the [M − H]^-^ molecular ion of **2** is 221.0808 Da. It was detected with an error of −0.45 and 0.90 ppm in the analyses of the standard and sample, respectively. **d**, Michaelis−Menten kinetics of COQ3 in the presence of an excess of SAM with **2** as varying substrate. Activity was measured in the presence (red) and absence of 150 μM MgCl_2_ (black). **e**, Bar graph showing the effect of 1 mM EDTA or 150 μM of divalent cations on COQ3 activity. The bars of the histogram show the mean value of independent replicates. **f**, GC/MS analysis of the overnight COQ3 conversion of **2** to **3**. The XICs and full-scan mass spectra of **3** recorded after the injection of 500 ppm analytical standard and of the reaction mixture are shown in red and black, respectively. **g**, Waterfall plot showing a qualitative analysis of the GC peak of **3** generated by COQ3 in the presence (black) or absence (blue−time offset 0.2 min) of 150 μM MgCl_2_ and in presence of 1mM EDTA (red−time offset 0.4 min). **h**, Microscale thermophoresis binding affinity curves of **2** binding by NHS-labelled COQ3 in presence (red) and absence (green) of 150 μM MgCl_2_. Individual data points corresponding to *n* = 2 independent measurements are shown in **a, b, d, e** and **h**. RT, retention time.

COQ6 was inspected for activity using **1** as substrate and NAD(P)H as reducing co-substrate. No activity was detected. We reasoned that COQ6 might function with an alternative electron donor. In yeast, it was recently demonstrated that Arh1, a ferredoxin reductase, and Yah1, a ferredoxin, are a prerequisite for CoQ biosynthesis and more importantly, Coq6 activity^25^. In line with these results, we postulated that the orthologues of this couple are also a requirement for the tetrapod species. The tetrapod ancestors of the mitochondrial ferredoxin reductase (FDXR) and ferredoxin 2 (FDX2, the orthologue for Yah1 (ref. 26)) were therefore resurrected (Supplementary Fig. 1). The two proteins were successfully isolated as soluble systems retaining their respective cofactors, FAD and [Fe_2_−S_2_], and inspected for activity. First, FDXR was reduced using sodium dithionite as an artificial reducing agent and its re-oxidation monitored overtime. Addition of equimolar quantities of FDX2 re-oxidized FDXR to completion within minutes corroborating its role as an electron acceptor (Extended Data Fig. 3a). Next, FDX2 was incubated with sodium dithionite to reduce the system and quench any oxygen in solution. Its re-oxidation was then monitored with time by adding oxygenated buffer (Extended Data Fig. 3b). These experiments proved that the FDXR/FDX2 couple is enzymatically active and allowed us to probe the turnover of NADPH (FDXR substrate) and molecular oxygen (FDX2 substrate) to produce reactive oxygen species. By monitoring either NADPH or molecular oxygen consumption, the reaction was found to follow Michaelis−Menten behaviour with a *k*_cat_ approaching 7.0 min^−1^ (Fig. 2a and Extended Data Fig. 2). Moreover, in the presence of both COQ6 and its substrate, **1**, the FDXR and FDX2 couple exhibited increased rates of NADPH and molecular oxygen consumption with a *k*_cat_ value of 14−17 min^−1^ (Fig. 2a and Supplementary Fig. 4) and a *K_M_* value for **1** of 23−28 μM (Fig. 2b and Extended Data Fig. 2). This experiment demonstrated that COQ6 can be reduced by FDX2. This process requires the presence of the substrate, a typical property of class A flavin-dependent monooxygenases^27^.

To confirm electron directionality and that FDX2 was transferring electrons to COQ6, we deployed a cytochrome c assay. FDXR and FDX2 alone reduced cytochrome c at a rate of 10 min^−1^ (Extended Data Fig. 3c). Addition of either COQ6 or **1** did not change the rate of reduction. However, the addition of both COQ6 and **1** together decreased cytochrome c reduction to approximately 4 min^−1^, implying that COQ6 competes with cytochrome c for the electron source (Extended Data Fig. 3c). To confirm that COQ6 was converting **1** into **2**, we applied ultrahigh-performance liquid chromatography coupled to high-resolution mass spectrometry (UHPLC/HRMS). Overnight incubation of COQ6 with FDXR, FDX2, FAD and an NADPH regeneration system at 30 °C, showed conversion of **1** to **2** (Fig. 2c). Conversely, in the absence of the ferredoxin couple no substrate conversion was detected. Hence, COQ6 activity, and the first reaction step in CoQ biosynthesis, is triggered by a coupled ferredoxin pair−FDXR and FDX2.

**Extended Data Fig. 4 |:**
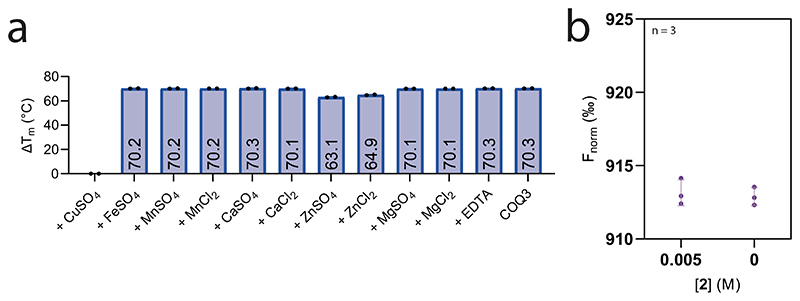
COQ3 requires Mg^2+^for substrate coordination. **a**. Unfolding temperature of COQ3 in the presence of a range of divalent cations (150 μM) or EDTA (1 mM). Individual data points corresponding to n = 2 independent measurements are shown. The bars of the histogram show the mean value of independent replicates. **b**. Microscale thermophoresis binding-check of NHS labelled COQ3 with **2** after pre-incubation with EDTA (1 mM). The substrate (**2**) does not cause any large difference in the normalised thermophoretic signal (the experiment without EDTA is shown in Fig. 2h). Individual data points corresponding to n = 3 independent measurements are shown. The error bars correspond to the standard deviations in n = 3 independent measurements for each datum.

The next step in the reaction pathway, **2** to **3**, concerns COQ3 (Fig. 1b). To determine whether COQ3 was active, we took advantage of the spectrophotometric signal of **2** (*Λ* = 430 nm, *ε* = 9.9 mM^−1^ cm^−1^) that disappears once methylated (Supplementary Fig. 5). Substrate depletion was observed in the presence of COQ3 and *S*-adenosyl methionine (SAM) and conveyed Michaelis−Menten kinetics with a *K*_M_ of 887.9 μM and a *k_cat_* of 2.8 min^-1^ (Fig. 2d and Extended Data Fig. 2). Known catechol *O*-methylases possess metal binding sites, including Mg^2+^, that can coordinate the incoming substrate in a bidentate fashion^28^. To verify whether COQ3 was metal dependent, we monitored rates of activity and melting temperatures in the presence of various divalent metals (Fig. 2e and Extended Data Fig. 4a). We did not observe any improved stability in the presence of divalent metals. However, supplementing MgCl_2_ or MgSO_4_ resulted in an approximate four-fold increase in activity and a reduction in the *K*_M_ by 27% for **2** (Fig. 2d,e and Extended Data Fig. 2). Addition of ethylenediamine tetraacetic acid (EDTA) abolished substrate turnover. Substrate transformation as well as the catalytic role of Mg^2+^ was also confirmed by gas chromatography/mass spectrometry (GC/MS) analysis after overnight incubation of COQ3 with **2** and SAM (Fig. 2f,g). Furthermore, using microscale thermophoresis, we found that the addition of MgCl_2_ decreases the *K*_d_ of **2** from 32.8 μM to 6.5 μM (80% decrease), substantiating a metal-based binding mode (Fig. 2h). EDTA abolished all extent of binding (Extended Data Fig. 4b). Hence, COQ6, activated by FDXR and FDX2, and COQ3 are responsible for the conversion steps at the C_5_ position.

### COQ4 and COQ6 are responsible for the unidentified steps

The enzymes responsible for the C_1_ modifications are currently unknown (Fig. 1b). In bacteria, a dedicated enzyme pair, UbiX and UbiD, catalyse aromatic decarboxylation and utilize the highly reactive prenylated flavin mononucleotide cofactor, prFMN^29,30^. Likewise, a dedicated enzyme, UbiH performs the hydroxylation at C_1_ (ref. 31). So far, no biochemically equivalent proteins have been found in animals, suggesting an alternative mode of function. We reasoned that, in animals, the biosynthetic pipeline could follow two possibilities: a hydroxylation-through-decarboxylation reaction that directly transforms **3** into **4b**, or a two-step process with one protein dedicated to the decarboxylation, **3** to **4a**, and one to the hydroxylation, **4a** to **4b** (Fig. 1b and Supplementary Fig. 6).

**Fig. 3 |:**
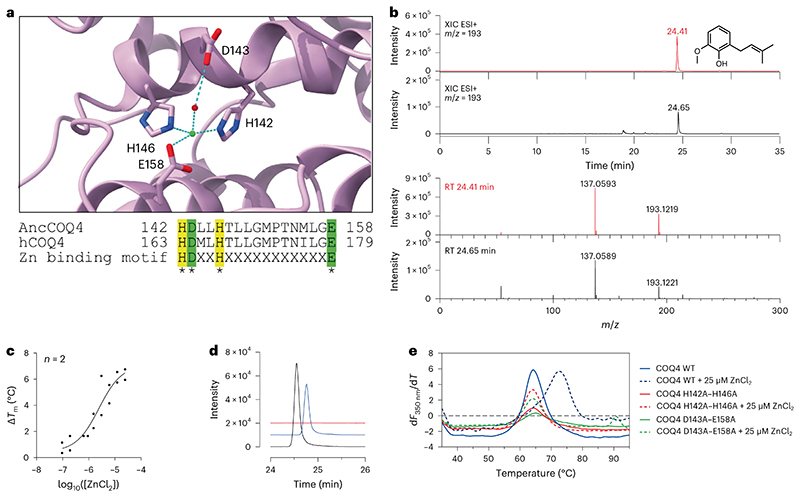
C_1_ decarboxylation is performed by COQ4 in a Zn^2+^-dependent manner. **a**, Close-up of the putative Zn^2+^-binding site in the predicted COQ4 AlphaFold model, and alignment of human and ancestral COQ4 with the Zn^2+^-binding motif highlighted. Residues involved are shown as sticks, and Zn^2+^ ion is shown in green and water molecules in red. **b**, UHPLC/HRMS analysis of the overnight COQ4 conversion of **3** into **4a**. The XICs and ESI+ full-scan mass spectra of **4a** recorded after the injection of 500 ppm analytical standard and of the reaction mixture are shown in red and black, respectively. The theoretical mass of the [M + H]^+^ molecular ion of **4a** is 193.1223 Da. It was detected with an error of −2.07 and −1.04 ppm in the analyses of the standard and sample, respectively. RT, retention time. **c**, Dose−response plot of the thermostabilizing effect provided by ZnCl_2_ on COQ4. **d**, Waterfall plot showing a qualitative analysis of UHPLC peak of **4a** produced by COQ4 in the presence (black) or absence (blue−time offset 0.2 min) of 25 μM ZnCl_2_ and in presence of 1mM EDTA (red−time offset 0.4 min). **e**, First derivative plot of nano-differential scanning fluorimetry analyses on COQ4 wild type (WT) and double mutants. The *T*_m_ corresponds to the peak of the first derivative. Individual data points corresponding to *n* = 2 independent measurements are shown in **c**.

Within the amidohydrolase superfamily there are Zn^2+^- and Mn^2+^-dependent *ortho*-(de)carboxylases that coordinate both the negatively charged carboxy and phenolate groups in a bidentate fashion for substrate decarboxylation^32,33^. Out of the currently known COQ proteins, COQ4 is the only system that possesses a canonical Zn-binding motif, HD−xx−H−(x)_11_−E (Fig. 3a), as first observed by Clarke and colleagues^34^. **3**, however, does not possess a hydroxy group at either the C_2_ or C_6_ position, *ortho* to the carboxy substituent. We hypothesized that the electron delocalization properties of the C_4_-hydroxy group, *para* to the carboxy moiety, in combination with the Lewis acidic properties of Zn^2+^, may nonetheless be able to facilitate the decarboxylation reaction. In line with *ortho*-(de)carboxylases that do not require co-substrates, COQ4 was incubated overnight with **3** at 30 °C. Remarkably, we found that COQ4 exercised decarboxylase activity and production of **4a** (Fig. 3b). No C_1_ hydroxylation, corresponding to **4b** production, was observed suggesting that animals perform two separate and subsequent biosynthetic transformations for C_1_ modification.

We obtained a half-maximal effective concentration value of 2.4 μM for COQ4 when titrating ZnCl_2_ (Fig. 3c). Furthermore, we observed higher turnover of **3** after supplementing 25 μM ZnCl_2_, which was commensurately abolished after adding 1 mM EDTA (Fig. 3d). To confirm Zn dependency over other divalent cations, we screened other potential salts; however, despite COQ4 being stabilized by other divalent cations, only ZnCl_2_ or ZnSO_4_ promoted activity (Extended Data Fig. 6a,b). To validate whether the metal contributes to enzymatic activity, we generated point mutants abolishing the metal binding site and exploited activity and thermostability analyses. Double-point mutants H142A−H146A and D143A−E158A failed to show any increased stability in the presence of ZnCl_2_ (Fig. 3a,e) and no product detected in UHPLC/HRMS analyses (Extended Data Fig. 6c). These findings attest to COQ4’s role as the C_1_ decarboxylase in CoQ biosynthesis. Delineating the chemical principles underlining this reaction mechanism will require further structural, computational and additional mutational studies. However, a potential reaction mechanism is proposed in line with amidohydrolase (de)carboxylases (Supplementary Fig. 6a)^32,33^.

**Fig. 4 |:**
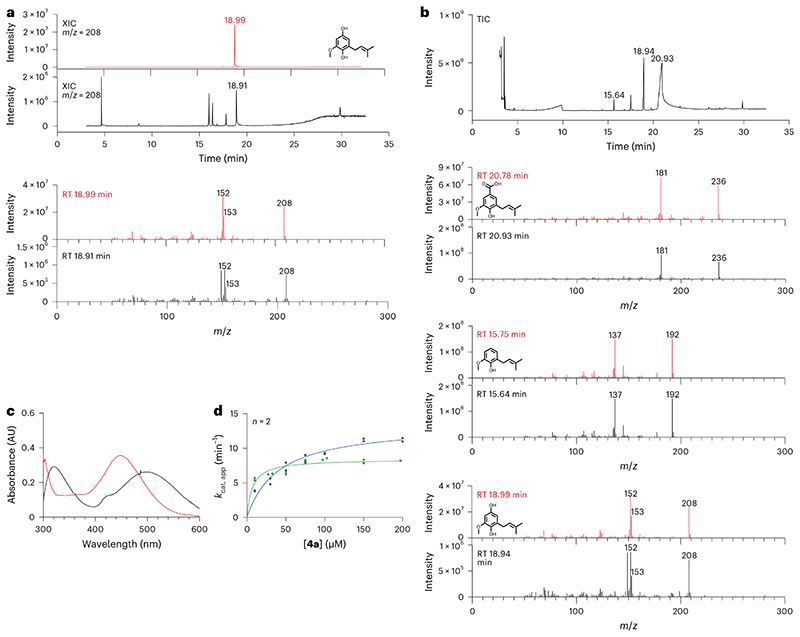
COQ6 is required for C_1_ hydroxylation. **a**, GC/MS analysis of the overnight COQ6 conversion of **4a** into **4b**. The XICs and full-scan mass spectra of **4b** recorded after the injection of 500 ppm analytical standard and of the reaction mixture are shown in red and black, respectively. The three unlabelled peaks in the reaction-mix chromatogram originate from the background (Supplementary Fig. 7a). **b**, GC/MS analysis of the overnight COQ6−COQ4 reaction of **3** to **4b**. The total-ion current (TIC) chromatogram recorded after the injection of the reaction mixture is shown. The full-scan mass spectra of **3, 4a** and **4b** recorded after the injection of a 500 ppm analytical standards (red) and of the mixture (black) are shown, respectively. **c**, Visible spectra of the 4-aminoantipyrine adducts produced by horseradish peroxidase after pre-incubation of **4a** with (red) and without (black) COQ6−FDXR−FDX2 and NADPH. **d**, Michaelis−Menten kinetics of FDXR−FDX2−COQ6 system in the presence of saturating concentration of NADPH with **4a** as varying substrate. Rates were measured with both NADPH (green) and dioxygen (blue) consumption assays. Individual data points corresponding to *n* = 2 independent measurements are shown in **d**.

With COQ4 demonstrating decarboxylase activity, we turned to the C_1_-hydroxylation step (Fig. 1b). We speculated that the reaction could be fulfilled by COQ6, considering its ability to perform aromatic hydroxylation. FDXR, FDX2 and COQ6 were incubated overnight at 30 °C with **4a** in the presence of an NADPH-regenerating system. We were able to detect product formation using GC/MS analysis, albeit at low levels, using both the truncated and full-length COQ6 proteins (Fig. 4a,b and Supplementary Fig. 7a). To substantiate the C_1_-hydroxylating role of COQ6, we exploited an in-house endpoint assay where the phenolic compounds, **4a** and **4b**, were conjugated to 4-aminoantipyrine using horseradish peroxidase^35^. The differing *Λ* of the adducts formed by **4a** and **4b** provided a qualitative assay to confirm product formation (Supplementary Fig. 7b). Indeed, COQ6, in the presence of FDXR and FDX2, produced the **4b** adduct corroborating its dual functionality as both a C_5_ and C_1_ hydroxylase (Fig. 4c). COQ6 displayed typical Michaelis−Menten kinetics at saturating concentrations of NADPH over a range of **4a** concentrations and exhibited molecular oxygen consumption in accordance with its role as a co-substrate and source for hydroxylation, with a *K*_M_ of 20 μM and a *k*_cat_ of 11 min^−1^ (Fig. 4d and Extended Data Fig. 2). COQ6 showed no activity in the presence of **3** and thus cannot function as a decarboxylase hydroxylase (see proposed reaction mechanism in Supplementary Fig. 6b). Finally, we found that coupling COQ6 with COQ4 resulted in the generation of both **4a** and **4b** starting from **3** (Fig. 4b). Critically, the detected amounts of **4b** were higher as compared with those obtained from COQ6 in isolation. This finding delineated a biosynthetic directionality and, more generally, demonstrated that the enzymes exert enhanced turnover when functionally coupled, a characteristic reminiscent of metabolons.

### C_2_ and C_6_ transformations require COQ5, COQ7 and COQ3

**Fig. 5 |:**
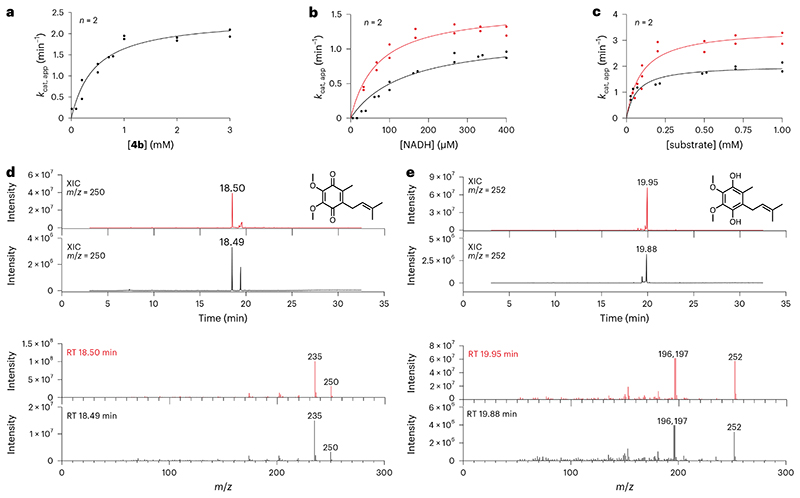
C_2_ decoration is catalysed by COQ5, COQ7 and COQ3. **a,** Michaelis−Menten kinetics of COQ5 in presence of an excess of SAM with **4b** as varying substrate. Activity was measured by coupling the enzyme to COQ7 and COQ9 to exploit the NADH consumption spectrofluorimetric assay. **b**, Michaelis−Menten kinetics of COQ7 in presence of an excess of non-prenylated 5_ox_ with NADH as substrate. Activity was measured in the presence (red) and absence of COQ9 (black). **c**, Michaelis−Menten curve of the COQ7−COQ9 system in presence of saturating concentration of NADH with **5_ox_** as substrate. Activities were measured using the mono-prenylated (red) and non-prenylated (black) version of the substrate. **d**, GC/MS analysis of the overnight COQ7−COQ9−COQ3 transformation of **5_ox_** to **CoQ_1_**. The XICs and full-scan mass spectra of CoQ_1_ recorded after the injection of 500 ppm analytical standard and the reaction mixture are shown in red and black, respectively. **e**, GC/MS analysis of the overnight COQ7−COQ9−COQ3 transformation of **5_ox_** to **CoQ_1_H_2_**. The XICs and full-scan mass spectra of **CoQ_1_H_2_** recorded after the injection of 500ppm analytical standard and the reaction mixture are shown in red and black, respectively. Individual data points corresponding to *n* = 2 independent measurements are shown in **a**−**c**. RT, retention time.

The remaining enzymatic steps include C_2_ methylation of **4b** by COQ5 and formation of the O-methyl group at the C_6_ position, mediated by COQ7 and COQ3, producing the final product, CoQ_1_ (ref. 36) (Fig. 1b). COQ5 was incubated overnight at 30 °C with SAM and its substrate, **4b**; GC/MS analysis confirmed enzymatic activity and production of **5** (Extended Data Fig. 6). We built an ad hoc enzyme-coupled spectro-fluorimetric assay consisting of COQ5 paired to an excess of COQ7 and COQ9. COQ5 showed typical Michaelis−Menten kinetics over titration of **4b** with a *k*_cat_ of 2.4 min^−1^ and a *K*_M_ of 444 μM (Fig. 5a and Extended Data Fig. 2). RT, retention time.

COQ7 is an NADH-dependent hydroxylase that possesses a carboxylate-bridged diiron centre and converts **5** to **6** (ref. 37). Previous work by Lippard and colleagues demonstrated that COQ7 (also referred to as clock-1, CLK-1,) hydroxylates the C_6_ position through a substrate-mediated reduction pathway, whereby NADH shuttles electrons to oxidized **5, 5_ox_** (the quinone form of **5**; see Fig. 1b legend), which then transfers electrons to the two Fe^3+^ centres^38^. To probe COQ7 activity, we monitored NADH depletion, in the presence of **5_ox_** or **5**, using ultraviolet/visible (UV/Vis) spectrophotometry. NADH depletion was detected only in the presence of **5_ox_** (non-prenylated) and exhibited Michaelis−Menten kinetics for NADH, with a *K*_M_ of 141.7 μM and a *k*_cat_ of 1.2 min^−1^, supporting Lippard’s mechanism of activation^38^ (Extended Data Fig. 2). COQ9 has been shown to facilitate COQ7 activity in vivo^38,39^. Consistently, addition of COQ9 increased catalytic efficiency by 1.5-fold, substantiating its role for COQ7 function (Fig. 5b and Extended Data Fig. 2). Furthermore, we note that the mono-prenylated substrate, **5_ox_**, increased enzyme velocity 1.5 times when compared with the non-prenylated equivalent (Fig. 5c and Extended Data Fig. 2).

COQ3 performs the final reaction step, **6** to CoQ_1_. However, due to issues of stability, we were unable to obtain **6**. Hence, we decided to synthesize it in situ and couple the reaction to COQ7 and COQ9.

Performing GC/MS analysis after overnight incubation at 30 °C exhibited consumption of **5_ox_** and formation of CoQ_1_ (both oxidised and reduced; Fig. 5d,e). These results demonstrated that each COQ protein is enzymatically competent in vitro and confirmed the dual functionality of both COQ3 and COQ6 for CoQ biosynthesis.

### In vitro reconstruction of CoQ biosynthesis

**Fig. 6 |:**
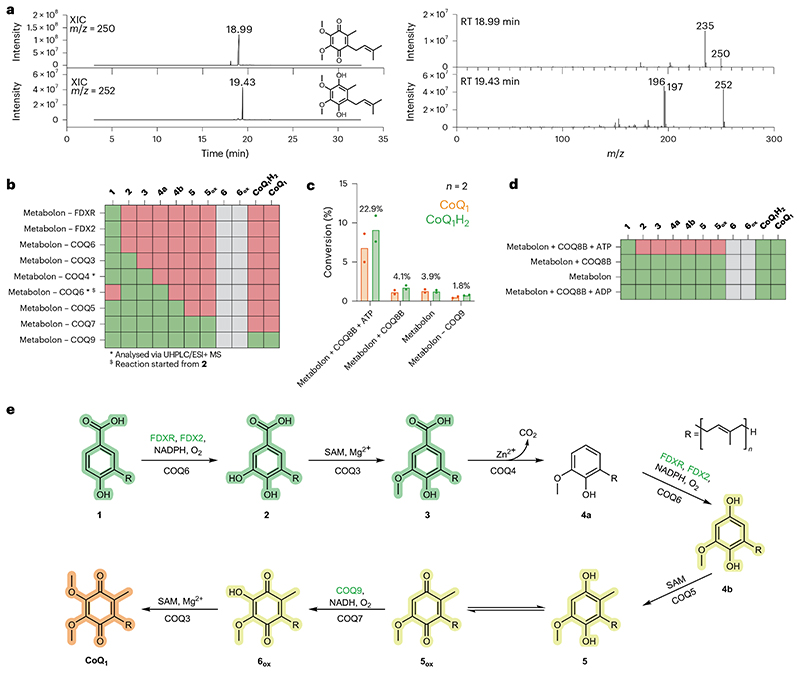
The in vitro reconstituted COQ metabolon is regulated COQ8B. **a**, GC/MS analysis of the overnight COQ metabolon transformation reaction of **1** to **CoQ_1_(H_2_)**. The XICs and full-scan mass spectra of **CoQ_1_** and **CoQ_1_H_2_** recorded from the reaction mixture are shown. XIC and full-scan mass spectra of 500 ppm analytical standard are reported in Fig. 5d,e. RT, retention time. **b**, Hit map showing detected (green) and undetected (red) CoQ_1_ biosynthesis intermediates after overnight COQ metabolon reaction. Intermediates for which an analytical standard was not available are depicted in grey. Individual COQ proteins or activators were removed one at a time (Supplementary Fig. 9). **c**, Percentage of conversion of **1** in **CoQ_1_**, either in the reduced (green) or oxidized (orange) form, in different conditions (Supplementary Figs. 10 and 11). **d**, Hit map showing the intermediates detected after conversion in the presence or absence of COQ8B and ATP or ADP. **e**, Vertebrate CoQ biosynthetic pathway in light of the findings of this work. *p*-Hydroxybenzoic acid derivatives are highlighted in green, immature hydroquinones in yellow and the final product in orange. COQ proteins responsible of each ring decoration step are reported below the arrow, co-substrates, metals and ancillary proteins (green) above. Individual data points corresponding to *n* = 2 independent measurements are shown in **c**. The bars of the histogram show the mean value of independent replicates.

With all COQs deemed enzymatically active, we turned to reconstructing the entire biosynthetic pathway. All COQs were pooled together, including several coenzyme regenerating systems and the initial substrate, **1**. Strikingly, the final product, CoQ_1_, was detected and illustrated the complete reconstitution of the CoQ biosynthetic pathway in vitro (Fig. 6a). Moreover, to evaluate whether the proteins are acting as stand-alone enzymes, or potentially engaging in protein−protein interactions, attesting to the formation of a COQ complex, we performed analytical size-exclusion chromatography, followed by Blue Native polyacrylamide gel electrophoresis (PAGE) and peptide mapping analyses, to assess whether they all co-elute. Fascinatingly, mixing all COQs−COQ3, COQ4, COQ5, COQ6, COQ7 and COQ9−resulted in the co-elution of all proteins as a monodisperse peak (Extended Data Fig. 7, Supplementary Fig. 8 and Supplementary Table 2). With the entire biosynthetic pathway obtained and evidence of the formation of the COQ metabolon, we explored the various intermediates obtained in the absence of various COQs. Initially, we assessed whether, in the absence of COQ6, **1** could be transformed by a downstream reaction, such as a C_1_ decarboxylation. Our GC/MS analysis illustrated that no intermediates possessing *m/z* values that could correspond to C_1_ decarboxylation, C_1_ hydroxylation, C_6_ hydroxylation, C_6_ O-methylation and C_2_ methylation, were present (Fig. 6b and Supplementary Fig. 9). This result suggests that CoQ biosynthesis is initiated by COQ6. By removing each of the subsequent COQ systems in turn, following the CoQ biosynthetic pathway, rewardingly, we observed the accumulation of the intermediate corresponding to the substrate of the absent COQ enzyme (Fig. 6b and Supplementary Fig. 9). Specifically, removing COQ6 and starting with the substrate of COQ3, **2**, led to the formation of **4a** and no production of **4b**. Conversely, removal of either COQ5, COQ7 or COQ9 (following C_1_ hydroxylation by COQ6), led to a clear accumulation of the C_1_-hydroxylated **4b** product with levels higher than those measured from the conversions by COQ6 in isolation (Fig. 4a and Supplementary Fig. 9g−i). This finding showcases the gain in efficiency afforded by coupling the enzymatic activities in a metabolon.

Collectively, these results show that, in vitro, the metabolic pathway has a firm directionality. However, we speculate that some flexibility in the order of the reactions may exist in vivo when cells are facing acute CoQ deficiency. It has been shown that yeast and mammalian cells harbouring deletions in COQ6 can still perform the C_1_ decarboxylation and hydroxylation steps independently of the C_5_ hydroxylation^24,40,41^. We speculate that another redundant hydroxylase might be able to perform C_1_ hydroxylation in these ΔCOQ6 cells. This hypothesis could explain why this step has resisted identification for decades.

### COQ8 imparts the streamlining capacity of the COQ metabolon

COQ8 is classified as an atypical kinase involved in CoQ biosynthesis^42–44^. It presents heightened ATPase activity in the presence of CoQ intermediates, cardiolipin and Triton X-100 and removal or inhibition of both paralogues, COQ8A and COQ8B, results in diminished CoQ production in humans^44^. To learn more about the role of COQ8, we scrutinized its function in CoQ biosynthesis. Ancestral COQ8A and COQ8B were purified as membrane-bound recombinant proteins; however, COQ8A produced very low yields compared with COQ8B. As such, we decided to experimentally characterize and scrutinize COQ8B’s specific role in CoQ biosynthesis.

We first sought out to assess whether COQ8B augments CoQ biosynthesis. COQ8B was added to the entire COQ biosynthetic pipeline in the presence and absence of an ATP regeneration system. Experiments were conducted using nucleotide concentrations (1 mM) matching physiologically relevant levels^45^. Size-exclusion chromatography showed that COQ8B does not efficiently co-elute with the metabolon. Fascinatingly, however, we observed an ATP-dependent increase in CoQ_1_ production (approximately five-fold) in the presence of COQ8B, reaching approximately 25% of turnover (Fig. 6c). The importance of ATP in CoQ biosynthesis is corroborated by past literature showing that deficiency in oxidative phosphorylation and subsequently decreased ATP levels impair CoQ biosynthesis^46^. Furthermore, we observed that, in the presence of COQ8B and ATP, no intermediate accumulation occurred (Fig. 6c,d and Supplementary Figs. 10 and 11). The data demonstrated that, though not an integral piece of the COQ metabolon, COQ8B ties up the system by coupling the transformations.

To elucidate the mode of operation underlining the benefit attributed by COQ8B, we first probed its catalytic properties. We observed a basal unspecific ATPase activity (*k*_cat_ of 0.4 min^−1^, *K*_M_ of 26.6 μM; Extended Data Fig. 2 and Supplementary Fig. 12) that was slightly increased by certain CoQ metabolites (up to two-fold for **1**) in line with previous work (Extended Data Fig. 8a)^43,44,47^. Critically, GC/MS analyses did not detect any phosphorylated CoQ intermediates, suggesting that the enzyme is not a small-molecule kinase.

We next asked whether COQ8B increases substrate turnover of the COQ enzymes. For these experiments, we selected COQ3, COQ6 and COQ7:COQ9 because their activities can be monitored by direct substrate consumption assays. None of them exhibited an increase in activity upon exposure to COQ8B and ATP (Extended Data Fig. 8b). However, we noticed that the activity of COQ6 was augmented approximately two-fold by the combined addition of COQ3 with COQ8B and ATP (Extended Data Fig. 8c). Likewise, the activity of COQ6 was similarly increased by the addition of COQ3 previously incubated with COQ8B and ATP. We therefore hypothesized that COQ8B could act as a COQ3 kinase. Intact protein MS validated this idea: COQ3, but not COQ6, is phosphorylated by COQ8B at multiple sites (Supplementary Fig. 13). This finding resonates with the work by Clarke and co-workers demonstrating that yeast mitochondrial Coq3 is phosphorylated^48^. Consistent with the increased activities for COQ6:COQ3, we noticed that this pair stimulates ATPase activity of COQ8B by ten-fold. Adding all COQ proteins associated to the COQ metabolon resulted in a similar extent of ATP hydrolysis. By contrast, when individually added, none of the COQ proteins increased the COQ8B ATPase activity (Extended Data Fig. 8a). Collectively, these results illustrate that COQ8B, while strictly not essential for the individual catalytic steps, fuels the overall biosynthetic process. We hypothesize that COQ8A and COQ8B might organize the metabolon structure. Substrate trafficking is thereby streamlined, attesting to the formation of the COQ metabolon (Fig. 6d). The molecular mechanisms underlying metabolon formation will be an exciting topic for future investigation, and we speculate that the assembly, and subsequent disassembly, of the metabolon, that collectively tunes CoQ output, is intricately controlled by both kinase, COQ8, and phosphatase, such as PPTC7, functions^49^.

## Discussion

In this work, to overcome hurdles associated to both COQ and CoQ intermediate stability, we employed ASR and took advantage of more soluble mono-prenylated CoQ intermediates. We speculate that, withinthe cell, protein/substrate chaperones, such as COQ10, may be essential for efficiently delivering the full-length intermediates as observed by Pierrel and colleagues when inspecting the COQ metabolon in *E. coli*^50^. Nevertheless, remarkably, this evolution-engineering approach successfully generated enzymatically competent enzymes, in line with current literature for animal CoQ biosynthesis, and conveyed key characteristics that support metabolon generation. The unknown reaction steps in CoQ biosynthesis were characterized, and the enzymes governing these transformations in animals were assigned (Fig. 6e). We do not see any major reason as to why this methodology could not be translated to other systems opening the way to the mechanistic and functional investigation of enzymes in the context of their pathways rather than as individual catalysts.

Metabolons are an assortment of proteins that collectively deliver the reactions of an entire metabolic pathway. They are commonly affiliated and pertain to reaction pathways that involve highly reactive intermediates. Over the course of evolution, with chemotaxis acting as the critical driving force^51^, they introduce beneficial protein-protein interactions, as illustrated by the co-elution of the large COQ complex. Large protein complexes typically suffer, or fail altogether, when integral proteins are removed from the assembly. Our results portray that, instead, the systems comprising metabolons are not so fragile per se since individual protein components are functional as stand-alone enzymes. Yet, only in the presence of their organizing partners and concomitant protein neighbours (for example, COQ8), are their enzymatic activities propelled and streamlined. This is the true essence of a metabolon−transitioning independently functional enzymes into a cohesive biosynthetic macromolecular machine.

## Methods

### Chemicals

All commercial chemicals and proteins for assays were purchased from Sigma-Aldrich, except for detergents that were purchased from Anatrace. 4-Hydroxy-3-(3-methylbut-2-en-1-yl)benzoic acid (**1**) was purchased from BLD Pharmaceutics. 3,4Dihydroxy-5-(3-methylbut-2-en-1-yl)benzoic acid (**2**), 4-hydroxy-3-methoxy-5-(3-methylbut-2-en-1-yl)benzoic acid (**3**), 2-methoxy-6-(3-methylbut-2-en-1-yl)phenol (**4a**), 2-methoxy-6-(3-methylbut-2-en-1-yl) benzene-1,4-diol (**4b**), 5-methoxy-2-methyl-3-(3-methylbut-2-en-1-yl) benzene-1,4-diol (**5**) and 5-methoxy-2-methyl-3-(3-methylbut-2-en-1-yl) cyclohexa-2,5-diene-1,4-dione (**5_ox_**) were purchased from WuXi App Tech. The purities (HPLC at 220 nm) are as follows: **2**, 99.50%; **3**, 99.00%; **4a**, 95.64%; **4b**, 98.20%; **5**, 85.65%; **5_ox_**, 88.28%.

### Phylogenetic inference and ASR

Human COQs sequences were employed as queries and for homology searches employing BLASTP. Datasets were constructed vetting all the chordates classes according to TimeTree^52^, by mining at least two species with fully sequenced genomes. Multiple sequence alignments were constructed in MAFFT v7 (ref. 53) and manually trimmed for single sequence insertions/terminal extensions. Neighbour-joining guide trees were constructed in MEGA v10.2 to assess the quality of datasets under construction. Once the working multiple-sequence alignments were obtained, best-fit substitution models and gamma distribution values (*α*) were calculated using ProtTest (data for each protein phylogeny are shown in Supplementary Fig. 1)^54^. Maximum likelihood phylogenies were inferred using RaxML v8.2.10 (HPC-PTHREADS module^55^), using rapid bootstrap analysis and searching for the best-scoring ML tree, with 500 bootstraps replicates and the given best-fit model under gamma distribution. When required, a species tree was used to constrain the phylogeny, this was constructed using TimeTree (timetree. org). Once the phylogenies were inferred, the bootstrap values were subjected to transfer bootstrap expectation values using BOOSTER online^56^. Figtree v1.4.2 was employed for analysing and visualizing the trees.

ASR was performed employing PAML v4.9a (CODEML module) as marginal reconstruction, using the phylogenies obtained previously, empirical amino acid substitution model (model =2) and either LG, JTT and DUMMY2 substitution matrices (Supplementary Fig. 1), four gamma categories and re-estimation of gamma shape parameter^57,58^. The distribution of the posterior probabilities (PP) for each of the ancestral states was analysed at the node corresponding to the tetrapod ancestor in each COQ phylogeny. Sites that displayed PP <0.8 were considered ambiguously reconstructed when alternative states displayed PP >0.2 (ref. 59). The length of the targeted nodes was treated by Fitch’s parsimony^57^. For reconstructed ancestral sequences, see Supplementary Fig. 14. To visualize the degree of sequence conservation across tetrapod organisms, multiple sequence alignments (MSAs) were generated using ESPript 3 (Supplementary Fig. 2)^60^.

### Size-exclusion chromatography

COQs were subjected to size-exclusion chromatography analyses to evaluate oligomeric profile and whether COQs exhibited aggregation. The samples were loaded onto an Äkta Pure system (Cytiva) equipped with either a Superdex 200 5/150 GL (Cytiva) or Superdex 200 10/300 GL (Cytiva) pre-equilibrated in storage buffer. Storage buffer consisted of 50 mM Tris−HCl pH 8.0 at 4 °C and 100 mM NaCl; COQs that were purified as insoluble proteins (COQ4, COQ5, COQ7 and COQ8B) were analysed using the same storage buffer supplemented with 0.03% N-Dodecyl-β-D-maltoside (DDM) (w/v final) (Anatrace, Anagrade).

COQs 3, 4, 5, 6, 7 and 9 were diluted to 1.5 μM in 1.3 ml of COQ-pulldown buffer: 50 mM Tris−HCl pH 8.0 at 4°C, and left mixing overnight at room temperature in the presence of geranyl-geraniol (substrate mimic, 250 μM) and coenzyme Q_10_ (1 μM). After overnight incubation, the sample was concentrated in an Amicon Ultra 0.5-ml centrifugal filter (Merck) with a 10 kDa cut-off until approximately 20 μl. The highly concentrated sample was then loaded onto an Äkta Pure system (Cytiva) equipped with a Superdex 200 5/150 GL (Cytiva) pre-equilibrated in 50 mM Tris−HCl pH 8.0 at 4 °C. The resulting elution was fractionated (0.1 ml), pooled together and concentrated using an Amicon Ultra 0.5-ml centrifugal filter (Merck) with a 10 kDa cut-off until approximately 20 μl. This sample was then then submitted for analyses using sodium dodecyl sulfate−PAGE and Blue Native PAGE (Extended Data Fig. 7 and Supplementary Fig. 8).

### FDXR−FDX2 kinetics with Cytochrome c reduction assay

The activity of FDXR−FDX2 enzymatic system was measured using 1 μM (final) of each protein, in 150 μl final volume of 50 mM Hepes pH 7.2, 250 mM NaCl, 10% v/v glycerol (Buffer A) with 50 μM FAD and 80 μM Cytochrome c (from bovine heart). These proteins were pre-incubated as a 50 μM stock for 30 min in ice beforehand. Reactions were monitored using 10.00-mm quartz cuvettes (Hellma) and a Cary 100 UV/ Vis spectrophotometer (Agilent) equipped with a thermo-stated cell holder (*T* = 25 °C). Reaction was started by adding 50 μM NADPH and monitored by following the reduction of Cytochrome c (*ε*_550 nm_ = 21.0 mM^−1^ cm^−1^). Activity was monitored in the presence and absence of 5 μM truncated COQ6 and 200 μM **1**. Superoxide dismutase was added to the reactions as a control.

### FDXR−FDX2−COQ6 kinetics with NADPH consumption assay

The activity of the FDXR−FDX2-truncated COQ6 enzymatic system was measured using 5 μM (final) of each protein, in a 200 μl final volume of Buffer A with 50 μM FAD added. These proteins were pre-incubated as a 50 μM stock for 30 min in ice beforehand. Reactions were monitored using 10.00-mm quartz cuvettes (Hellma) and a Cary Eclipse fluorescence spectrophotometer (Agilent) equipped with a thermo-stated cell holder (*T* = 25 °C). Reaction was started by adding NADPH and rates were determined by following NADPH oxidation (excitation 340 nm, emission 460 nm). A calibration line was previously built by measuring the fluorescence at known NADPH concentrations. The activity of the FDXR−FDX2−COQ6 full-length enzymatic system was measured following the same experimental procedure and supplementing 0.05% DDM (w/v, final) to the assay buffer. *K*_M_ of NADPH was with 200 μM of **1** as substrate. *K*_M_ of **1** and **4a** were determined using 50 μM NADPH. GraphPad Prism 9 was used to perform non-linear regression. Activity was measured also in the presence of 1 μM COQ8B, 200 μM MgCl_2_ with 10 μM ATP and in the presence of 5 μM COQ3 and 200 μM MgCl_2_ in a series of conditions: (1) without any addition, (2) with 1 μM COQ8B and 10 μM ATP, (3) with 1 μM COQ8B, (4) 10 μM ATP and 100 μM adenosine 5’-(β,γ-imido)triphosphate (AMP-PMP) or (5) with 5 μM COQ3 phosphorylated (overnight incubation with 1 μM COQ8B and 1 mM ATP, after size-exclusion chromatography).

### FDXR−FDX2−COQ6 kinetics with dioxygen consumption assay

The activity of the FDXR−FDX2−truncated COQ6 system was measured using 1−5 μM of proteins prepared as above, in 1 ml final volume of Buffer A with 50 μM FAD using a Hansatech Oxygraph instrument (Hansatech Instruments). Before measurements the instrument was first calibrated to determine the zero-dioxygen level by complete reduction with sodium dithionite. Reaction was started by adding NADPH. Rates were determined measuring the dioxygen consumption. Activity of FDXR−FDX2−COQ6 full length could not be measured due to bubble formation from the stirred detergent solution interfering with the oxygen sensor. *K*_M_ of NADPH was determined with 200 μM of **1** as substrate. *K*_M_ of **1** and **4a** were determined using 50 μM NADPH. GraphPad Prism 9 was used to perform non-linear regression.

### COQ3 kinetics with direct spectrophotometric assay

The activity of COQ3 was monitored using 1 μM protein in 150 μl final volume of Buffer A with 2 mM SAM, with 10.00-mm quartz cuvettes (Hellma) and a Cary 100 UV/Vis spectrophotometer (Agilent) equipped with a thermo-stated cell holder (*T* = 25 °C). The reaction was started by adding **2**, and rates were monitored by following the decrease of the absorbance at 430 nm. The *ε*_430 nm_ value of 9.9 mM^−1^ cm^−1^ was determined plotting the absorbance at 430 nm of **2** at known concentrations in a calibration line. The spectra of the standard samples were recorded using a Diode Array 8453 UV/Vis Spectrophotometer (Agilent). Activity was tested for the stand-alone protein, in the presence of 150 μM CuSO_4_, FeSO_4_, MnSO_4_, MnCl_2_, CaSO_4_, CaCl_2_, ZnSO_4_, ZnCl_2_, MgSO_4_ and MgCl_2_ and 1 mM EDTA as control. *K*_M_ of **2** was determined in presence and absence of 150 μM MgCl_2_. GraphPad Prism 9 was used to perform non-linear regression. Activity was measured also in the presence of 1 μM COQ8B, 200 μM MgCl_2_ and 10 μM ATP.

### COQ7−COQ9 kinetics with NADH consumption assay

The activity of COQ7 and of the COQ7−COQ9 complex was measured using 5 μM protein in 150 μl final volume of Buffer A with 0.05% DDM (w/v, final) with 10.00-mm quartz cuvettes (Hellma) and a Cary 100 UV/ Vis spectrophotometer (Agilent) equipped with a thermo-stated cell holder (*T* = 25 °C). COQ7 and COQ9 were pre-incubated on ice at 5 μM each for 30 min. Reaction was started by adding NADH, and rates were determined by following NADH oxidation (*ε*_340 nm_ = 6.22 mM^-1^ cm^-1^). *K*_M_ of NADH was determined using 700 μM **5_ox_** (non-prenylated) as substrate. The *K*_M_ of **5** and **5_ox_** were determined using 500 μM NADH. GraphPad Prism 9 was used to perform non-linear regression. Activity of COQ7 was also measured in the presence of 1 μM COQ8B, 5 μM COQ9, 200 μM MgCl_2_ and 10 μM ATP.

### COQ5 kinetics coupled to COQ7−COQ9 with NADH consumption assay

The activity of COQ5 was measured by coupling it to the reaction of COQ7. Reaction mixture (200 μl) contained 5 μM COQ5, 25 μM COQ7, 25 μM COQ9, 1 mM SAM, 150 μM NADH and 0.05−3 mM **4b**. Reactions were monitored using 10-mm quartz cuvettes (Hellma) and a Cary Eclipse fluorescence spectrophotometer (Agilent) equipped with a thermo-stated cell holder (25 °C). All reagents except SAM and NADH were blanked. Reaction was started by adding SAM, and rates were determined by following NADPH oxidation (excitation 340 nm, emission 460 nm). Adequate controls were performed by removing single reagents from the mixture. GraphPad Prism 9 was used to perform non-linear regression.

### Small-scale reactions

Reactions with single COQ proteins and with the COQ metabolon were carried out overnight on a 400-μl scale in brown 1.5-ml Eppendorf tubes in a benchtop incubator at 30 °C and 200 rpm. The reaction mixture contained 5 μM proteins, 5 mM substrate from a 100 mM stock in absolute ethanol, and reaction-dependent cofactors/metals as follows: reaction of **1** contained COQ6, FDXR, FDX2, 250 μM FAD and NADPH regeneration system (1.2 U glucose dehydrogenase and 1.2 mM glucose); conversion of **2** contained COQ3, 5 mM SAM, 150 μM MgCl_2_ and 0.05% DDM (w/v, final); reaction of **3** contained COQ4−GST, 25 μM ZnCl_2_ and 0.05% DDM (w/v, final); conversion of **4a** contained COQ6, FDXR, FDX2, 250 μM FAD and NADPH regeneration system; conversion of **4b** contained COQ5, 5 mM SAM and 0.05% DDM (w/v, final); reaction of **5_ox_** contained COQ3, COQ7, COQ9, 150 μM MgCl_2_, 5 mM SAM, 0.05% DDM (w/v, final) and NADH regeneration system. Adequate controls were performed by removing single components from the reaction mixture. The reactions of COQ3 and COQ4 were performed also in the presence of 1 mM EDTA as a negative control. The reaction of **3** by COQ4 was also tested in the presence of 25 μM CuSO_4_, FeSO_4_, MnSO_4_, MnCl_2_ and ZnSO_4_. It was also tested utilizing COQ4 H142A−H146A and D143A−E158A mutants. The reaction of **1** to CoQj was tested by adding all the COQ proteins and cofactors/metals as described for individual reactions. The reaction of **1** with the COQ metabolon was tested in presence of COQ8B with and without an ATP regeneration system, in the absence of COQ8B and by removing individual proteins as a control. The reaction of **2** to CoQ_1_ was also tested in the absence of COQ6 as a control. As NAD(P)H regeneration system, 300 μM NAD^+^ and NADP^+^, 1.2 U glucose dehydrogenase and 1.2mM glucose were used. As ATP regeneration system, 1 mM ADP, 4 U pyruvate kinase, 5 mM phosphate and 5 mM phosphoenolpyruvate were used. Reactions were started by adding the substrate. Reactions, except for the reactions of **1** to **2** and **3** to **4a**, were quenched by adding an equal volume of ethyl acetate and vortexed. Organic and aqueous phases were separated by centrifugation at 20,000*g* for 10 min at room temperature. The organic phase was submitted to GC/MS analysis. Reactions of COQ6 with **1** and COQ4 were quenched by adding acetonitrile 1:3, vortexed and incubated in ice for 10 min. Proteins were precipitated by centrifugation at 20,000*g* for 10 min at room temperature. The supernatant was submitted to UHPLC/HRMS analysis. The reaction experiments on the entire COQ metabolon were analysed with both analytical methods.

For GC/MS, samples (**1, 2, 3, 4a, 4b, 5, 5_ox_**, CoQ_1_ and CoQ_1_H_2_) were analysed by GC/MS on a DSQII single quadrupole system (Thermo Scientific) coupled to a Trace GC system (Thermo Scientific) equipped with a Rxi-5ms (30 m × 0.25 mm × 0.25 μm inner diameter) capillary column (Restek), with helium as carrier gas at a constant 1 ml min^−1^ flow rate. The injection was performed in split-less mode as follows: split-less time 1 min, injection volume 1 μl and injection temperature 250 °C. The GC oven temperature was held at 45 °C for 2 min, linearly increased to 300 °C at a rate of 10 °C min^−1^ and held at 300 °C for 5 min. The transfer line temperature was set at 290 °C and the ion source temperature at 250 °C. A qualitative analysis was carried out by overlaying retention times and mass spectra with that of standards representing one of the various intermediates recorded at 500 ppm dissolved in absolute ethanol.

A quantitative analysis was performed to determine the CoQ_1_(H_2_) content in the reaction with the intact COQ metabolon and controls. Standard solutions were prepared in 100% ethyl acetate. The calibration range was 10−100 μM, *R^2^* > 0.98. Standard solutions were injected in duplicate as described above following a blank injection of ethyl acetate. Samples were diluted ten-fold before injection. Quantitation of the analyte was performed according to external calibration curves interpolated with the quadratic regression model (CoQ_1_) and linear (CoQ_1_H_2_) (Supplementary Fig. 11). CoQ_1_H_2_ was quantitated utilizing the extracted ion chromatogram (XIC) of *m/z* 252, CoQ_1_ was quantitated utilizing the XIC of *m/z* 250 + 235. Percentage of bioconversion was calculated as ratio between the product of the calculated concentration of CoQ_1_(H_2_) with the dilution factor and the initial concentration of **1**.

## UHPLC/HRMS

C_5_ hydroxylation was monitored in UHPLC/HRMS in negative polarity as **1** and **2** were almost co-eluting in GC/MS (for example, see Supplementary Fig. 9g). We also observed a minimal decarboxylation of the Cj-carboxyl moiety in **1, 2** and **3** once submitted to GC/MS analyses, most likely due to the high temperature of the GC inlet (250 °C)^61^. For this reason, we applied UHPLC coupled to electrospray ionization quadrupole time-of-flight high resolution mass spectrometry (ESI−qTOF-HRMS) to identify the C_1_-decarboxylated intermediate (**4a**). Samples were analysed by ESI−qTOF-HRMS on a X500B QTOF system (SCIEX) equipped with the Twin Sprayer ESI probe coupled to an ExionLC system (SCIEX) controlled by SCIEX OS software 3.0.0. Injection volume was 10 μl. Chromatographic separation was carried out with a Kinetex EVO C18 (100 mm length × 2.1 mm diameter, 2.6 μm particle size; Phenomenex). The mobile phase consisted of water (A) and acetonitrile (B) both including 0.1% (v/v) formic acid. Flow rate was set at 0.2 ml min^−1^. Gradient elution was performed as follows: 2% B at 0.0−0.1 min, 2−66% B at 0.1−32.0 min, 66-2% B at 32.0−35.0 min. MS detection parameters were set as follows: curtain gas 30 psi, ion source gas 1 45 psi, ion source gas 2 55 psi, temperature 450 °C. The full scan range of *m/z*50−1,000 was monitored in negative mode for **1** and **2**, with spray voltage of −4,500 V, de-clustering potential of −60 V and collision energy of −10 V, or in positive mode for **1, 2, 3** and **4a**, with spray voltage of +5,500 V, de-clustering potential of +50 V and collision energy of +10 V. Mass calibration was performed with the ESI Negative Calibration solution or the ESI Positive Calibration solution for the SCIEX X500 system (SCIEX) before experiments. A qualitative analysis was carried out by overlaying retention times and mass spectra with standards representing one of the various intermediates recorded at 50 ppm and dissolved in absolute ethanol.

### Nuclear magnetic resonance

Compounds were dissolved in deuterated DMSO (d_6_-DMSO), or deuterated chloroform (CDCl_3_) and spectra were recorded with 100 scans on a Bruker 400 MHz Avance III instrument and analysed using TopSpin 4.3.0. Supplementary Fig. 15 reports on the spectra.

### Reproducibility and statistics

All experimental observations were confirmed by fully independent repeat experiments. The kinetics experiments were performed in duplicate. The endpoint activity assays, and the analytical chemistry experiments were performed in triplicate. Unless stated otherwise, the mean of numerical data is shown, with individual data points shown for *n* < 3, where *n* is the number of replicates. Statistics were performed using GraphPad Prism version 9. Supplementary Fig. 16 shows the unprocessed gels.

### Reporting summary

Further information on research design is available in the Nature Portfolio Reporting Summary linked to this article.

## Extended Data

**Extended Data Fig. 5 |:**
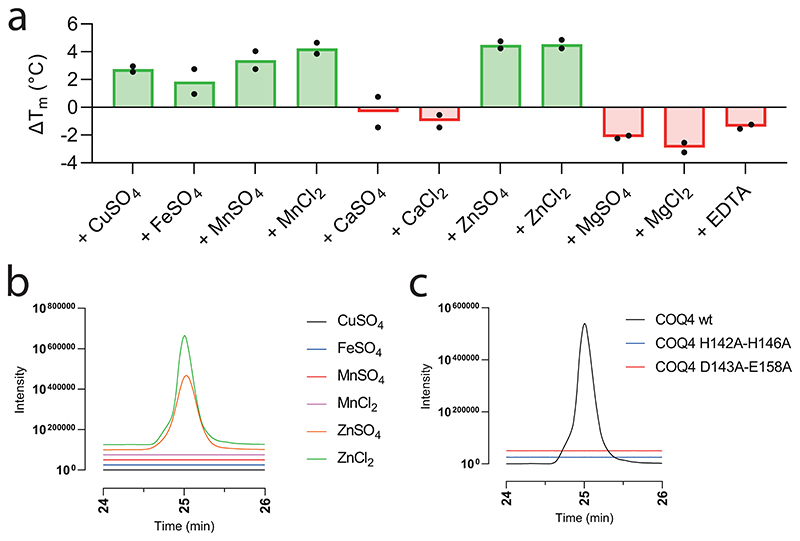
Zn^2+^ promotes decarboxylase activity of COQ4. **a**. Melting temperature of COQ4 in the presence of a range of divalent cations (25 μM) or EDTA (1 mM). Individual data points corresponding to n = 2 independent measurements are shown. The bars of the histogram show the mean value of independent replicates. **b**. Qualitative analysis of the UHPLC peak of **4a** after overnight conversion of 3 by COQ4 in the presence of the divalent cations (25 μM) that showed a thermal stabilization. **c**. Qualitative analysis of the UHPLC peak of 4a after overnight conversion of 3 by COQ4 double point mutants in the presence of ZnCl_2_ (25 μM) in comparison with wild-type COQ4.

**Extended Data Fig. 6 |:**
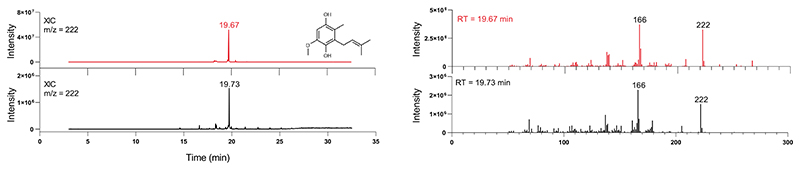
COQ5 C_2_-methylase activity is confirmed by GC/MS. GC/MS analysis of the overnight COQ5 reaction of **4b** into **5**. The extracted-ion chromatograms and full-scan mass spectra of **5** recorded after the injection of 500 ppm analytical standard and the reaction mixture are shown in red and black, respectively. RT, retention time.

**Extended Data Fig. 7 |:**
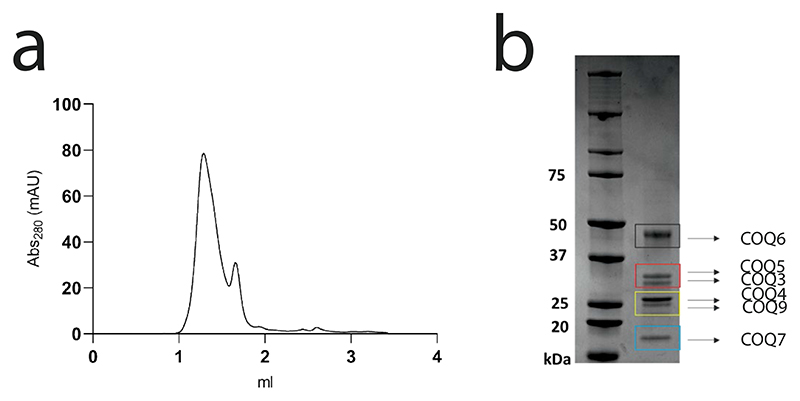
A large COQ complex can be reconstituted *in-vitro*. **a**. Analytical Size Exclusion Chromatography profile. The protein mix (derived from individually purified COQ proteins) was pre-incubated with both CoQ_10_ and geranyl-geraniol, a mimic of the poly-isoprene tail, as it gave rise to a sharper gel filtration peak. **b**. SDS-PAGE of the pooled and concentrated fractions corresponding to peak 1. Size Exclusion Chromatography and SDS-PAGE analyses were repeated in n = 3 independent experiments. See also Supplementary Figs. 8 and 16.

**Extended Data Fig. 8 |:**
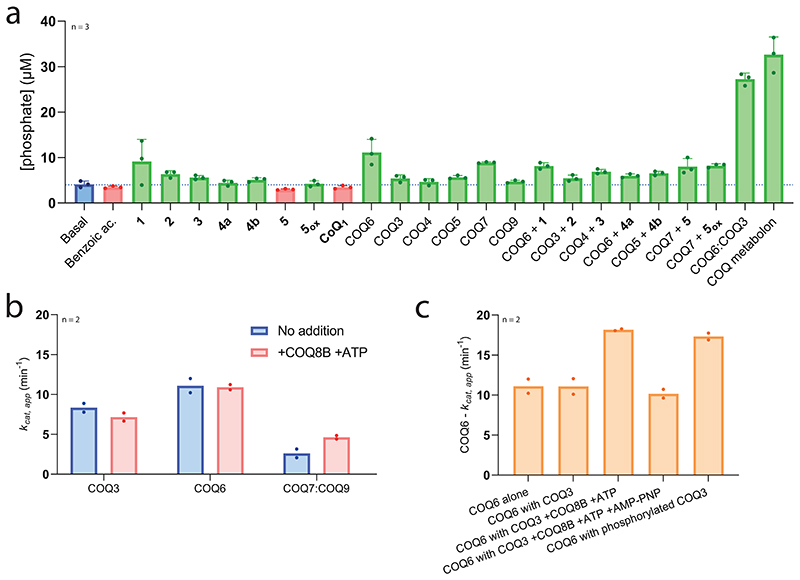
Dissecting the role of COQ8B in CoQ biosynthesis. **a**. COQ8B ATPase activity is stimulated by COQ proteins and intermediates. Micromolar concentration of inorganic phosphate released via ATP hydrolysis by COQ8B in a series of conditions. Concentrations were determined after 10 minutes following a calibration line of analytical standard of phosphate reacted with the Malachite green dye (Supplementary Fig. 12). The phosphate concentration produced by COQ8B in the absence of any COQ substrate or protein (*ca*. 4 μM) is shown as a blue bar and dashed line. Individual data points corresponding to n = 3 independent measurements are shown. The bars of the histogram show the mean value of independent replicates. The error bars correspond to the standard deviations in n = 3 independent measurements for each datum. **b**. COQ8B promotes activity for paired COQ proteins. COQ8B (1 μM) and ATP (10 μM) does not boost COQ3 and COQ6 activity, and marginally for COQ7:COQ9 as measured by substrate consumption. **c**. COQ6 activity is boosted by COQ8B and ATP only if in the presence of COQ3. As a further control, the role of COQ8B was evaluated by competition of ATP with AMP-PNP (Adenosine 5’-(β,γ-imido)triphosphate; 100 μM), a non-hydrolysable ATP analogue. COQ3, previously phosphorylated by pre-incubation with COQ8B and ATP, provided a comparable increase in COQ6 activity. Phosphorylated COQ3 was generated by incubation with COQ8B, and ATP followed by size-exclusion chromatography purification. Individual data points corresponding to n = 2 independent measurements are shown in panels b-c. The bars of the histogram show the mean value of independent replicates.

## Supplementary Material

Source Data

Supplementary Data 1

Supplementary file

## Data Availability

The experimental data generated in this study is provided in the supplementary information and source data files. All other data are available from the authors upon reasonable request. The ancestral sequences generated in this study can be found in Supplementary Information and the resulting genes used in this study (possessing N-terminally truncated motifs) have been submitted for deposition in the Genbank database. The accession codes for each ancestral protein are listed here and found in Supplementary Fig. 14: tAncCOQ3_tr: OQ859710; tAncCOQ4_tr: OQ859711; tAncCOQ5_tr: OQ859712; tAncCOQ6_tr: OQ859713; tAncCOQ7_tr: OQ859714; tAncCOQ8A_tr: OQ859715; tAnc-COQ8B_tr: OQ859716; tAncCOQ9_tr_N79: OQ859717; tAncFDXR_tr: OQ859718; tAncFDX2_tr: OQ859719. The collected dataset for the phylogenetic analysis is provided in Supplementary Information. The taxonomic relationships and evolutionary timescale data used in this study are available in the TimeTree 5 knowledge-base (http://www.timetree.org/). Source data are provided with this paper.
