# Supplementary figures and images for: In vitro construction of the COQ metabolon unveils the molecular determinants of coenzyme Q biosynthesis
Source: Nat Catal. Author manuscript; Available in PMC 2024 Feb 29. (PMC7615680; doi:10.1038/s41929-023-01087-z)

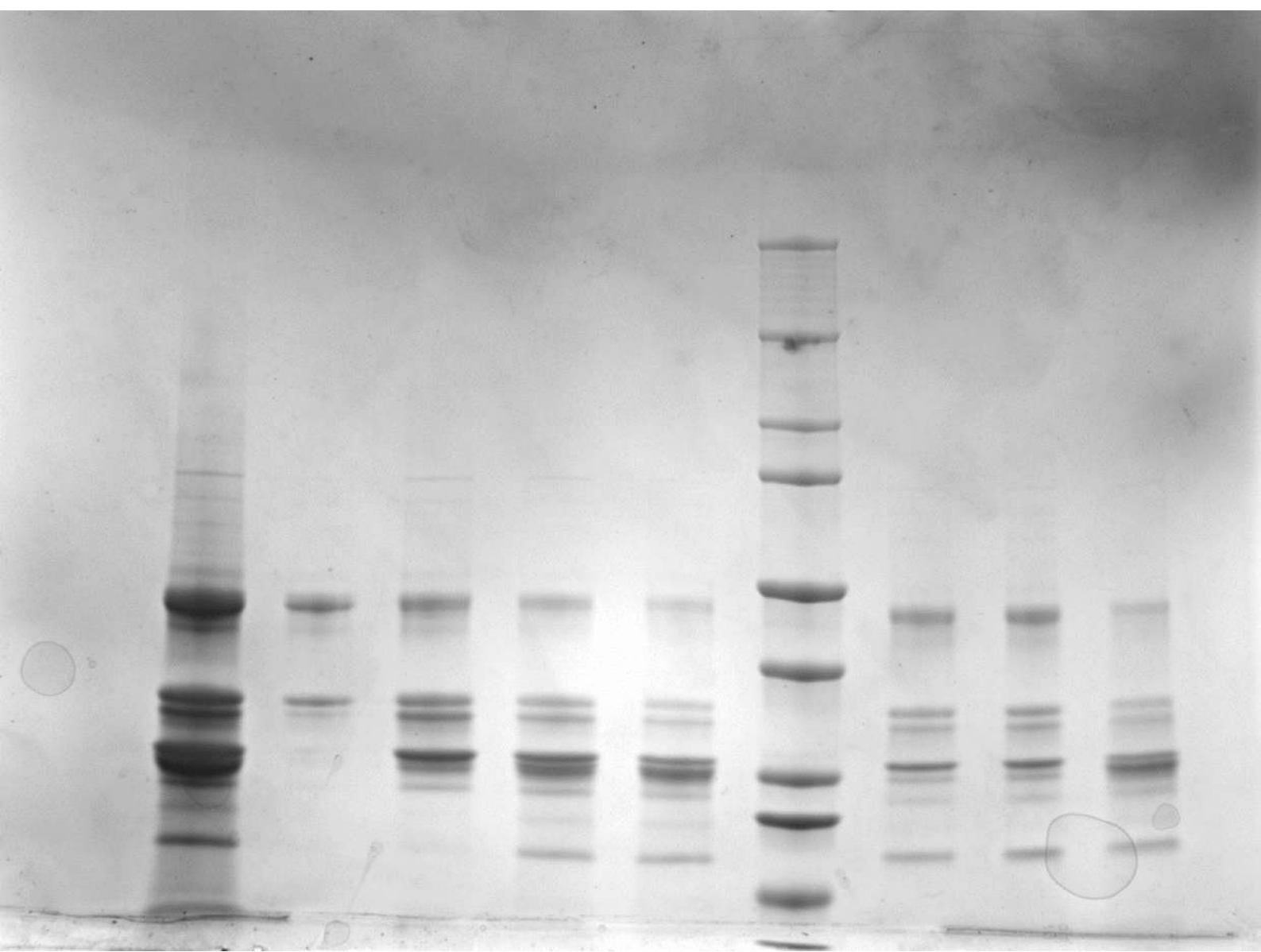

Supplement: Source Data [file EMS193170-supplement-Source_Data.zip › Source Data/41929_2023_1087_MOESM15_ESM.pdf]

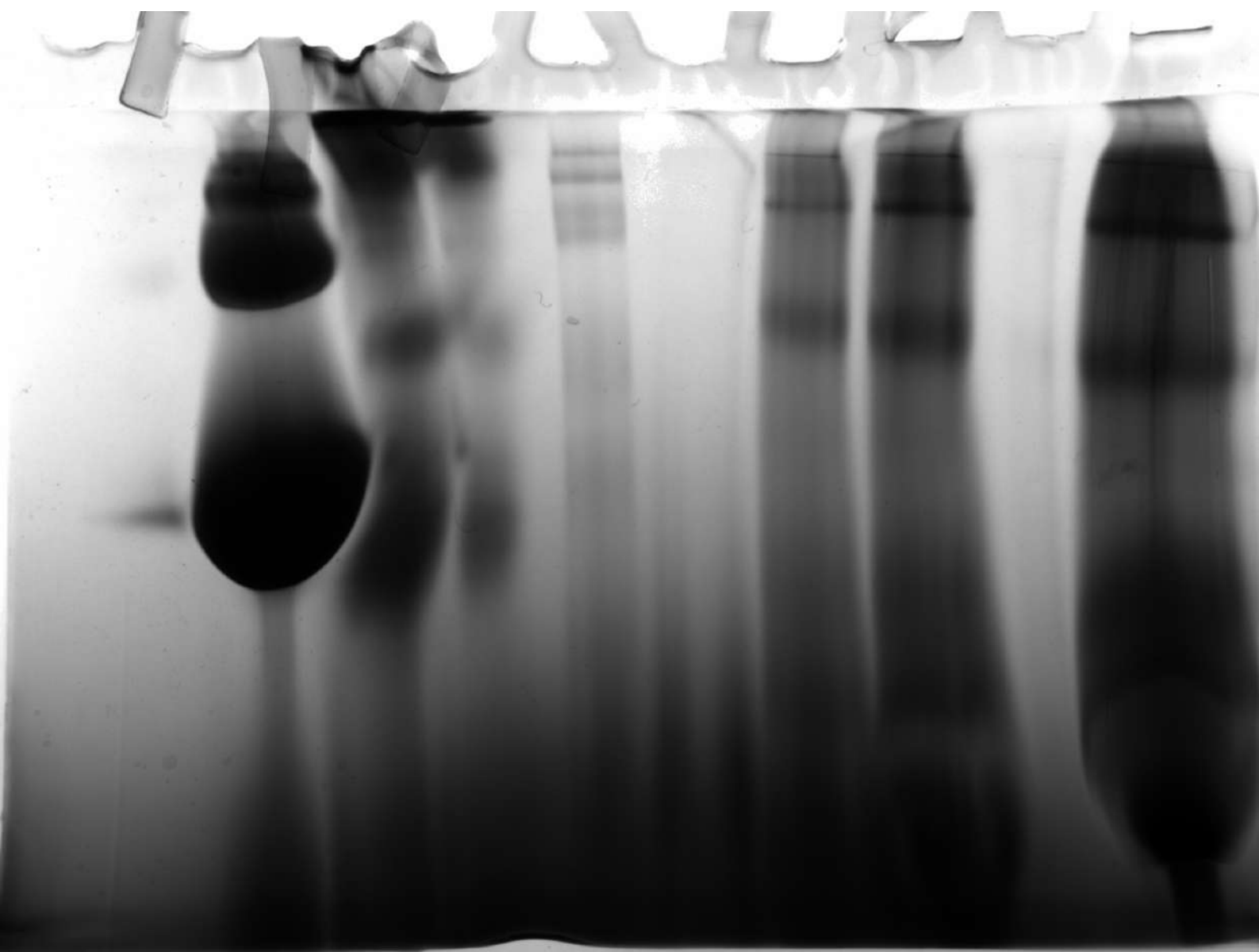

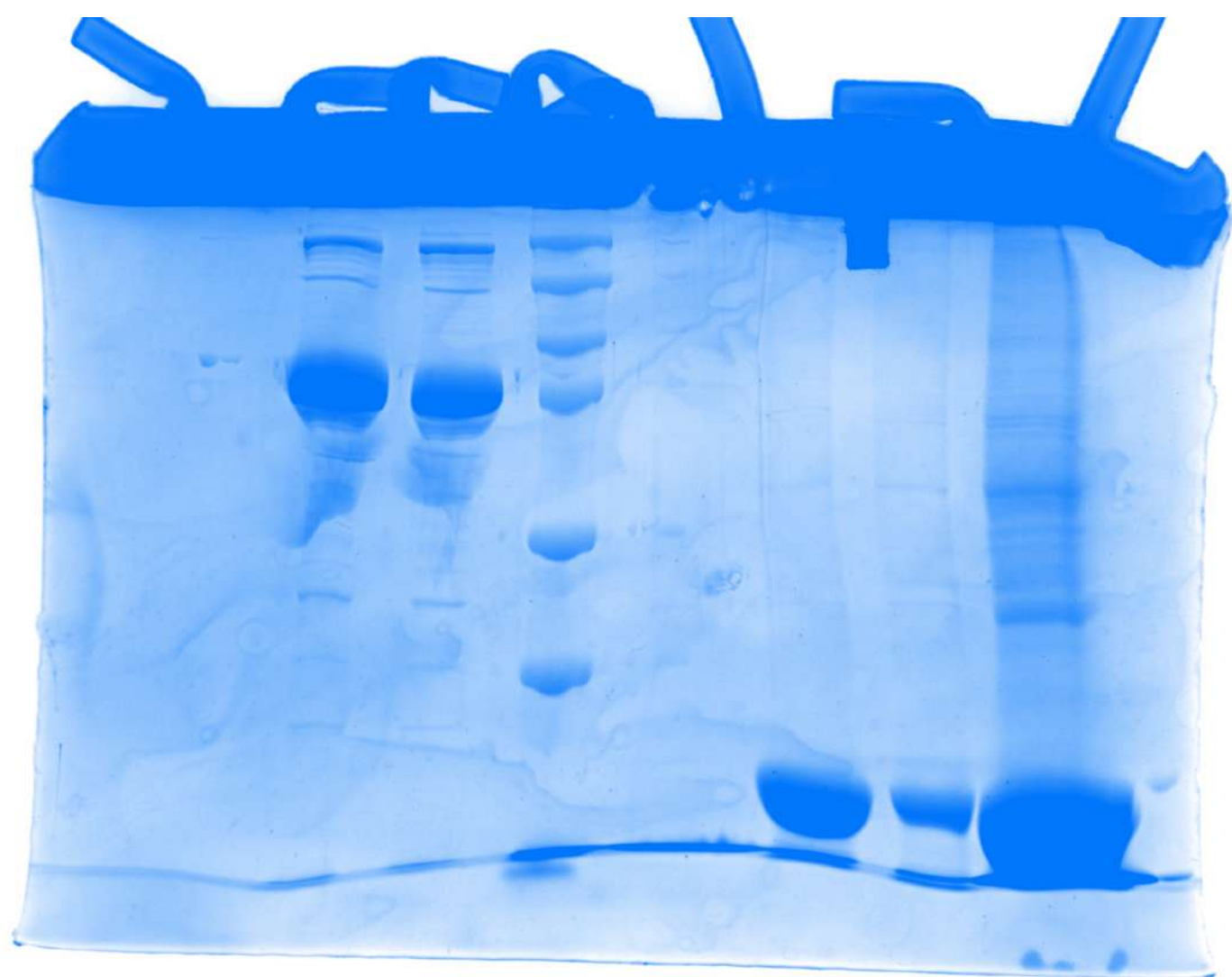

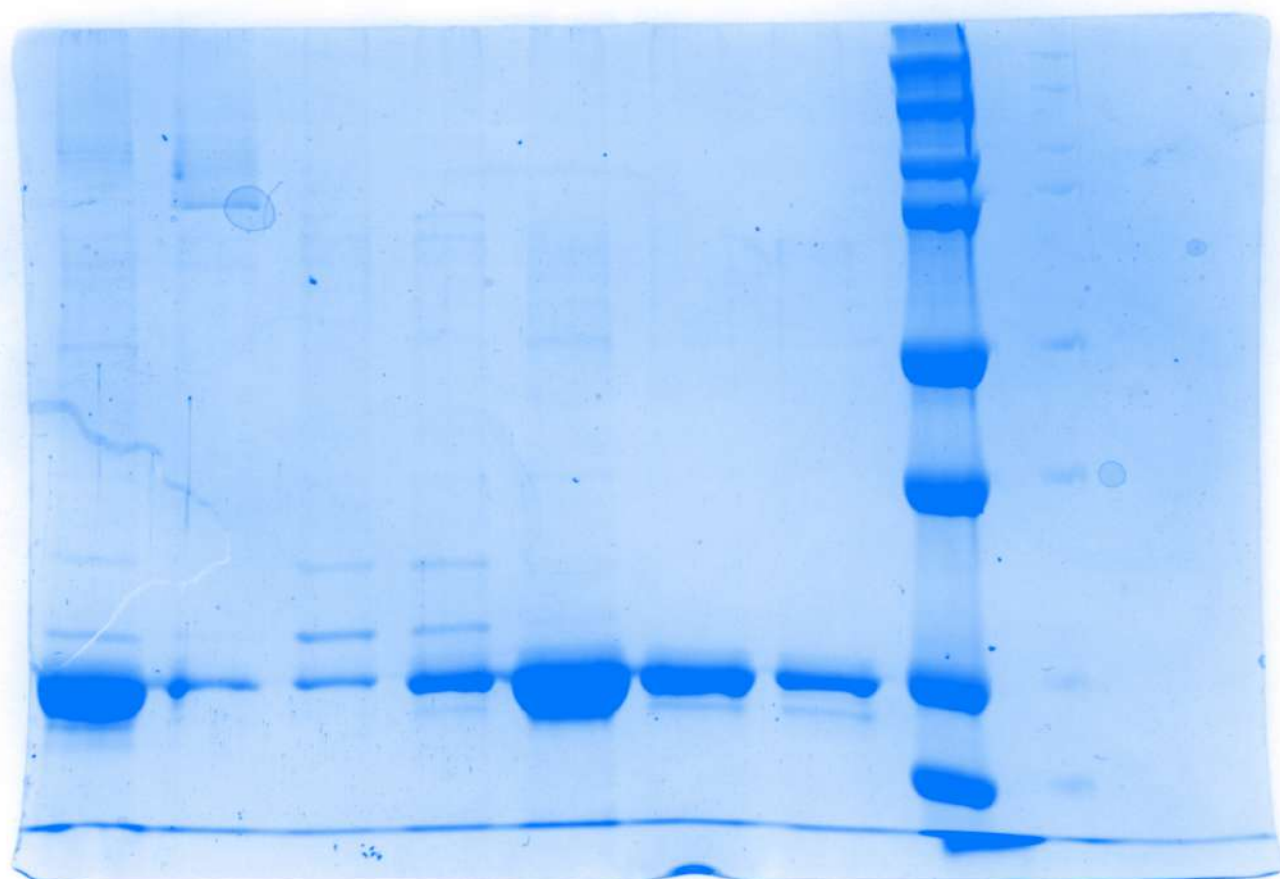

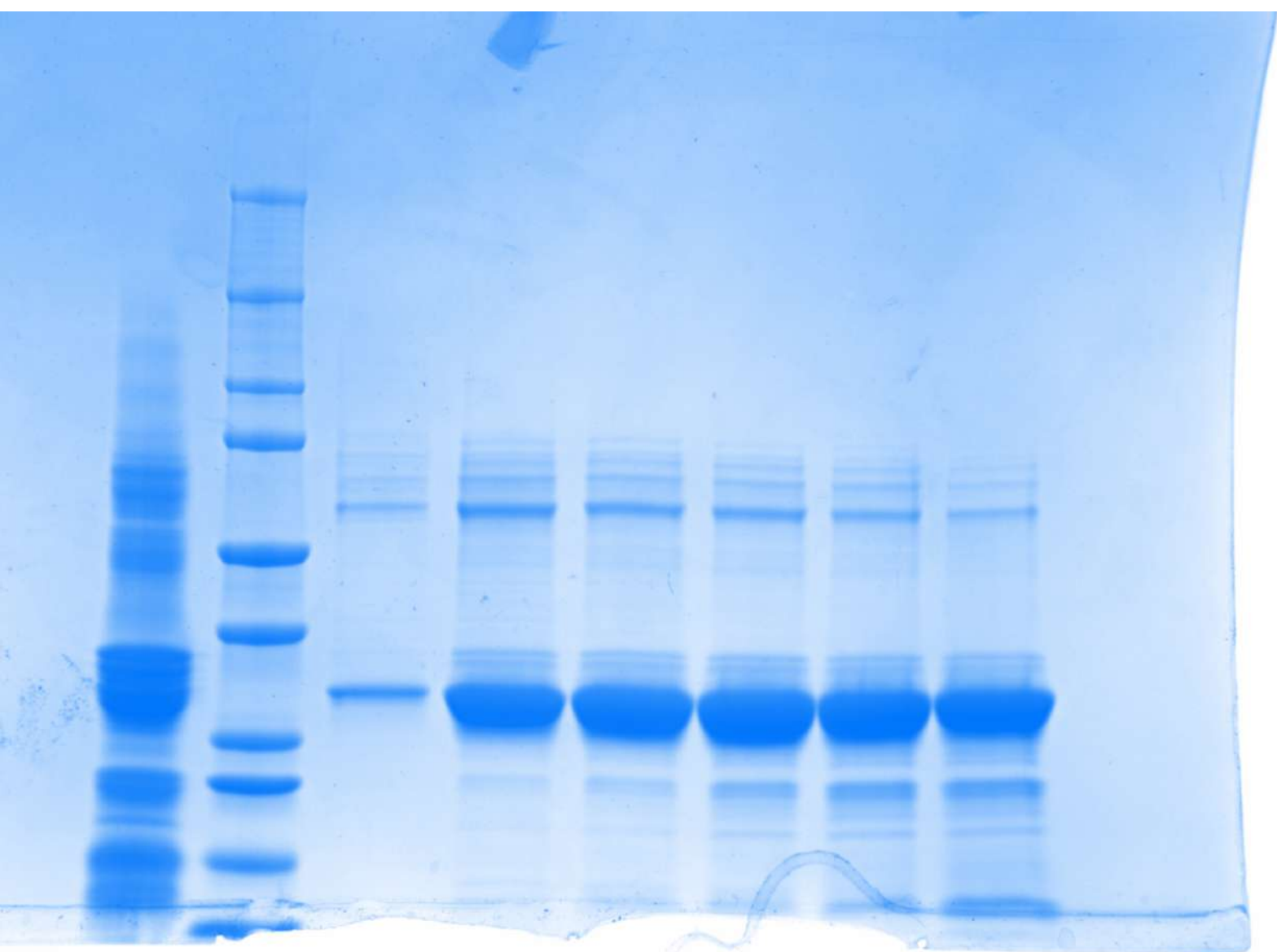

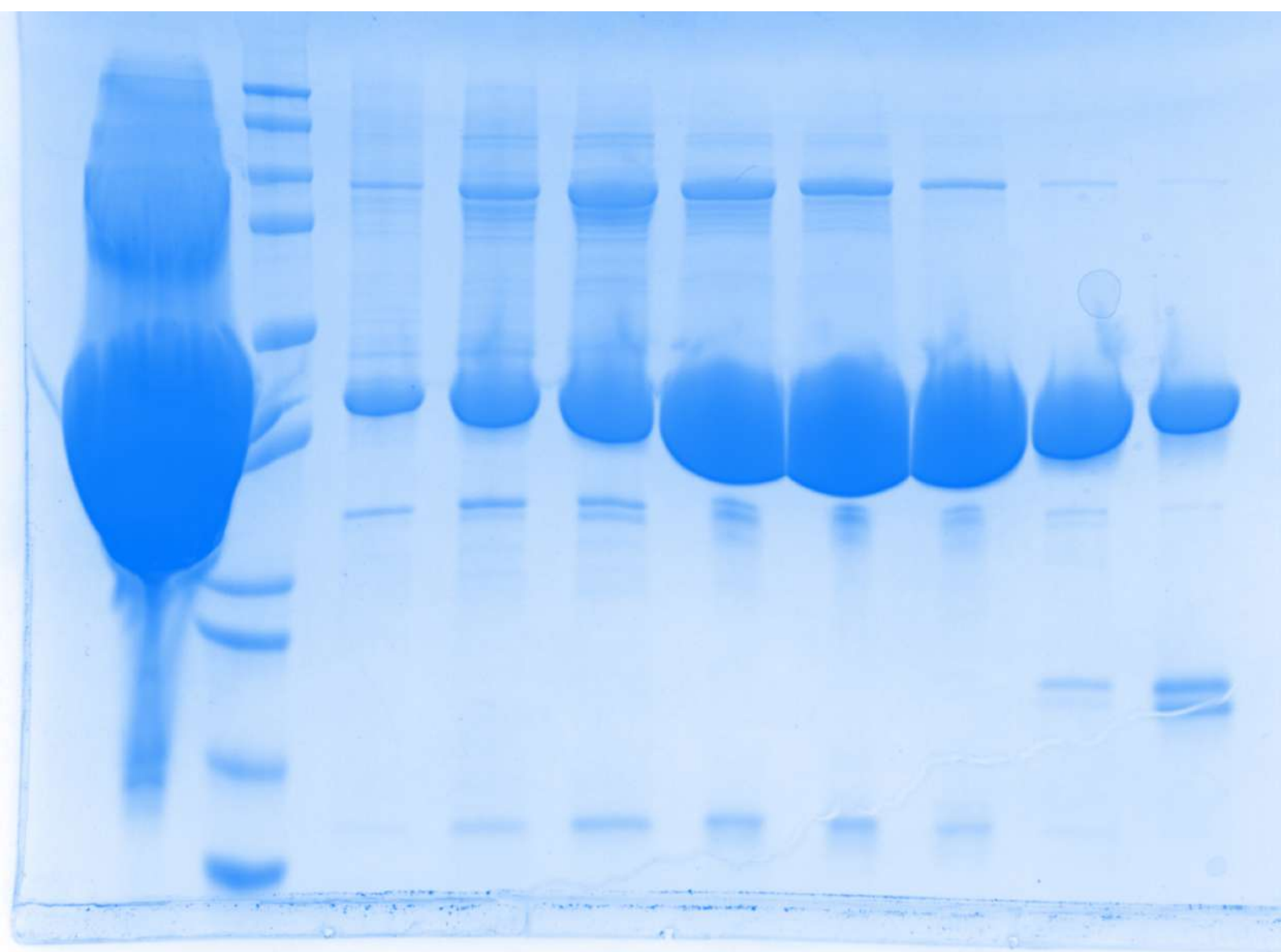

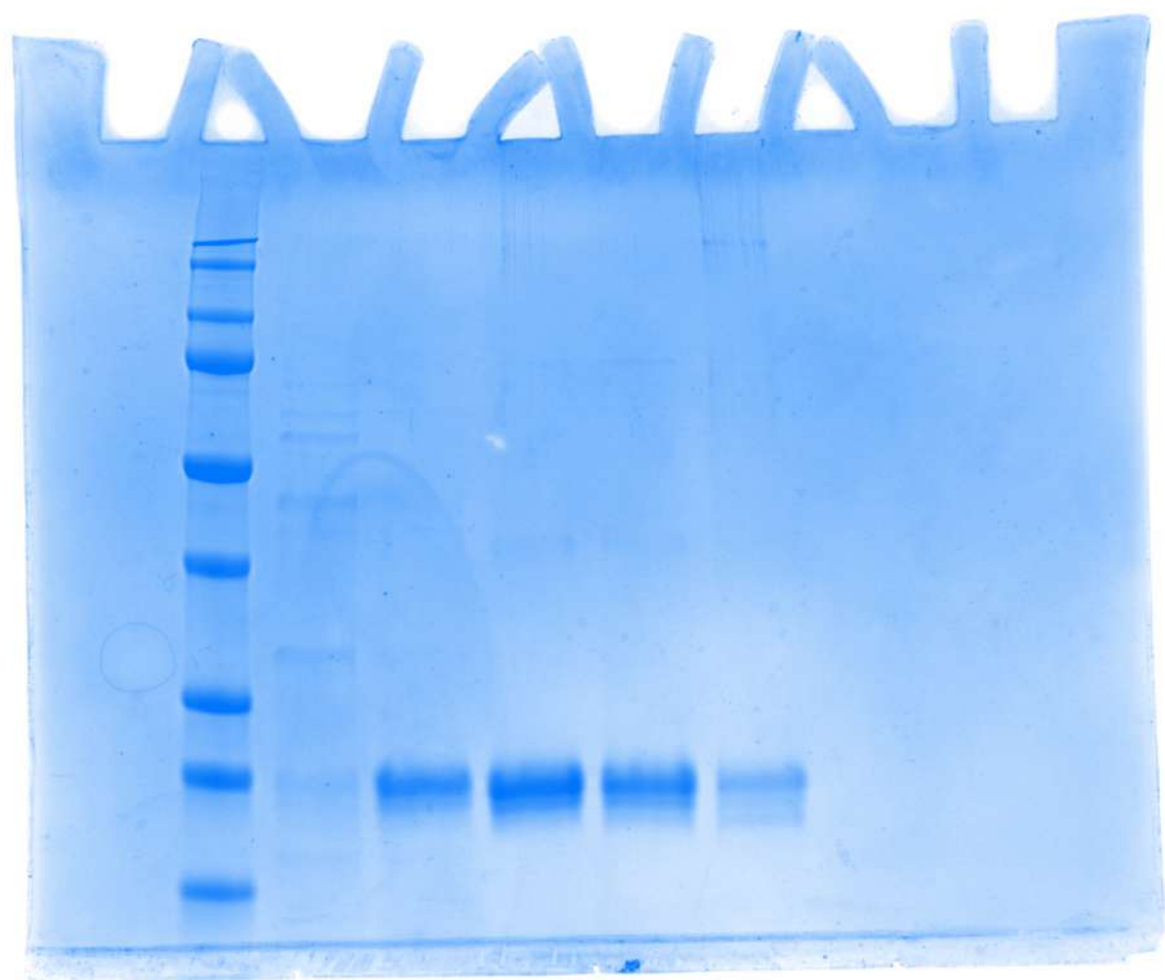

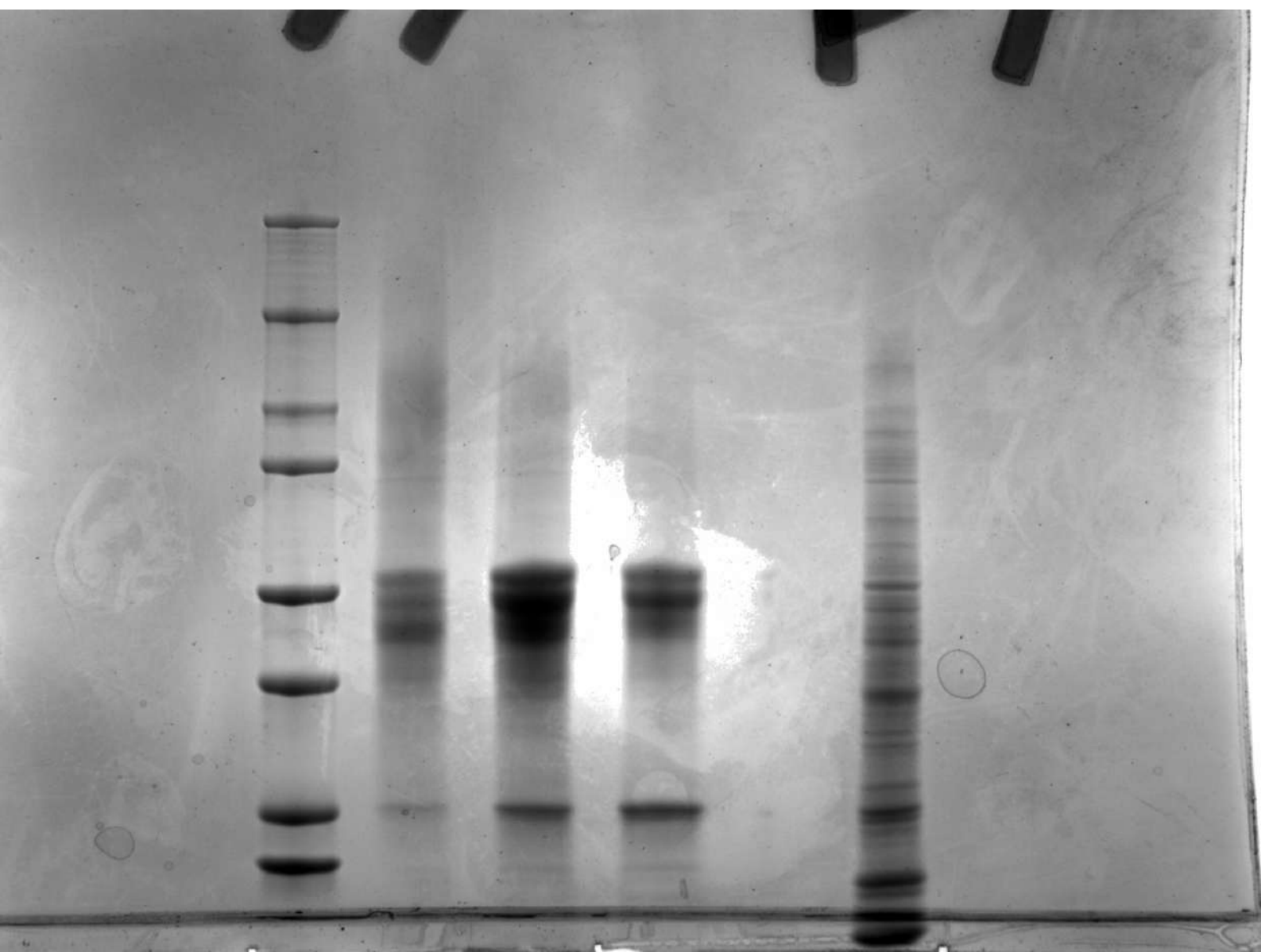

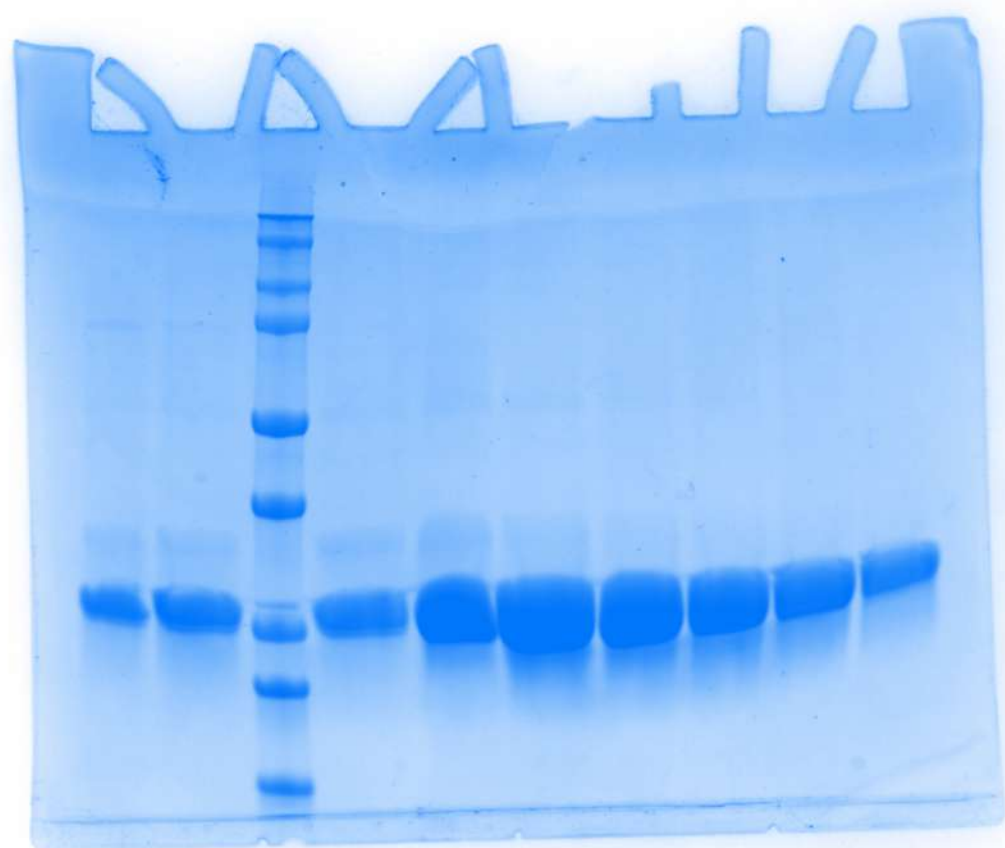

Supplement: Source Data [file EMS193170-supplement-Source_Data.zip › Source Data/41929_2023_1087_MOESM9_ESM.pdf]
