# Supplementary material for: In vitro construction of the COQ metabolon unveils the molecular determinants of coenzyme Q biosynthesis
Source: Nat Catal. Author manuscript; Available in PMC 2024 Feb 29. (PMC7615680; doi:10.1038/s41929-023-01087-z)
Supplement: Supplementary file [file EMS193170-supplement-Supplementary_file.pdf]

<u>**Table of contents**</u>

Supplementary Figures Pages 2-40

SF1. Supplementary Figure 1. Maximum likelihood phylogenies from Chordata phylum obtained for each COQ, FDXR and FDX2
SF2. Representative Multiple Sequence Alignments of each COQ
SF3: Limited proteolysis of COQ6 analysed by UHPL/HRMS and peptide mapping
SF4: Fluorescence NAD(P)H calibration line
SF5: UV/Vis spectra of **2** and **3**
SF6: COQ4 and COQ6 proposed mechanisms of action for C_1 modifications
SF7: Visible spectra of 4-AAY adducts
SF8: Blue Native PAGE analysis of the purified COQ metabolon
SF9: Chromatograms of experiments reported in Figure 6b
SF10: Chromatograms of experiments reported in Figure 6c
SF11: Calibration and peak integration of CoQ_1 and CoQ_1H_2
SF12: Monitoring inorganic phosphate production using Malachite green assay
SF13: Intact protein mass spec on COQ3
SF14: List of ancestral sequences and NCBI entries
SF15: NMR spectra of the CoQ compounds
SF16: SDS- and Blue Native-PAGE gels

Supplementary Tables Pages 41-42
ST1: List of primers for site-directed mutagenesis of COQ4
ST2: Peptide mapping analysis of the purified COQ metabolon

Supplementary Methods Pages 43-51
Construct design, cloning, mutagenesis and expression of the COQs, FDXR and FDX2……….43
Purification of GST-tagged FDXR, FDX2, COQ3, COQ4, COQ7, COQ8A and COQ8B……………44
Purification of HisSUMO tagged COQ6, COQ3, COQ4, COQ7 and COQ9…………………………..45
Purification of Strep tagged COQ5……………………………………………………………………………………47
UV/Visible Spectrometry………………………………………………………………………………………………48
4-aminoantipyrine spectrophotometric method of phenols/quinones analysis………………….48
Malachite Green ATPase assay……………………………………………………………………………………48
Limited proteolysis and peptide mapping……………………………………………………………………49
Intact protein mass spectrometry…………………………………………………………………………..50
Micro-Scale Thermophoresis……………………………………………………………………………………50
Nano Differential Scanning Fluorimetry……………………………………………………………………51

Supplementary References Pages 52

Supplementary Figures

COQ6

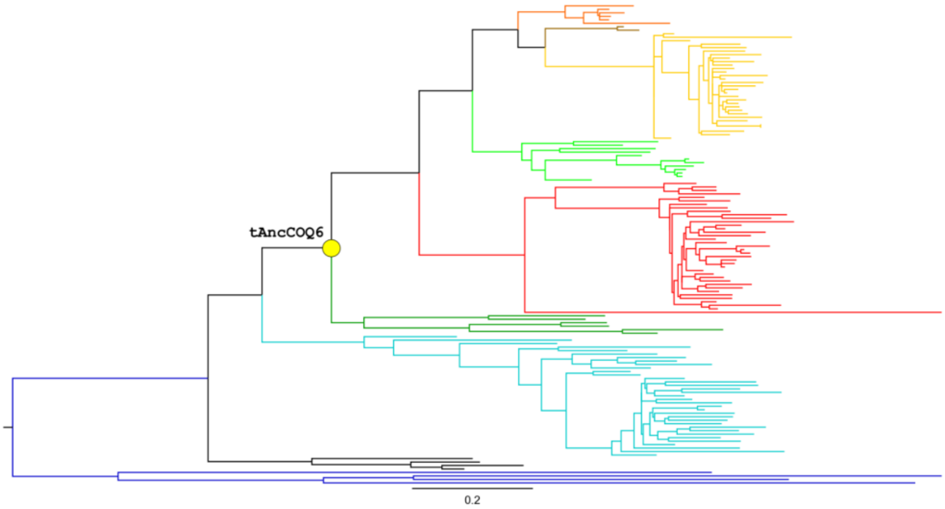

COQ3

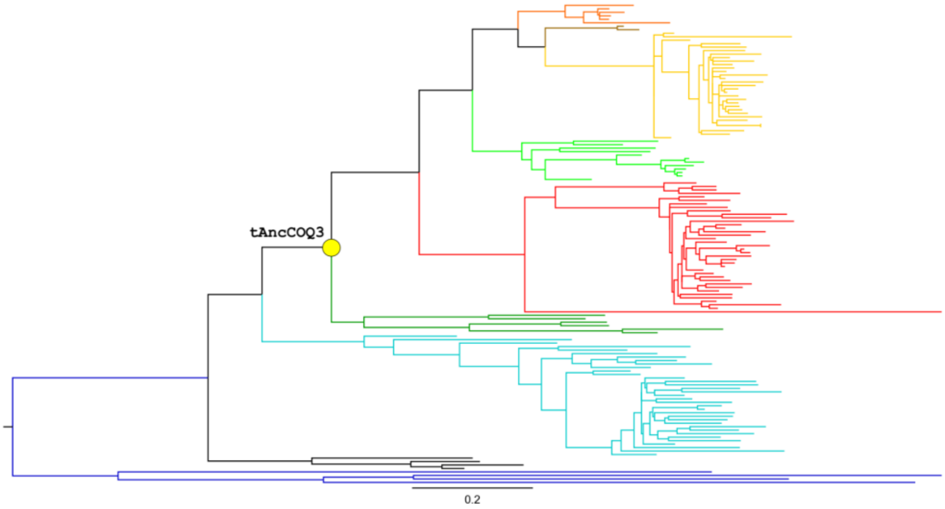

COQ4

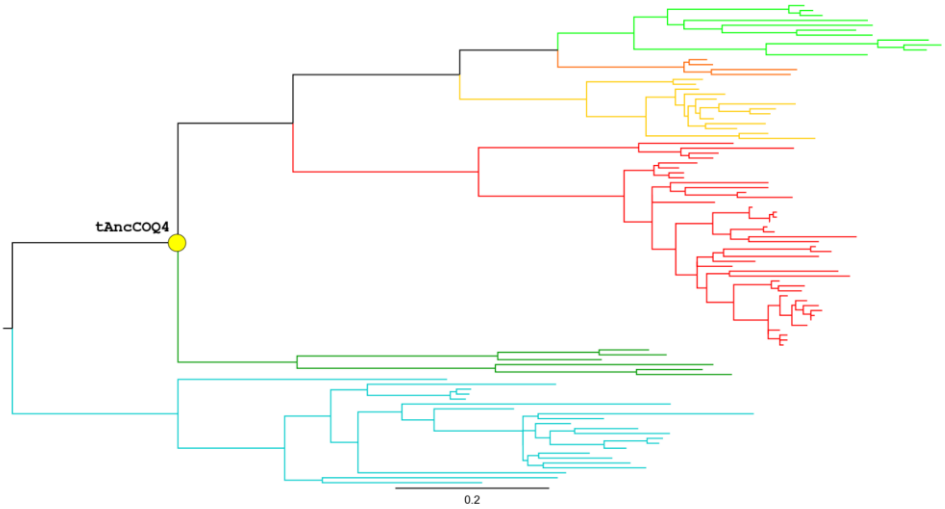

COQ5

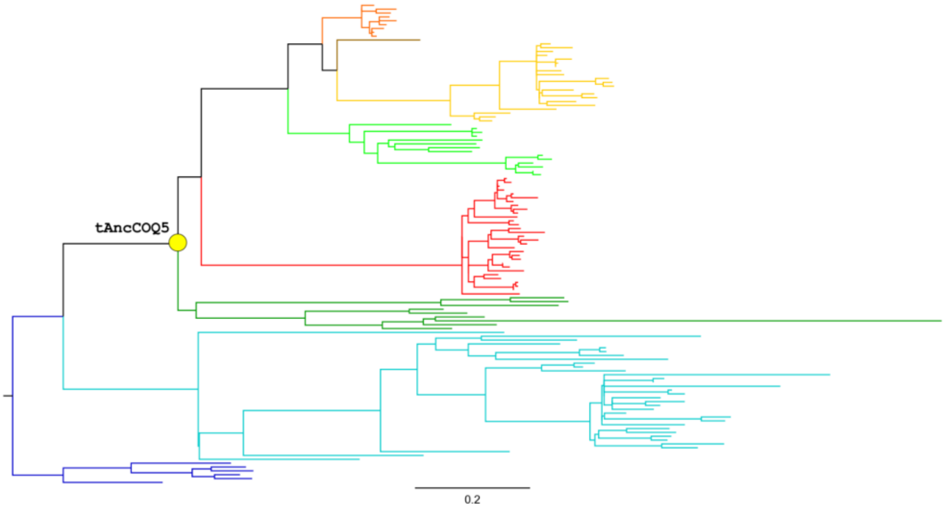

COQ7

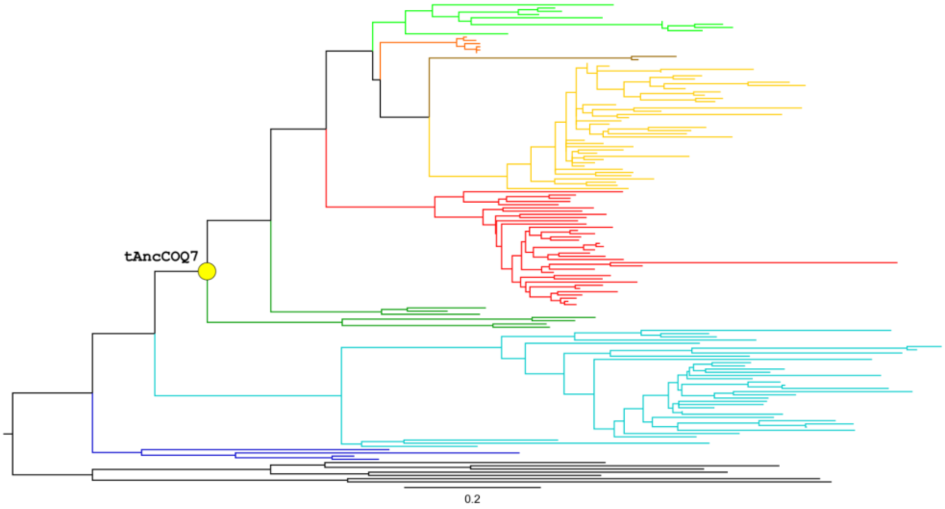

COQ8

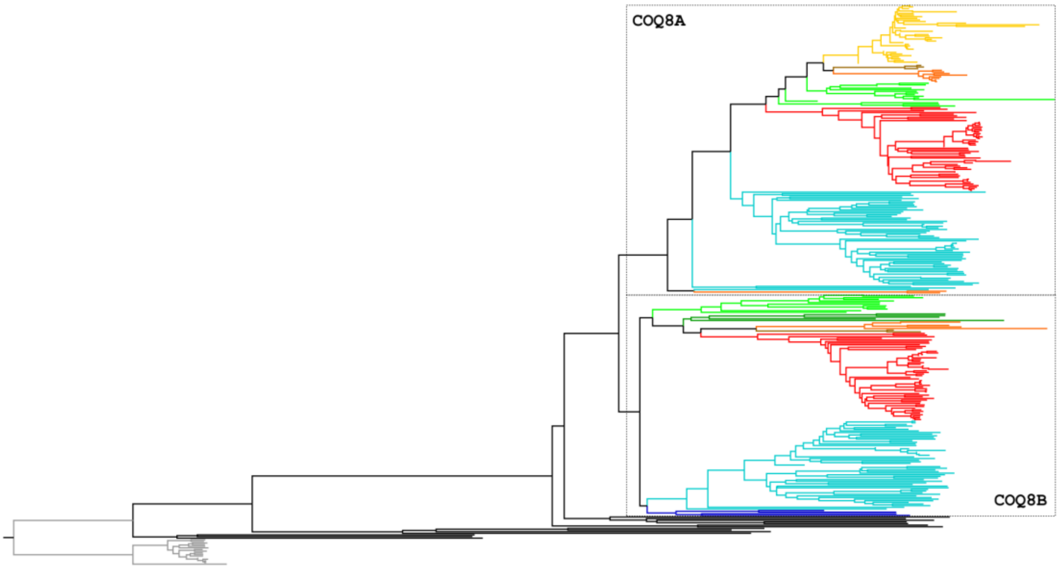

COQ8A

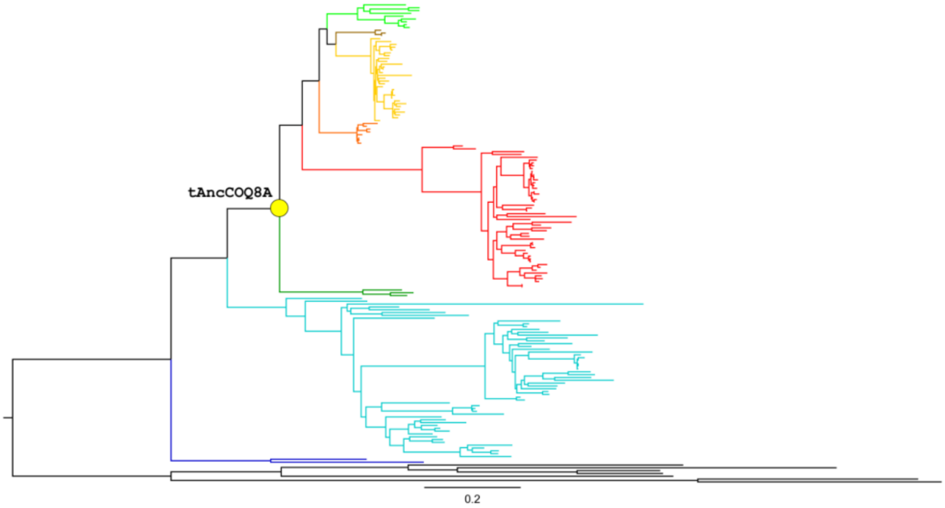

COQ8B

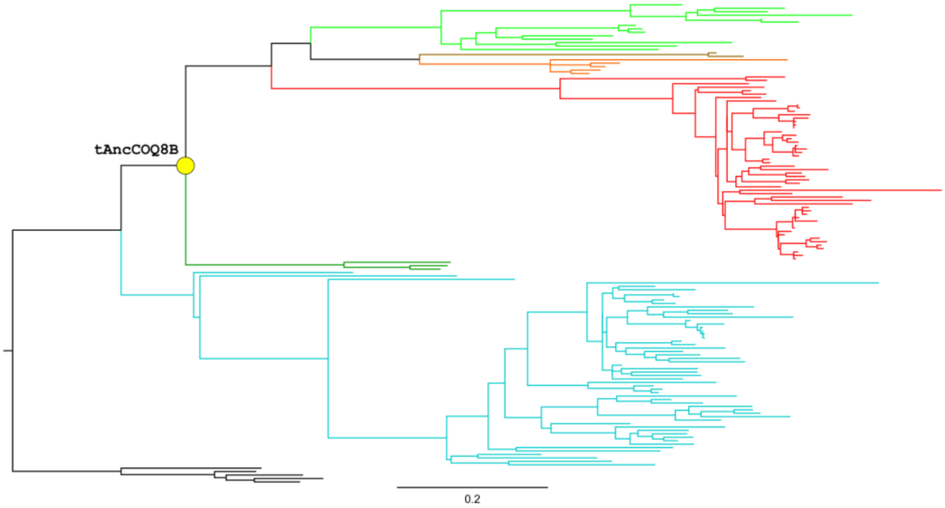

COQ9

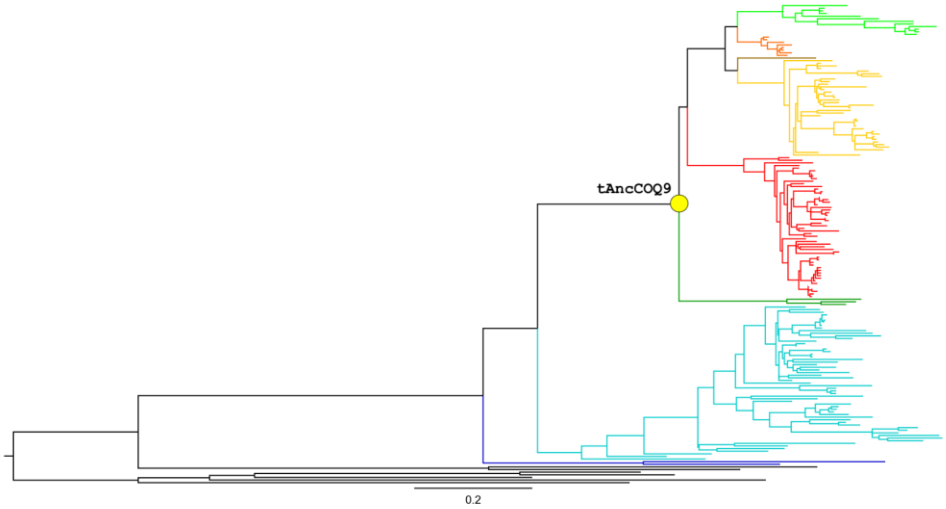

FDXR

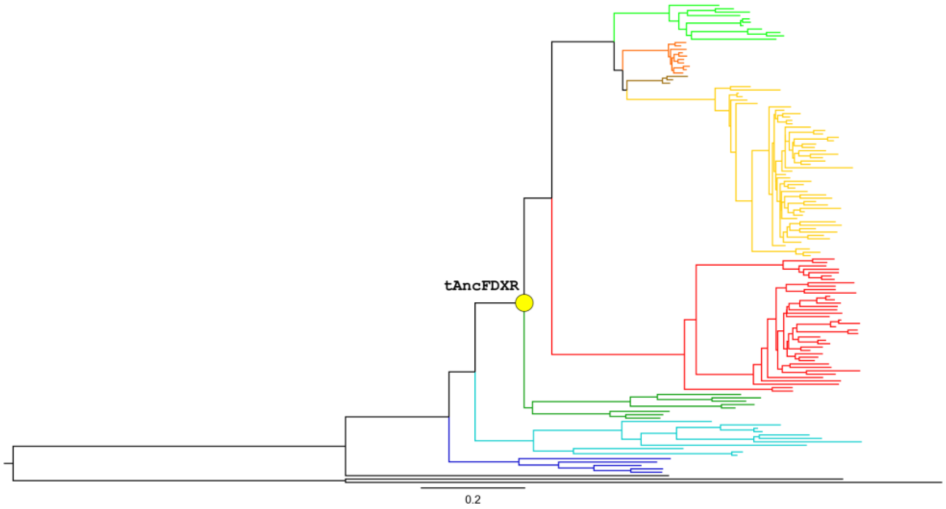

FDX2

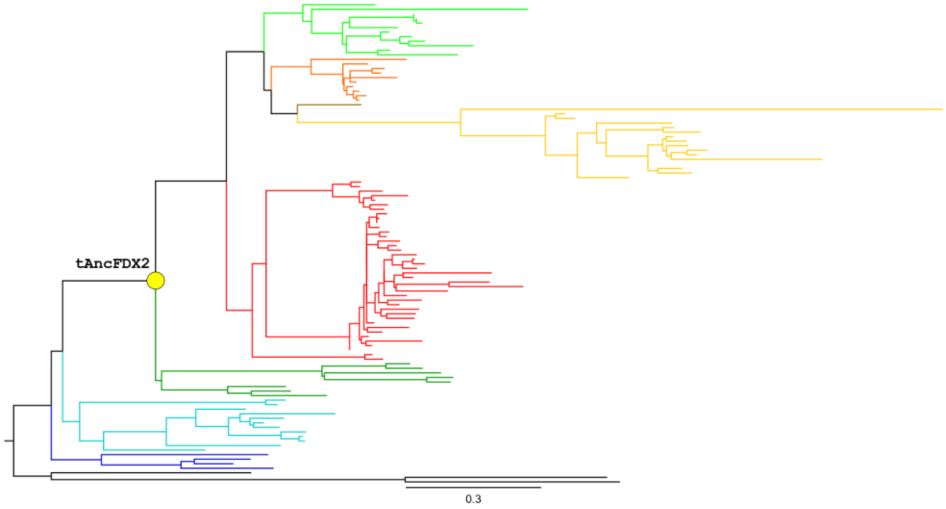

Supplementary Figure 1. Maximum likelihood phylogenies from Chordata phylum obtained for each COQ, FDXR and FDX2. Branches are colored according to the taxonomic groups: Mammalia (red), Aves (yellow), Testudines (orange), Crocodylia (brown), Lepidosauria (light green), Amphibia (dark green), Actinopterygii (cyan) and Chondrichthyes (blue). The root (black) is formed by sequences from hemichordata, echinodermata and cephalochordata groups. The resurrected tetrapod ancestors are marked with yellow circles. The scale bar indicates the substitutions *per* site. For COQ6 a multiple sequence alignment (MSA) including 138 sequences and 480 sites was employed, JTT was the best-fit substitution model and α= 0.81; for COQ3 a MSA including 181 sequences and 359 sites was employed, JTT was the best-fit substitution model and α= 0.92; for COQ4 a MSA including 98 sequences and 287 sites was employed, DUMMY2 was the best-fit substitution model and α= 48; for COQ5 a MSA including 125 sequences and 336 sites was employed, LG was the best-fit substitution model and α= 0.53; for COQ7 a MSA including 149 sequences and 217 sites was employed, JTT was the best-fit substitution model and α= 0.91; for COQ9 a MSA including 183 sequences and 323 sites was employed, JTT was the best-fit substitution model and α= 0.93. For COQ8, first a NJ tree (MSA with 346 sequences) is presented were the paralog duplication (COQ8A and COQ8B) is evidenced at the Chordata emergence (note that COQ8B paralog has been lost in Aves group). Sequences from the related family ACDK1 (aarF domain containing kinase 1) were included in the analysis for rooting purposes (shown in grey branches). For COQ8A a MSA including 170 sequences and 643 sites was employed, JTT was the best-fit substitution model and α= 0.84; for COQ8B a MSA including 140 sequences and 527 sites was employed, JTT was the best-fit substitution model and α= 0.87. For FDXR a multiple sequence alignment including 142 sequences and 500 sites was employed, JTT was the best-fit substitution model and α= 0.83. For FDX2 a multiple sequence alignment including 174 sequences and 180 sites was employed, JTT was the best-fit substitution model and α= 0.92.

COQ6

```
                             1         10        20                  30        40        50
tAncCOQ6                     MAA....RLGLSGWGRRRLRLRCGARLSLA.....RRGLARCCRRRSSSGGPAVYDVVIS
XP_029454311_Rhinatrema      MAALLAGPRGL.........CGFGL..........GAASCLARRSASSKPDLYDVVIS
NP_872282_Homo               MAA..............RLVSRCGAVRAAP.....HSGPLVSWRRWSGASTDTVYDVVVS
NWQ76339_Columbina           TSA..............AMAVVCGRGLLAPLAGRLRAGPSAPLR..SVAAAAPLYDVVVS

                                    60        70        80        90        100       110
tAncCOQ6                     GGGMVGTAMACALGYDPHFQDKKILLLEAGHKKVFDQLPESYSNRVSSITPGSATLLSSF
XP_029454311_Rhinatrema      GGGMVGTAMACALGYDPHFSNKRVLLLEAGHRKVIDQVADIYSNRVSAITPGSTTLLSSL
NP_872282_Homo               GGGLVGAAMACALGYDIHFHDKKILLLEAGPKKVLEKLSETYSNRVSSISPGSATLLSSF
NWQ76339_Columbina           GGGMVGSAMAAVLGHNIHFHDKKIALLEAGPRKEYDRMPESYSNRVSSISPGSATLLSSF

                                    120       130       140       150       160       170
tAncCOQ6                     GAWDHICNMRFKPFRRMQVWDACSDAMITFDKENLEDMGYIVENDVIMAALTKQLEAVSD
XP_029454311_Rhinatrema      GAWDHICSMRVKPFRRMQVWDGCSDAMITFDKEGLEDMGYIVENDVIVAALTRQLDALSK
NP_872282_Homo               GAWDHICNMRYRAFRRMQVWDACSEALIMFDKDNLDDMGYIVENDVIMHALTKQLEAVSD
NWQ76339_Columbina           GAWDHVCSLRLKAFRRMQVWDACSEAMIVFEKDDLDDMGYIVENDVIMSALTKQLDAVAD

                                    180       190       200       210       220       230
tAncCOQ6                     RVEVLYRSRAVGYTWPLPYHTAEANPWVQIELADGRRLQTKLLIGADGQNSMVRKAAGIQ
XP_029454311_Rhinatrema      RVEVFYKCRAVGYTWPLPYHTAEXNPWVQIELADGRNIQTKLLIGADGQNSTVRAATCIH
NP_872282_Homo               RVTVLYRSKAIRYTWPCPFPMADSSPWVHITLGDGSTFQTKLLIGADGHNSGVRQAVGIQ
NWQ76339_Columbina           RVQVFYGSRAVGYTWPLPTHNCDTSPWVQIELADGRRLQTKLLIGADGHNSVVRKEAEIK

                                    240       250       260       270       280       290
tAncCOQ6                     NVQWNYDQSAVVATLHLSEATDNNVAWQRFLPTGPIALLPLSDTVSSLVWSTSHEHAAEL
XP_029454311_Rhinatrema      SLKWSYDQVAVTATLHLSEATENNVAWQRFLPSGPIALLPLSDTCSSLVWSTSSEHAAEL
NP_872282_Homo               NVSWNYDQSAVVATLHLSEATENNVAWQRFLPSGPIALLPLSDTLSSLVWSTSHEHAAEL
NWQ76339_Columbina           NIEHRYDQSAVVATLHLSEATDNNVAWQRFLPTGPIALLPLSDTASSLVWSTSHEHASEL

                                    300       310       320       330       340       350
tAncCOQ6                     LSMDEESFVDAINSAFWSNENHSEFIDTAGSMFRSALSLLMPSGTSARQLPPSVARVDPK
XP_029454311_Rhinatrema      LSMDDERFVDTINSAFWSNETHSEFIHSAGSLFQSATSLLMPSGFSARQLPPSVARIDAK
NP_872282_Homo               VSMDEEKFVDAVNSAFWSDADHTDFIDTAGAMLQYAVSLLKPTKVSARQLPPSVARVDAK
NWQ76339_Columbina           LAMDEESFVDSINSAFWSNVNHTDFIDTAGAVFRSAISLLKPSGTAVRQLPPSVAKVDPE

                                    360       370       380       390       400       410
tAncCOQ6                     SRAMFPLGLGHATEYVRHRVALIGDAAHRVHPLAGQGVNMGFGDVACLTHHLSQAAFNGK
XP_029454311_Rhinatrema      SRAMFPLGMGHATEYIRHRVALIGDAAHRVHPLAGQGVNMGFGDVASLARHLSRAAFNGK
NP_872282_Homo               SRVLFPLGLGHAAEYVRPRVALIGDAAHRVHPLAGQGVNMGFGDISSLAHHLSTAAFNGK
NWQ76339_Columbina           SRAMFPLGMGHATEYVHHRVALIGDAAHRVHPLAGQGVNLGFGDIACLAHHLSAAAFNGS

                                    420       430       440       450       460       470
tAncCOQ6                     DLGSTRHLLEYETERQRHNLPLMAAIDLLKRLYSTKVAPFVLLRTLGLQATNALSPVKEQ
XP_029454311_Rhinatrema      DLGSIRHLLAYETERQRHNVLLMSAIDMLNRLYSTKEAPLVLLRTLGLQATNMLSPVKEQ
NP_872282_Homo               DLGSVSHLTGYETERQRHNTALLAATDLLKRLYSTSASPLVLLRTWGLQATNAVSPLKEQ
NWQ76339_Columbina           DLGSLKHLLKFETERQRHNVSLIAAIDVLKRLYSTKLAPLVLLRTWGLQATNALPPLKVR

tAncCOQ6                     IMAFASK
XP_029454311_Rhinatrema      IMNFASK
NP_872282_Homo               IMAFASK
NWQ76339_Columbina           MSLQWGP
```

COQ3

```
                             1        10        20        30        40        50
tAncCOQ3                     MWGGGR.GSRAGRLLVALRGRSRGR.GAGCRRLSLAAAAGNHYGWTLQMAPRFKSS....
NWQ76719_Columbina           ............................................................
XP_029451244_Rhinatrema      M..............AAWLSRSGGRYSPQCRRLTVVAAGE.......RRRRRWRGADGDE
NP_059117_Homo               MWSGRKLGSSGGWFLRVLGPGGCNT.KAARPLISSAVYVKNQLSGTLQIKPGVFNE....

                                   60        70        80        90       100       110
tAncCOQ3                     .NRTMWLKSNSTTFASLTKMKSSRSAVKRMYSTSQTTVDPKEMKKFQALAHKWWDEQGEY
NWQ76719_Columbina           ..................QLSLFRLTTKRLFSTSHSSVDSEELKKFQLLAHKWWDEEGEY
XP_029451244_Rhinatrema      MNPGMLLTSASRTFSRLSALSS.RCTMKWMHTAAQ.TVDSREMQKFQMHAYKWWDEEGVY
NP_059117_Homo               .YRTIWFKSYRTIFSCLNRIKSFRYPWARLYSTSQTTVDSGEVKTFLALAHKWWDEQGVY

                                  120       130       140       150       160       170
tAncCOQ3                     AALHSMNDLRVPFIRDTLLNMSGDHQLGSPLSGMKILDVGCGGGLLSEPLGRLGASVTGI
NWQ76719_Columbina           SALHSMNDIRVPFIRDTLLNMSSNYHLGNPLSGVKILDVGCGGGLLSEPLGRLGASVTGI
XP_029451244_Rhinatrema      AALHTMNDLRVPFIRDTLINRKRDHDPGRPLAGVTLLDVGCGGGLLSEPLGILGAAVTGI
NP_059117_Homo               APLHSMNDLRVPFIRDNLLKTIPNHQPGKPLLGMKILDVGCGGGLLTEPLGRLGASVIGI

                                  180       190       200       210       220       230
tAncCOQ3                     DPLEDNIRTAELHKSFDPVLDKRIQYKACSLEEIVEEATETFDAVVASEVVEHVADVETF
NWQ76719_Columbina           DPLEDNIRTADQHKSFDPVLAKRIQYKSSSLEEIVEECMETFDVIVASEVVEHVADLEMF
XP_029451244_Rhinatrema      DPLKENIRTAELHKSFDPVLAKQIQYKTSSLEEIVAESTEAFDAVVASEVVEHVGDVESF
NP_059117_Homo               DPVDENIKTAQCHKSFDPVLDKRIEYRVCSLEEIVEETAETFDAVVASEVVEHVIDLETF

                                  240       250       260       270       280       290
tAncCOQ3                     IKCCYQVLKPGGSLFITTINKTQLSYALGIVVAERIMGIVPAGTHDWEKFISPEELERLL
NWQ76719_Columbina           IKCCSQVLKPEGSLFITTINKTQLSYILGIVVAEKIIGVVPEGTHEWEKFVPPEELERLL
XP_029451244_Rhinatrema      IKCCHQVLKPGGSLFITTISKTMMSYALGIVVAERIMGIVPAGIHDWEKFVLPEELERLL
NP_059117_Homo               LQCCCQVLKPGGSLFITTINKTQLSYALGIVFSEQIASIVPKGTHTWEKFVSPETLESIL

                                  300       310       320       330       340       350
tAncCOQ3                     ESNGFSVETVNGMLYNPLSGSWSWIENTSINYALHAVKSKVQEQSDSTEPPSEQEQEQHQ
NWQ76719_Columbina           ESNGFSVKTVKGMLYNPLSGSWSWMESTSINYALHAVKSGAQGQPSPTDALSELENDQPS
XP_029451244_Rhinatrema      ESNGFVVETLSGMLYNPFCRSWSWMENTSLNYAMHAVKTIQPEEPASTEPAAERKQEQAE
NP_059117_Homo               ESNGLSVQTVVGMLYNPFSGYWHWSENTSLNYAAYAVKSRVQEHPASAEFVLKGETEELQ

                                  360
tAncCOQ3                     AETSTSTTV......
NWQ76719_Columbina           ATAGTAV........
XP_029451244_Rhinatrema      DSTSSAL........
NP_059117_Homo               ANACTNPAVHEKLKK
```

COQ4

```
tAncCOQ4                 MA...TLLRRARGLLPLLRPVLGSP......GLTGRALCMR...QAPAA...........
Q9Y3A0_Homo              MA...TLLR......PVLRRLCGLP......GL...........QRPAA...........
NXE46430_Casuarius       HP..GAGGSRSPG..........PR.....ALSAGAVRPRHAGRRAAA...........
XP_029467832_Rhinatrema  MAMVTALLREVRGCSLLLRSLGRVPRQSVIAAATGKGQRWY...QTNKASYYDMHEDAFT

tAncCOQ4                 ....DSPLRAAEEGYGPLYPGHIPTSPLQKALLAAGSACMALYNPYRHDMVAVLGETTGH
Q9Y3A0_Homo              ....EMPLRARSDGAGPLYSHHLPTSPLQKGLLAAGSAAMALYNPYRHDMVAVLGETTGH
NXE46430_Casuarius       ....EDGAAEEQEGCCQLYPGHIPTSPLQKALLAAGSAAMALYDPYRHDMVAVLGETTGC
XP_029467832_Rhinatrema  HMEEDNTKEAAGEKYHPLYPNHIPTNAFQKALLTVGSAMMSLYDPYRHDMIAVLGETTGP

tAncCOQ4                 LALQNLRDRMRNDPEGYQILQERPRIRLSTLDLAKLRSLPDGSFGREYVRFLDDNRVSPD
Q9Y3A0_Homo              RTLKVLRDQMRRDPEGAQILQERPRISTSTLDLGKLQSLPEGSLGREYLRFLDVNRVSPD
NXE46430_Casuarius       LVLPNLRDKMKHDPEGYRILRERPRIRLSTLDVNRLRGLPDGTLGREYTRFLEDNKVSPD
XP_029467832_Rhinatrema  LVLQNLRDRMRNDPEGNQILQERPRIQMSTLEMHRLRELPDATFGREYIRFLDVNKVSPD

tAncCOQ4                 TRAPVKFVDDEELAYVIQRYREVHDLLHTLLGMPTNMLGEVVVKWFEAVQTGLPMCILGA
Q9Y3A0_Homo              TRAPTRFVDDEELAYVIQRYREVHDMLHTLLGMPTNILGEIVVKWFEAVQTGLPMCILGA
NXE46430_Casuarius       TRMPAKFVDDEELAYVIQRYREVHDLMHTLLGMPTNMLGEVVVKWFEAIQTGLPMCVLGA
XP_029467832_Rhinatrema  TRMPVKFVDNEELAYVIQRYREVHDLMHTLLGMPTNMLGEVVVKWFEAIQTGLPMCILGA

tAncCOQ4                 AFGPIRLSARKLQVLVTELIPWAVQNGRNARCVLNIYYERRWEQSLESLREELGITPPPI
Q9Y3A0_Homo              FFGPIRLGAQSLQVLVSELIPWAVQNGRRAPCVLNLYYERRWEQSLRALREELGITAPPM
NXE46430_Casuarius       AFGPVRLNARKLRVLTTELIPWAIQSGRNANCILNIYYERRWEQTVESLREEIGIFSPP.
XP_029467832_Rhinatrema  TFGPLRLNAKRLQVLMMELLPWVIQCGRNSQFVMNVYYEKRWEQTMESLREELGITPPPI

tAncCOQ4                 RVTGLA
Q9Y3A0_Homo              HVQGLA
NXE46430_Casuarius       ......
XP_029467832_Rhinatrema  IK...V
```

COQ5

```
tAncCOQ5                 ..MAASMRCA....C....SRALCGCR........SGARVCCRAHSTEAAEKETHFGFQT
NWQ77319_Columbina       ...................RGL...........................AAGPEMHFGFQT
Q5HYK3_Homo              ..MAAPGSCALWSYCGRGWSRAMRGCQLLGLRSSWPGDLLSARLLSQEKRAAETHFGFET
XP_029474956_Rhinatrema  MAVLAGGRC.LGGLC....SRLLGS..........PGKLWFLRRQHTDGQYKKASFGFQS

tAncCOQ5                 VSEEEKGEKVYQVFENVAKKYDIMNDAMSLGIHRLWKDALLHQMNPYPGTQLLDVAGGTG
NWQ77319_Columbina       VTEAERREKIYQVFESVAKKYDVMNDSMTLGIHRVWKDILVHKMNPSPGTLLLDVAGGTG
Q5HYK3_Homo              VSEEEKGGKVYQVFESVAKKYDVMNDMMSLGIHRVWKDLLLWKMHPLPGTQLLDVAGGTG
XP_029474956_Rhinatrema  VTEAEKNEKVHELFDRVSLKYDLMNDVMTLGIHRLWKDSLLRLMNPYPGTQLLDVAGGTG

tAncCOQ5                 DIAFRFINYVRSQRERQVRQELKSHQNLSWQEISKSYQEEEQDSLGGSQAVICDINKEML
NWQ77319_Columbina       DIAFRFINYVRSVRERQLQRKLRHHQNLSWQEIAESYQEDKSKSLGGSQVVVCDINKEML
Q5HYK3_Homo              DIAFRFLNYVQSQHQRKQKRQLRAQQNLSWEEIAKEYQNEE.DSLGGSRVVVCDINKEML
XP_029474956_Rhinatrema  DIAFLFLDYIHSQREVQLRRDLKSYQNLSWLEVSKSYQKTRQDLLGGSHVVICDVNQKMM

tAncCOQ5                 KVGKQKAQQLGYSEGLSWVVGNAEELPFDDDKFDVYTIAFGIRNVTHIDQALQEAYRVLK
NWQ77319_Columbina       KVGKEKAQNLGYTEGLSWVLGNAEELPFDDDKFDVYTIAFGIRNVTRIDLALQEAYRVLK
Q5HYK3_Homo              KVGKQKALAQGYRAGLAWVLGDAEELPFDDDKFDIYTIAFGIRNVTHIDQALQEAHRVLK
XP_029474956_Rhinatrema  AIGEKKAQHLGYTEGLSWVVGNAEELPFTADKFDVYSVAFGIRNMTHIKQALQEAYRVLK

tAncCOQ5                 PGGRFLCLEFSQVNNPLISRLYDLYSFQVIPVLGEVIAGDWKSYQYLVESIRRFPSQEEF
NWQ77319_Columbina       PGGRFLCLEFSHVSNPLLSSLYDLYSFQVIPVLGEVIAGDWKSYQYLVESIRRFPPQEEL
Q5HYK3_Homo              PGGRFLCLEFSQVNNPLISRLYDLYSFQVIPVLGEVIAGDWKSYQYLVESIRRFPSQEEF
XP_029474956_Rhinatrema  PGGRFLCLEFSHASNPLISRIYDLYSFQVIPVLGEVIAGEWQSYQYLVESIRLFPTQEEF

tAncCOQ5                 KAMIEDAGFFKVEYHNLTSGIVAIHSGFKL
NWQ77319_Columbina       KAMIEDAGFLKVDYQNLNLGIVAIHSGFKL
Q5HYK3_Homo              KDMIEDAGFHKVTYESLTSGIVAIHSGFKL
XP_029474956_Rhinatrema  KAMIEDAGFMKVDYHNLTSGVVAIHSGFKL
```

COQ7

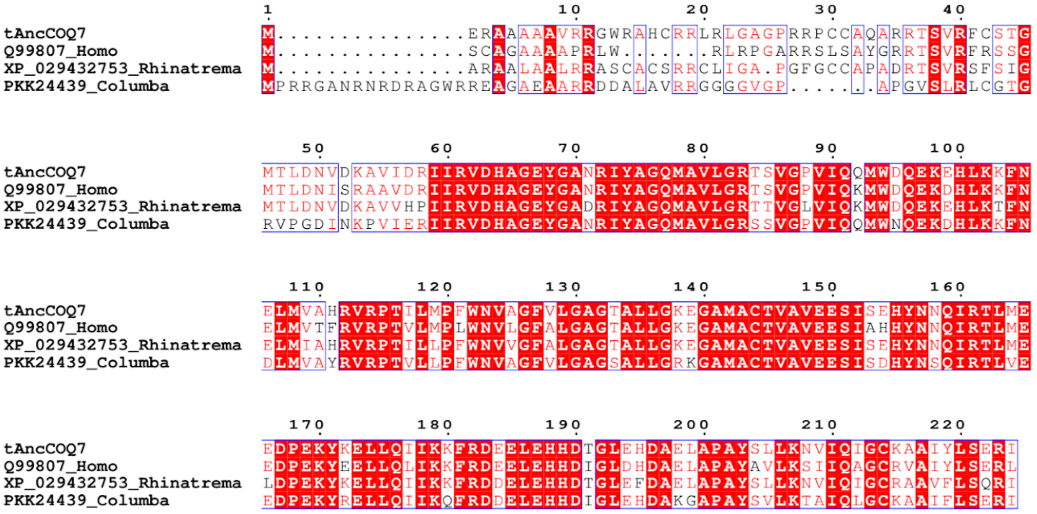
tAncCOQ7
Q99807_Homo
XP_029432753_Rhinatrema
PKK24439_Columba

COQ9

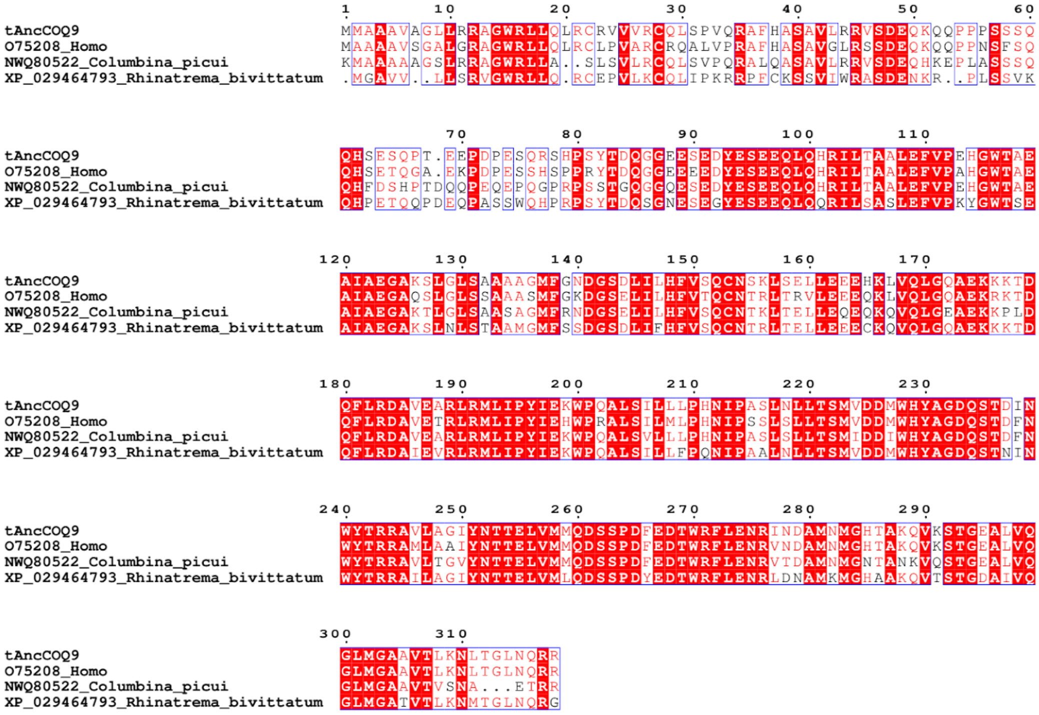
tAncCOQ9
O75208_Homo
NWQ80522_Columbina_picui
XP_029464793_Rhinatrema_bivittatum

COQ8A

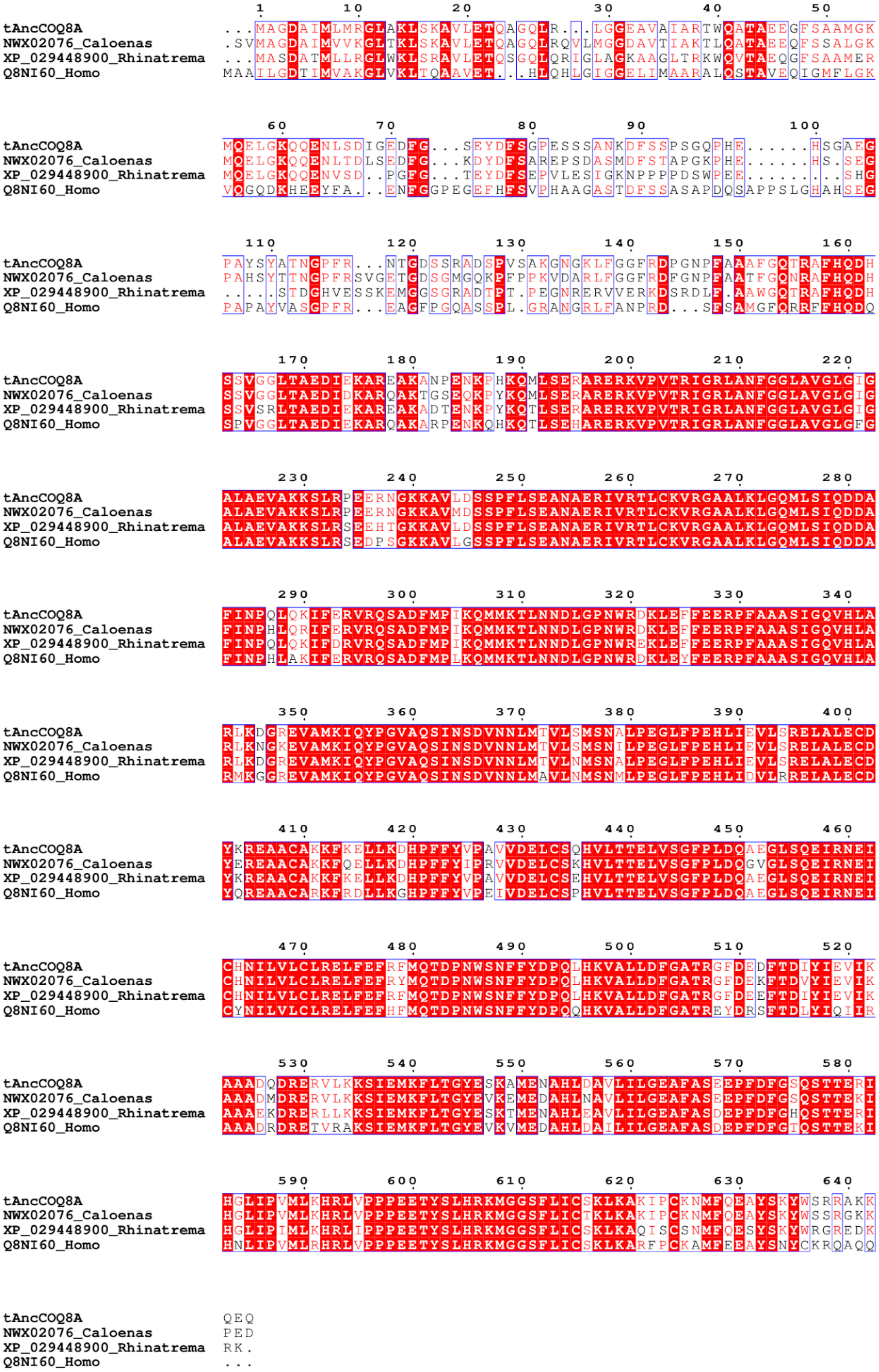
tAncCOQ8A
NWX02076_Caloenas
XP_029448900_Rhinatrema
Q8NI60_Homo

COQ8B

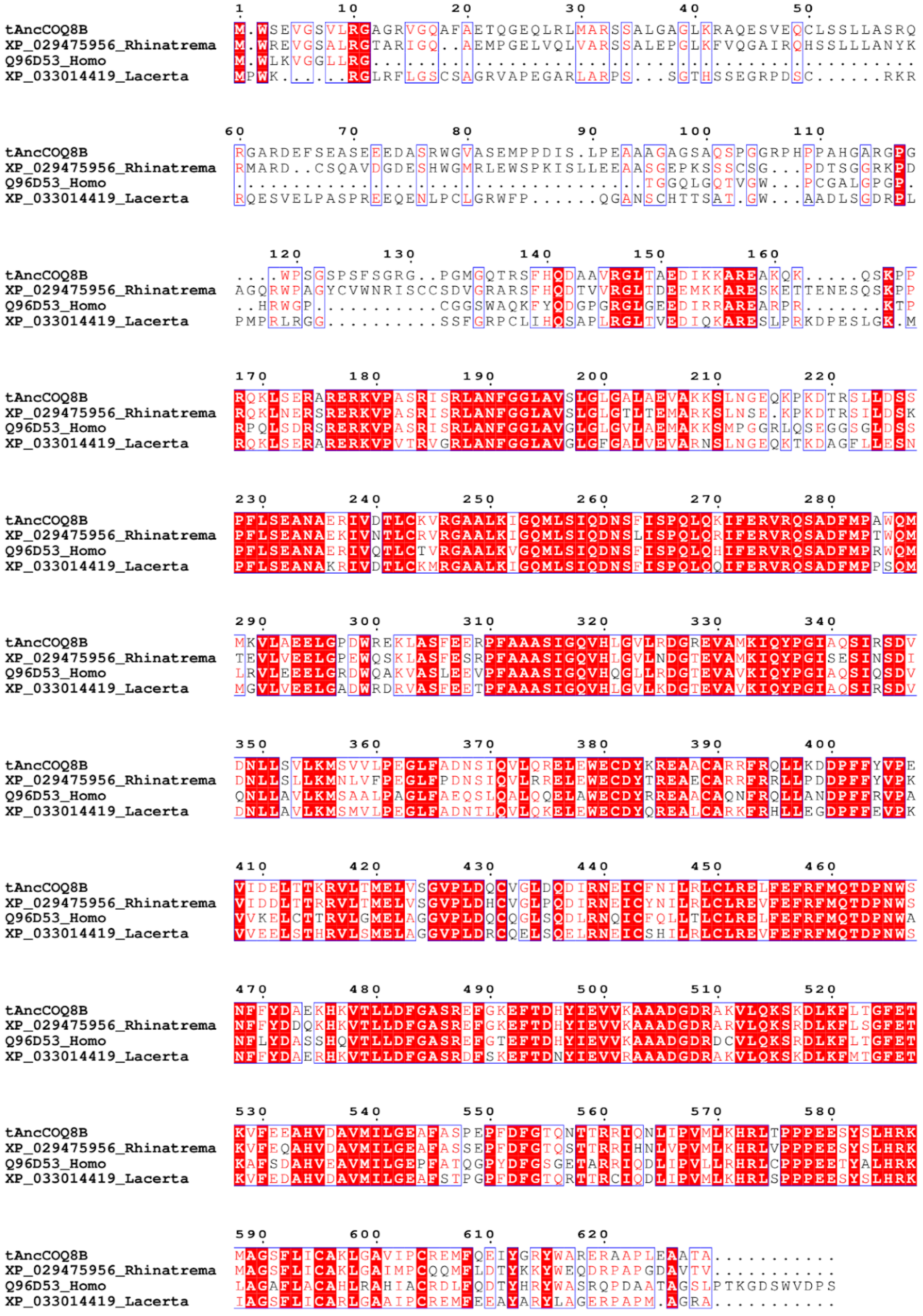
tAncCOQ8B
XP_029475956_Rhinatrema
Q96D53_Homo
XP_033014419_Lacerta

FDXR

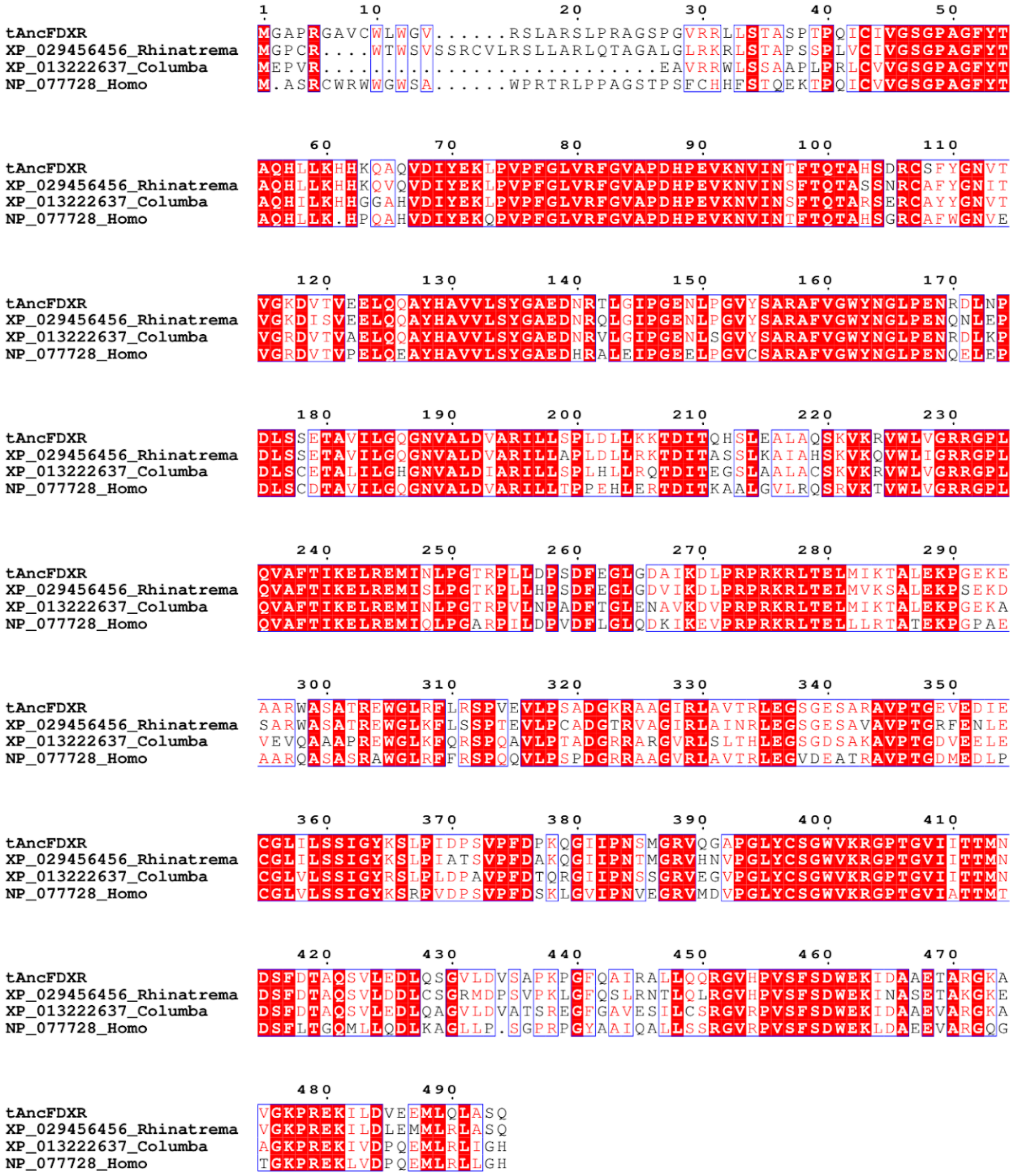
tAncFDXR
XP_029456456_Rhinatrema
XP_013222637_Columba
NP_077728_Homo

FDX2

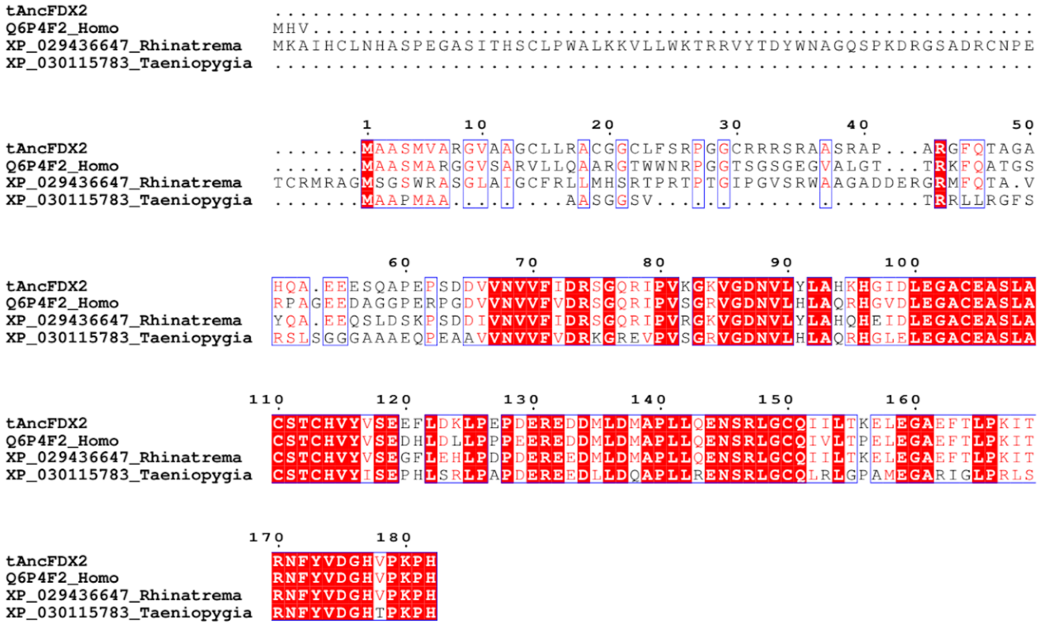

Supplementary Figure 2. Representative Multiple Sequence Alignments of each COQ. For each COQ a multiple-sequence alignment was constructed including the ancestral sequence reconstructed (full-length) from the tetrapoda superclass plus representative extant sequences from each of the three classes: amphibia (*Rhinatrema bivittatum* in all cases), reptilia (*Columba livia*, *Columbina picui*, *Casuarius casuarius*, *Caloenas nicobarica*, *Lacerta agilis* or *Taeniopygia guttata*) and mammalia (*Homo sapiens* in all cases). For each selected sequence the GenBank accession code is given in the name. The multiple-sequence alignments are colored according to the percentage of equivalence among residues calculated from their physical-chemical properties.

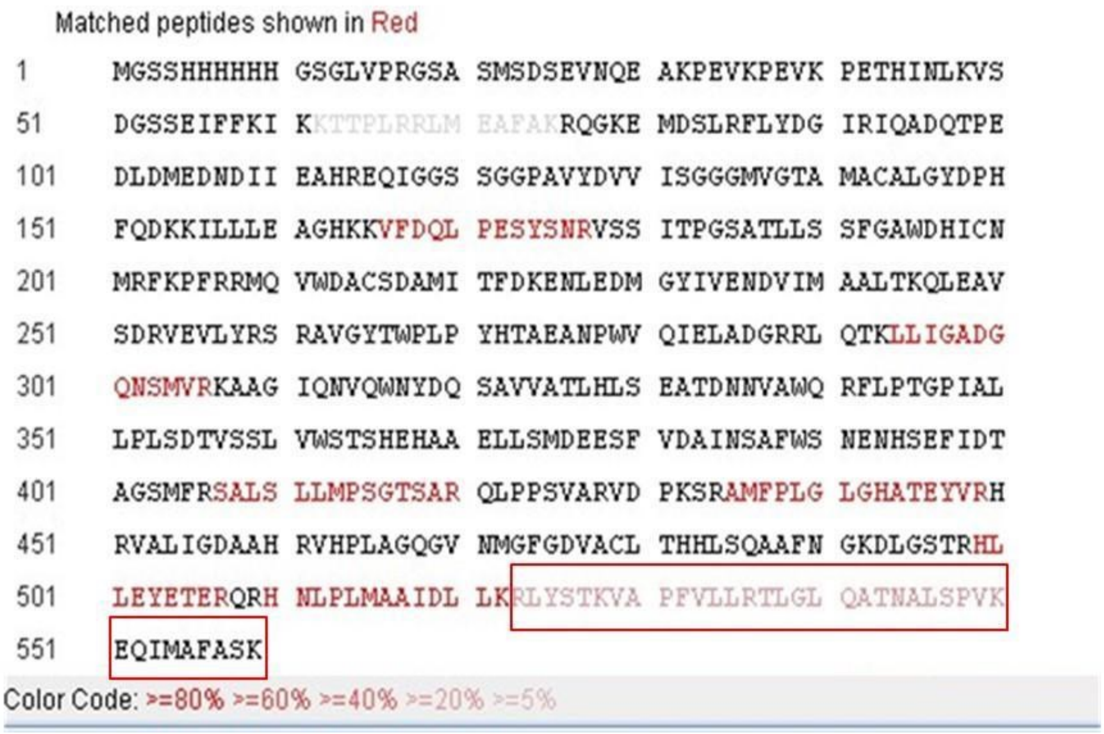

Supplementary Figure 3. Limited proteolysis of COQ6 analysed by UHPLC-HRMS and peptide mapping. The peptide in the red box was removed in the COQ6 construct employed for most of the experimental work.

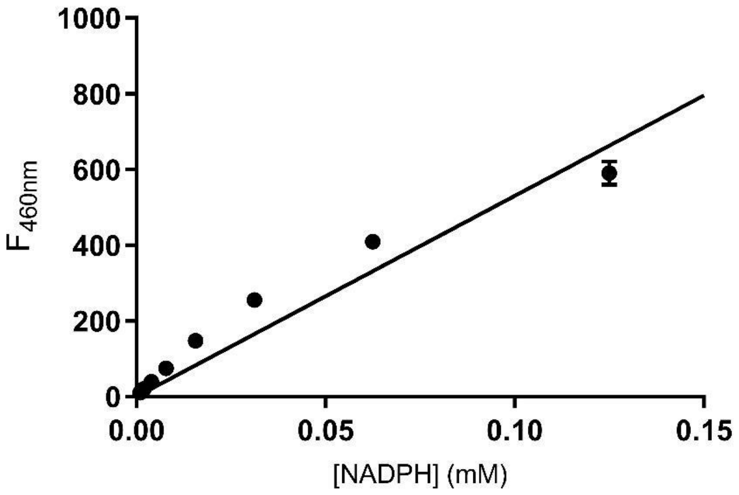

Supplementary Figure 4. Fluorescence NAD(P)H calibration line built by measuring $F_{460\,nm}$ of known concentrations of NAD(P)H. All data are presented as mean values ± s.d. The error bars correspond to the standard deviations in n = 2 independent measurements for each datum.

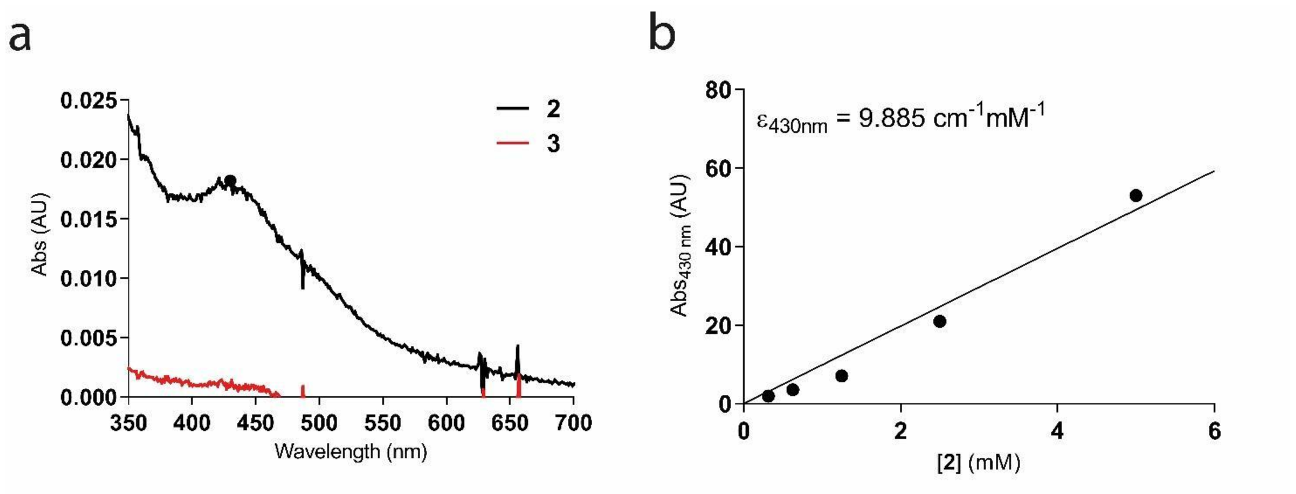

Supplementary Figure 5. UV/Vis spectra of 2 and 3. a. Visible spectra of **2** and **3**; the absorption peak at 430 nm disappears after O-methylation in position 5. **b.** Calibration line for the absorbance of **2** at 430 nm at known concentrations determined an extinction coefficient of 9.924 $cm^{-1}mM^{-1}$.

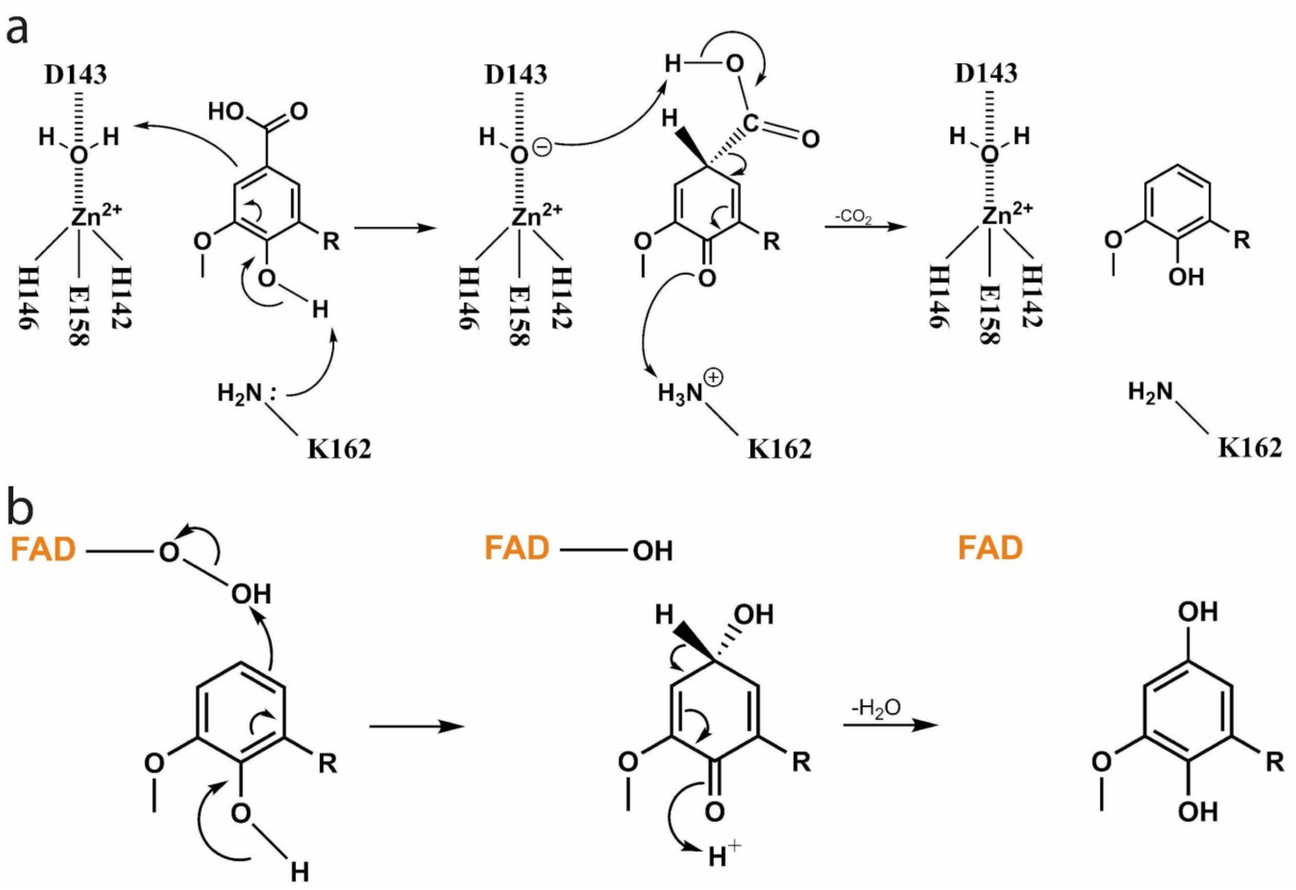

Supplementary Figure 6. COQ4 and COQ6 proposed mechanisms of action for C_1 modifications. a. Proposed mechanism for the Zn-dependent decarboxylase activity of COQ4 based on the inspection of the predicted AlphaFold model and mutagenesis experiments. **b.** Proposed mechanism for C_1-hydroxylase activity of COQ6, based on previous literature on flavin dependent monooxygenases [25,27].

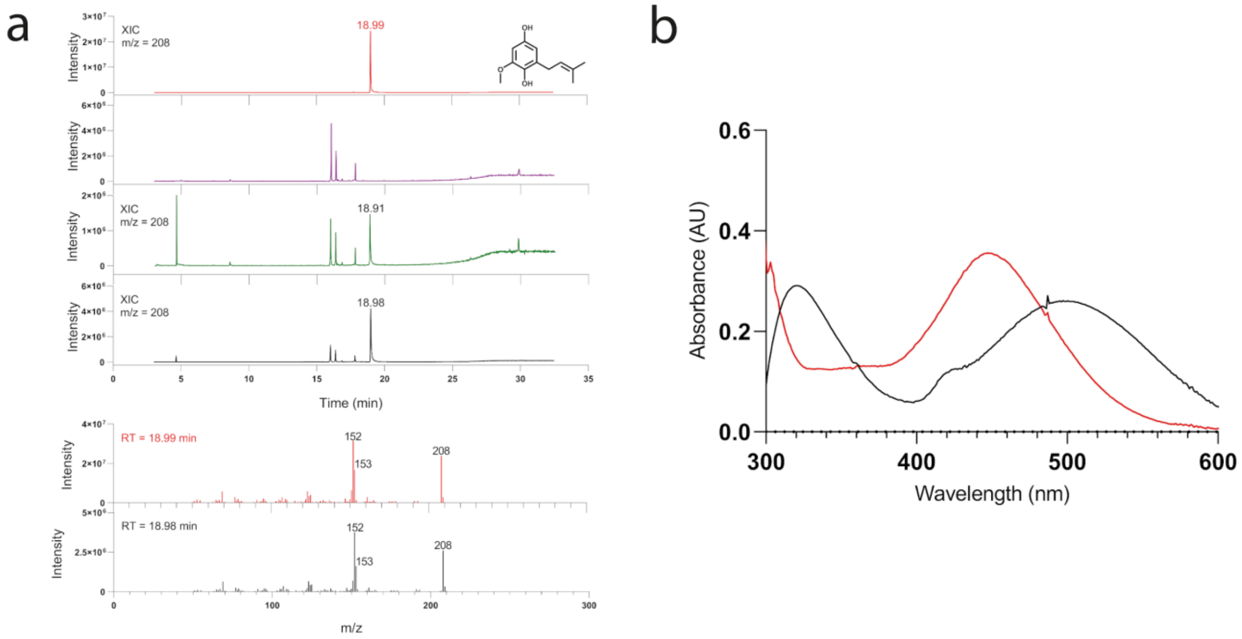

Supplementary Figure 7. COQ6 is responsible for C_1-hydroxylation. a. Comparison of the GC/MS analysis of the the overnight reaction of **4a** to **4b** with the blank injection. The extracted-ion chromatograms of **4b** recorded after the injection of 500 ppm analytical standard (red), blank injection (violet), reactions performed by truncated (green) and full-length (black) COQ6 are shown above. The full-scan mass spectra of corresponding to the peak of **4b** in the standard injection (red) and in the reaction by full-length COQ6 (black) are shown below. **b.** Visible spectra of 4-aminoantipyrine adducts with the analytical standards of **4a** (black) and **4b** (red).

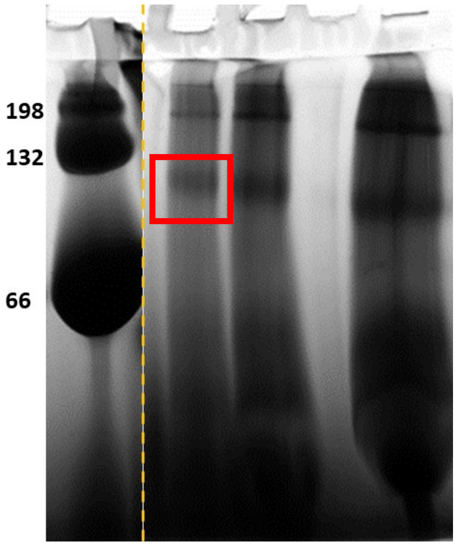

Supplementary Figure 8. Native PAGE analysis of the purified COQ metabolon. The gel filtered metabolon (see Extended Data Fig. 7) was submitted to Native PAGE analysis (14% polyacrylamide) at increasing concentrations (from left to right, 10, 25 and 50 µM, respectively, see Supplementary Fig. 16). The two top bands visible in the gel were found to contain all the COQ proteins as shown by peptide mapping (see Supplementary Table 2). In-house MW marker was prepared by vortexing Bovine Serum Albumin and generating dimeric and trimeric oligomeric states. Dashed lines indicate where lanes have been moved and rearranged in the gel to ease visual comparison; original gels can be found in Supplementary Figure 16. Native-PAGE analyses were repeated in n = 2 independent experiments.

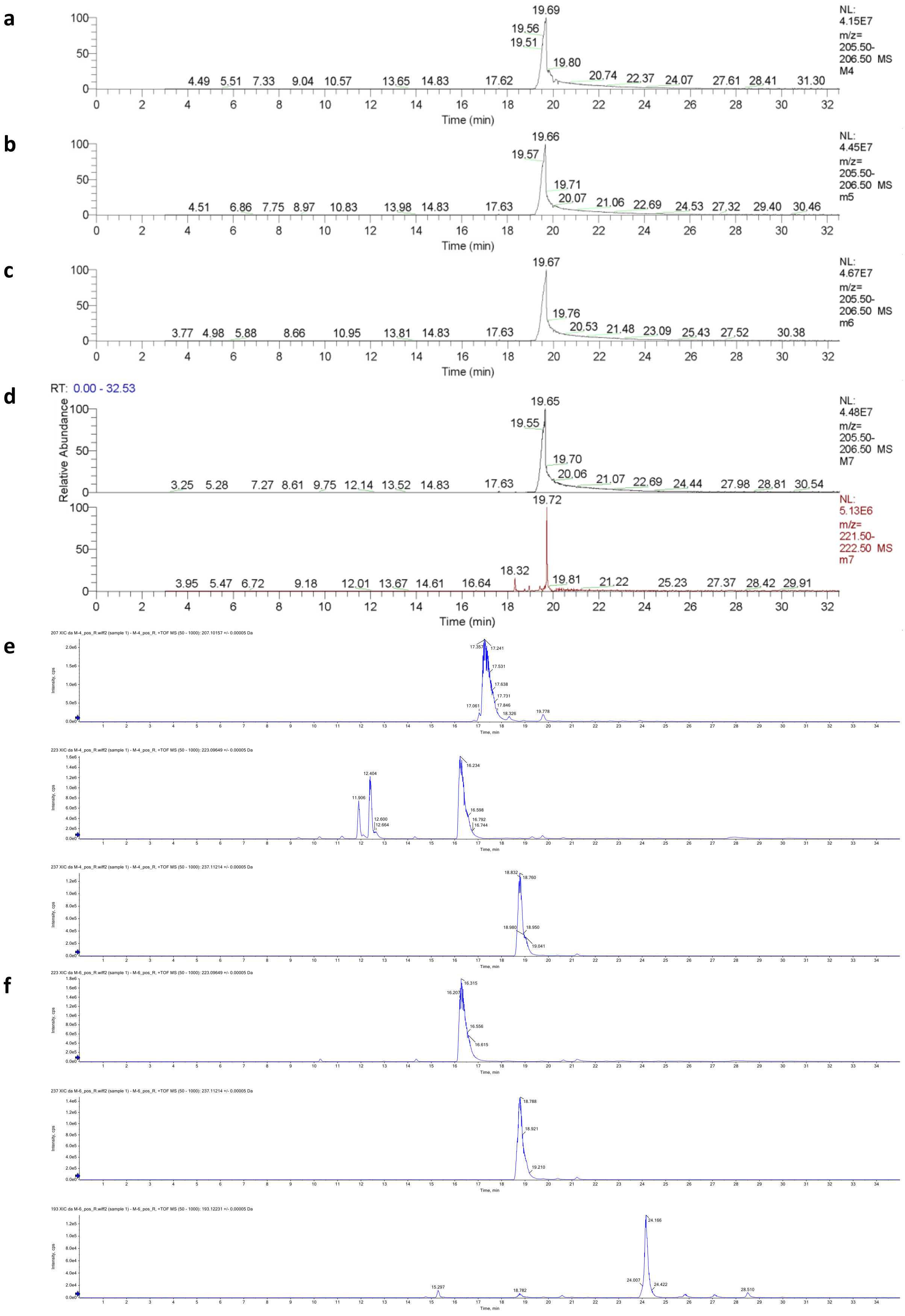
a
19.69
19.56
19.51
19.80
4.49 5.51 7.33 9.04 10.57 13.65 14.83 17.62 20.74 22.37 24.07 27.61 28.41 31.30
NL: 4.15E7 m/z= 205.50-206.50 MS M4
Time (min)
b
19.66
19.57
19.71
20.07 21.06 22.69 24.53 27.32 29.40 30.46
4.51 6.86 7.75 8.97 10.83 13.98 14.83 17.63
NL: 4.45E7 m/z= 205.50-206.50 MS m5
Time (min)
c
19.67
19.76
3.77 4.98 5.88 8.66 10.95 13.81 14.83 17.63 20.53 21.48 23.09 25.43 27.52 30.38
NL: 4.67E7 m/z= 205.50-206.50 MS m6
Time (min)
d
RT: 0.00 - 32.53
Relative Abundance
19.65
19.55
19.70
20.06 21.07 22.69 24.44 27.98 28.81 30.54
3.25 5.28 7.27 8.61 9.75 12.14 13.52 14.83 17.63
NL: 4.48E7 m/z= 205.50-206.50 MS M7
19.72
18.32
19.81 21.22 25.23 27.37 28.42 29.91
3.95 5.47 6.72 9.18 12.01 13.67 14.61 16.64
NL: 5.13E6 m/z= 221.50-222.50 MS m7
Time (min)
e
f

g

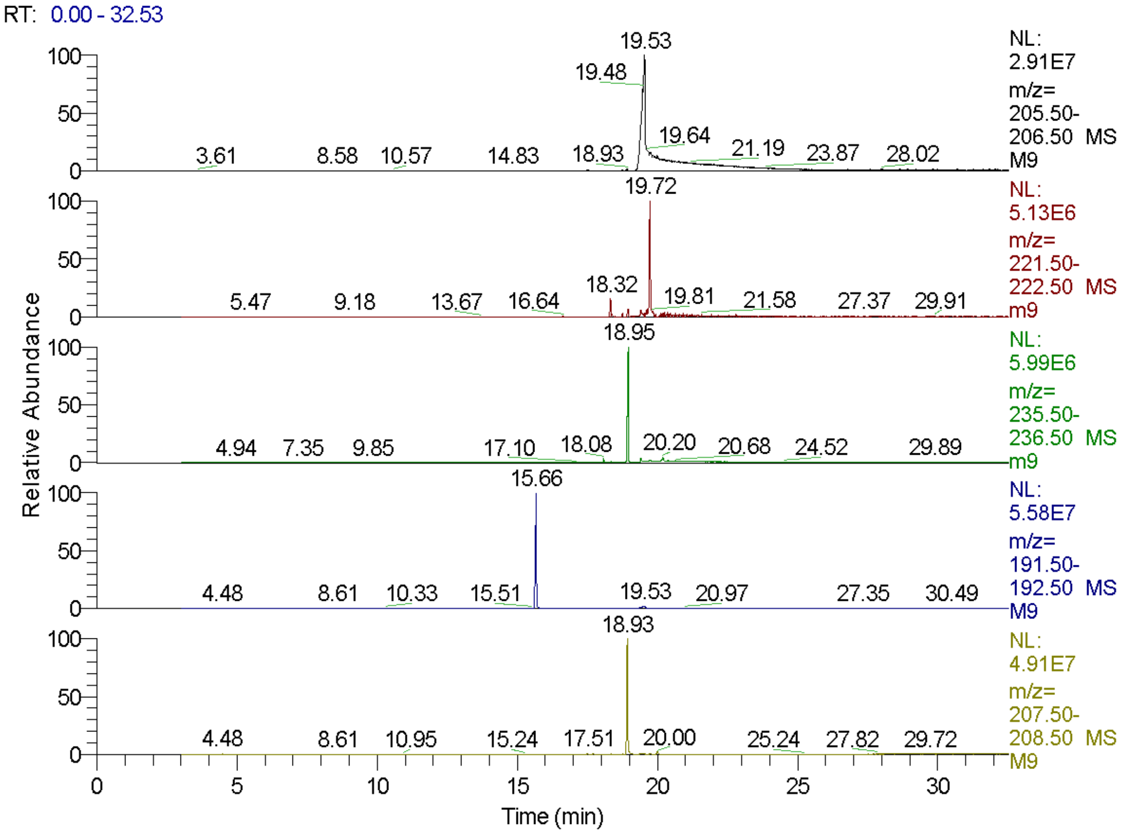
RT: 0.00 - 32.53
Relative Abundance
Time (min)
NL: 2.91E7 m/z= 205.50-206.50 MS M9
NL: 5.13E6 m/z= 221.50-222.50 MS m9
NL: 5.99E6 m/z= 235.50-236.50 MS m9
NL: 5.58E7 m/z= 191.50-192.50 MS M9
NL: 4.91E7 m/z= 207.50-208.50 MS M9
19.53
19.48
19.72
18.95
15.66
18.93

h

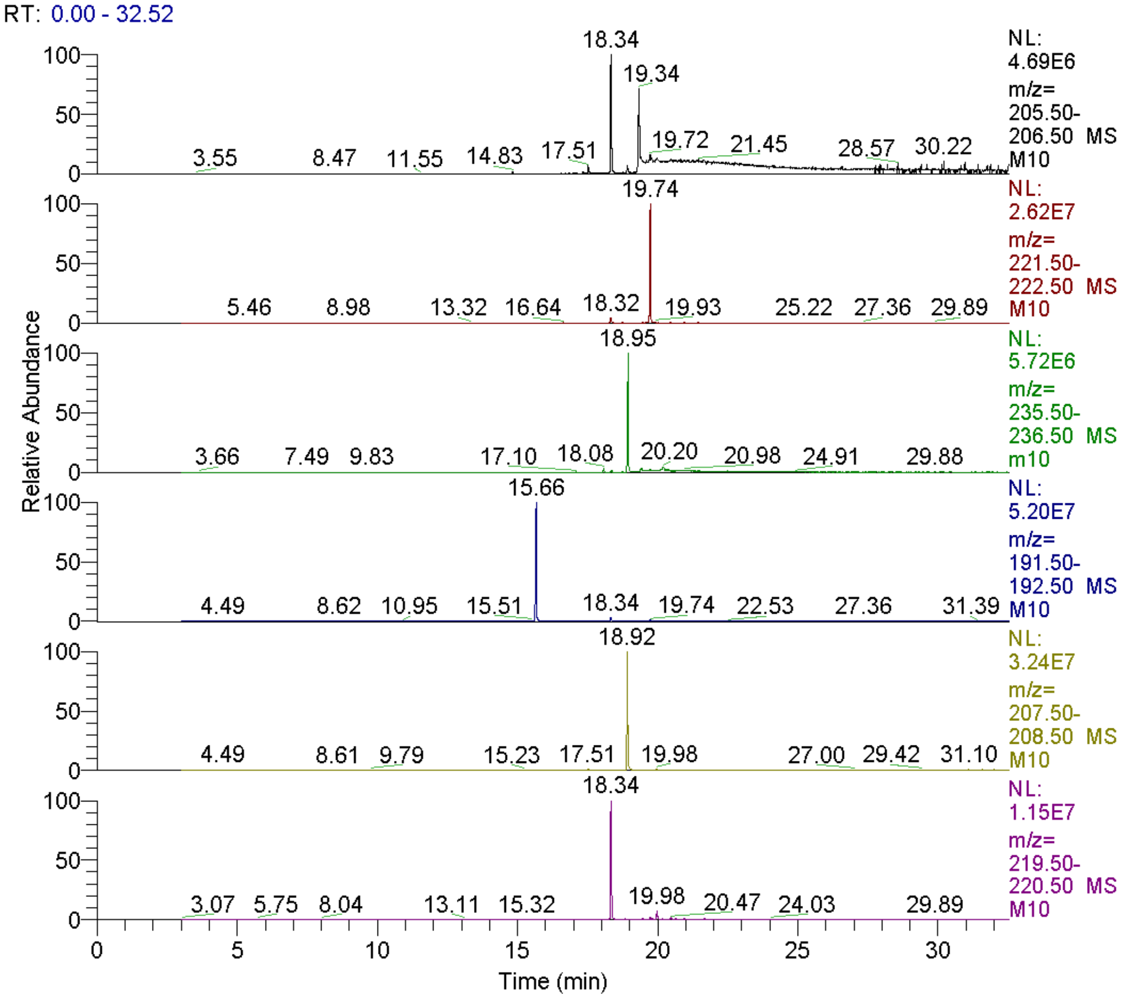
RT: 0.00 - 32.52
Relative Abundance
Time (min)
NL: 4.69E6 m/z= 205.50-206.50 MS M10
NL: 2.62E7 m/z= 221.50-222.50 MS M10
NL: 5.72E6 m/z= 235.50-236.50 MS m10
NL: 5.20E7 m/z= 191.50-192.50 MS M10
NL: 3.24E7 m/z= 207.50-208.50 MS M10
NL: 1.15E7 m/z= 219.50-220.50 MS M10
18.34
19.34
19.74
18.95
15.66
18.92
18.34

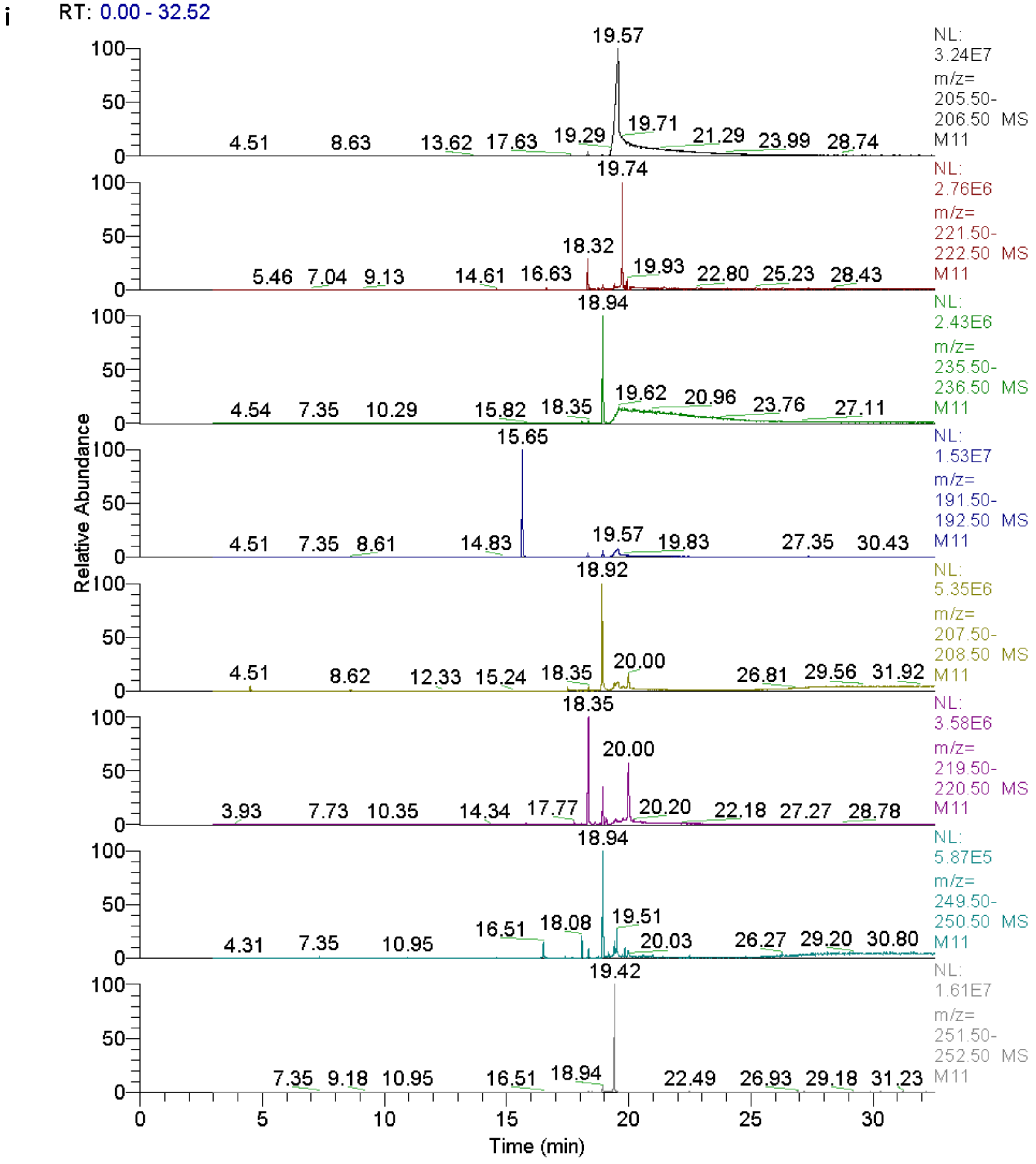

Supplementary Figure 9. Chromatograms of experiments reported in Figure 6b. **a.** Metabolon without FDXR: from top to bottom, GC XIC of **1** (m/z=206). **b.** Metabolon without FDX2: GC XIC of **1** (m/z=206). **c.** Metabolon without COQ6: GC XIC of **1** (m/z=206). **d.** Metabolon without COQ3: GC XIC of **1** (m/z=206) and **2** (m/z=222). **e.** Metabolon without COQ4: UHPLC/ESI+ XIC of **1** (m/z=207), **2** (m/z=223) and **3** (m/z=237). **f.** Metabolon without COQ6 starting from **2**: UHPLC/ESI+ XIC of **2** (m/z=223), **3** (m/z=237) and **4a** (m/z=193). **g**. Metabolon without COQ5: GC XIC of **1** (m/z=206), **2** (m/z=222), **3** (m/z=236), **4a** (m/z=192) and **4b** (m/z=208). **h.** Metabolon without COQ7: GC XIC of **1** (m/z=206), **2** (m/z=222), **3** (m/z=236), **4a** (m/z=192), **4b** (m/z=208) and **5** (m/z=220). **i.** Metabolon without COQ9: GC XIC of **1** (m/z=206), **2** (m/z=222), **3** (m/z=236), **4a** (m/z=192), **4b** (m/z=208), **5** (m/z=220), CoQ_1 (m/z=250) and CoQ_1H_2 (m/z=252).

a

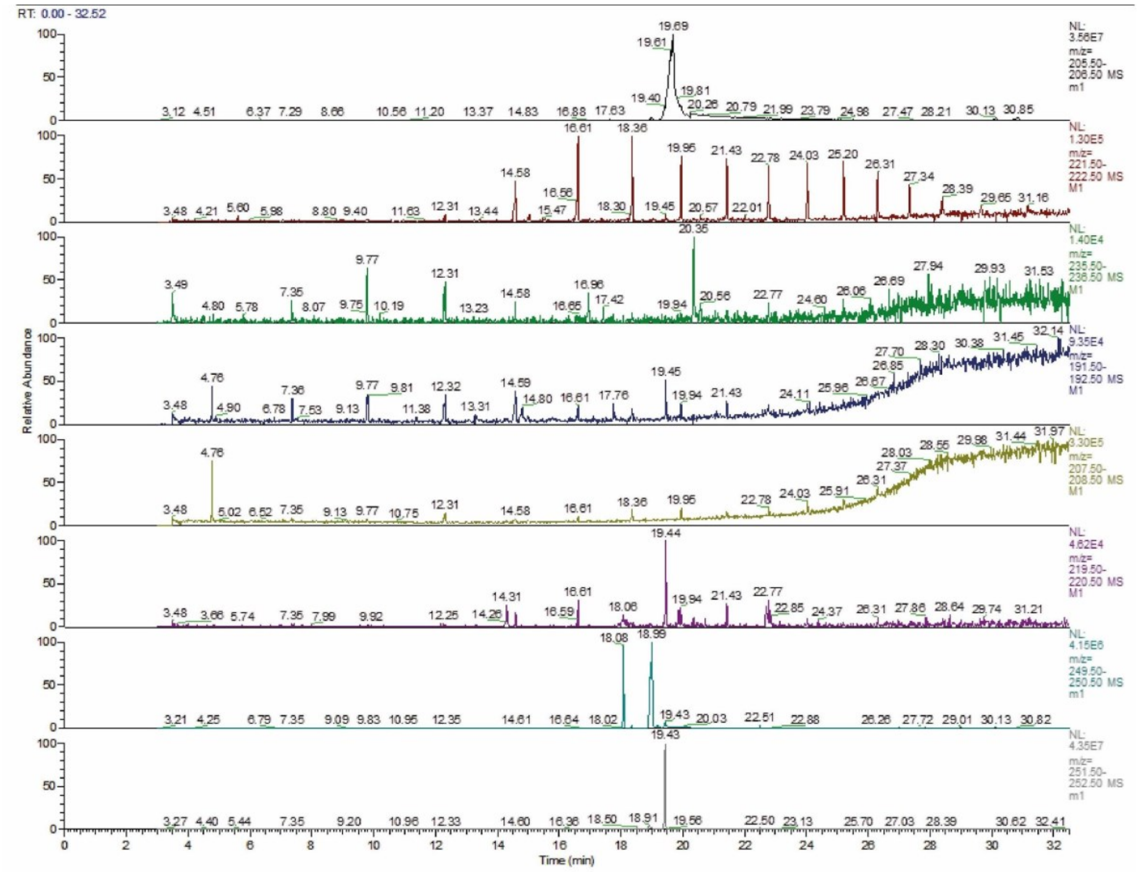
RT: 0.00 - 32.52
Relative Abundance
Time (min)
NL: 3.56E7 m/z= 205.50-206.50 MS m1
NL: 1.30E5 m/z= 221.50-222.50 MS M1
NL: 1.40E4 m/z= 235.50-236.50 MS M1
NL: 9.35E4 m/z= 191.50-192.50 MS M1
NL: 3.30E5 m/z= 207.50-208.50 MS M1
NL: 4.62E4 m/z= 219.50-220.50 MS M1
NL: 4.15E6 m/z= 249.50-250.50 MS m1
NL: 4.35E7 m/z= 251.50-252.50 MS m1

b

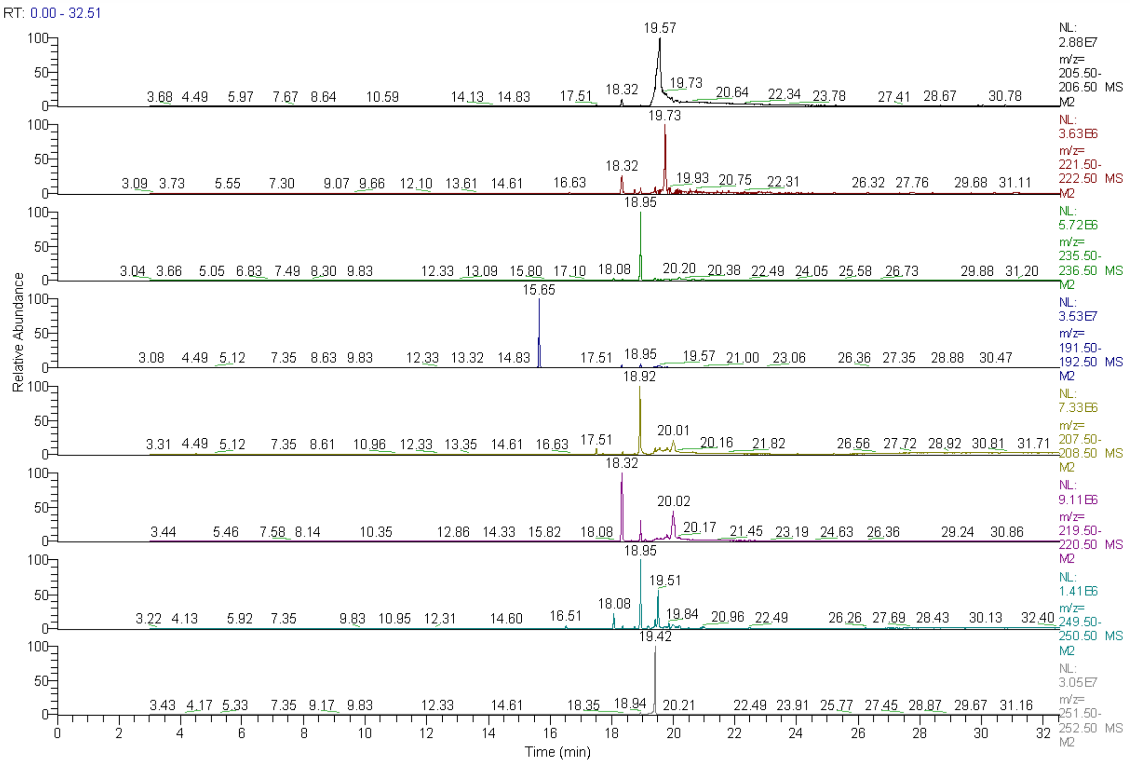
RT: 0.00 - 32.51
Relative Abundance
Time (min)
NL: 2.88E7 m/z= 205.50-206.50 MS M2
NL: 3.63E6 m/z= 221.50-222.50 MS M2
NL: 5.72E6 m/z= 235.50-236.50 MS M2
NL: 3.53E7 m/z= 191.50-192.50 MS M2
NL: 7.33E6 m/z= 207.50-208.50 MS M2
NL: 9.11E6 m/z= 219.50-220.50 MS M2
NL: 1.41E6 m/z= 249.50-250.50 MS M2
NL: 3.05E7 m/z= 251.50-252.50 MS M2

c

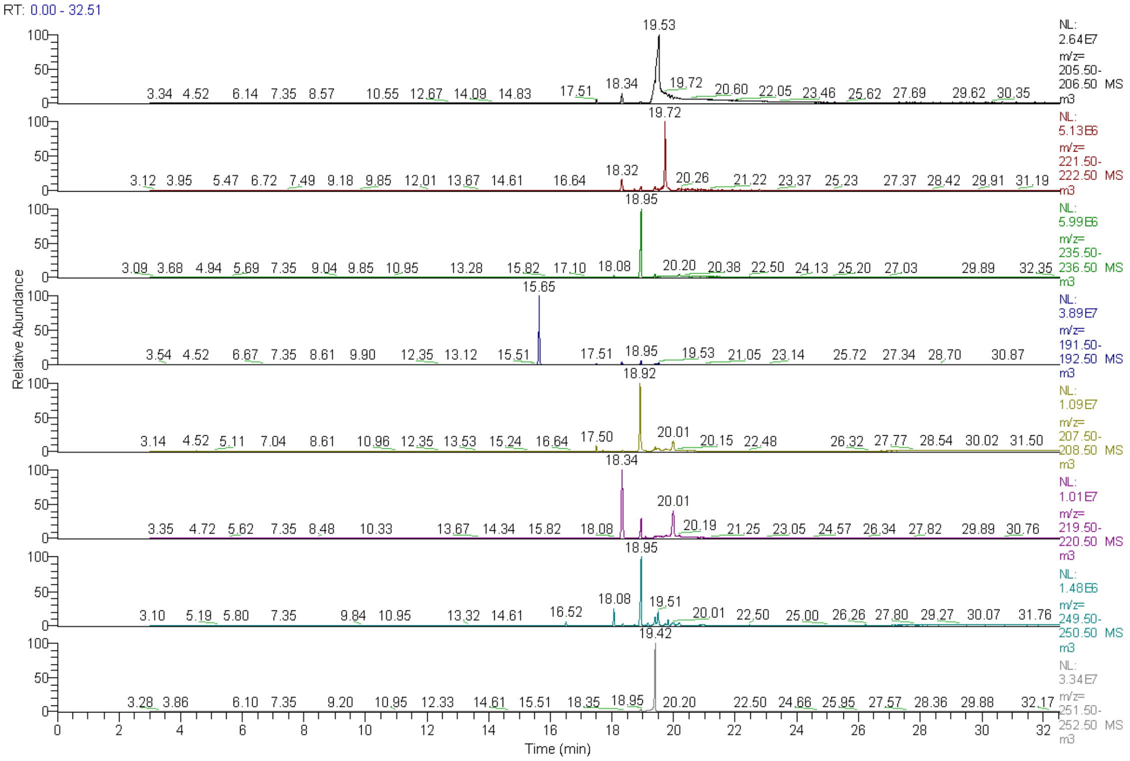

d

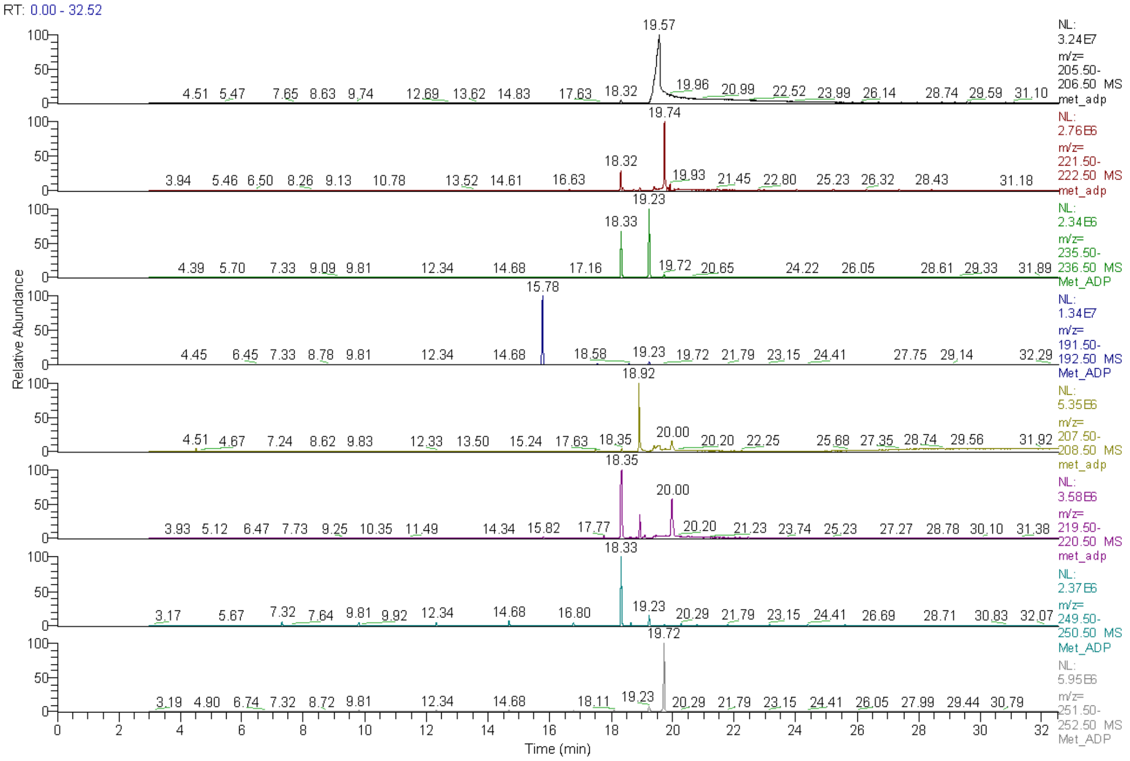

Supplementary Figure 10. Chromatograms of experiments reported in Figure 6c. a. Metabolon with COQ8B and ATP: from top to bottom, GC XIC of **1** (m/z=206), **2** (m/z=222), **3** (m/z=236), **4a** (m/z=192), **4b** (m/z=208), **5** (m/z=220), CoQ_1 (m/z=250) and CoQ_1H_2 (m/z=252). **b.** Metabolon with COQ8B without ATP: GC XIC of **1** (m/z=206), **2** (m/z=222), **3** (m/z=236), **4a** (m/z=192), **4b** (m/z=208), **5** (m/z=220), CoQ_1 (m/z=250) and CoQ_1H_2 (m/z=252). **c.** Metabolon without COQ8B and ATP: GC XIC of **1** (m/z=206), **2** (m/z=222), **3** (m/z=236), **4a** (m/z=192), **4b** (m/z=208), **5** (m/z=220), CoQ_1 (m/z=250) and CoQ_1H_2 (m/z=252). **d.** Metabolon with COQ8B and ADP: GC XIC of **1** (m/z=206), **2**

(m/z=222), **3** (m/z=236), **4a** (m/z=192), **4b** (m/z=208), **5** (m/z=220), CoQ_1 (m/z=250) and CoQ_1H_2 (m/z=252).

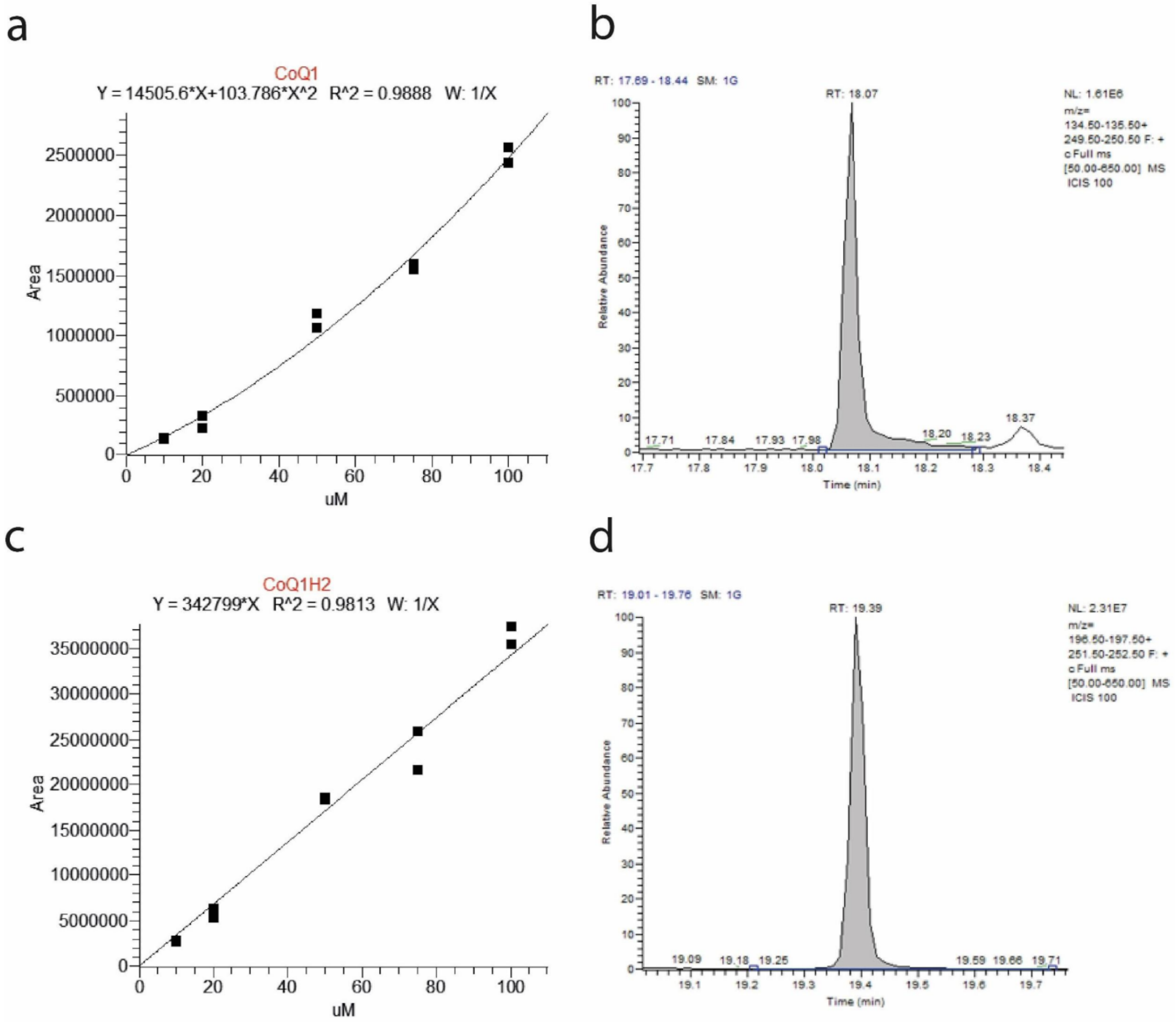

Supplementary Figure 11. Calibration curves and automated peak integration of $CoQ_1(H_2)$. a. Quadratic calibration curve employed for CoQ_1 quantitation. **b.** CoQ_1 peak integration showed for a 100 µM analytical standard. **c.** Linear calibration curve employed for CoQ_1H_2 quantitation. **d.** CoQ_1H_2 peak integration showed for a 100 µM analytical standard.

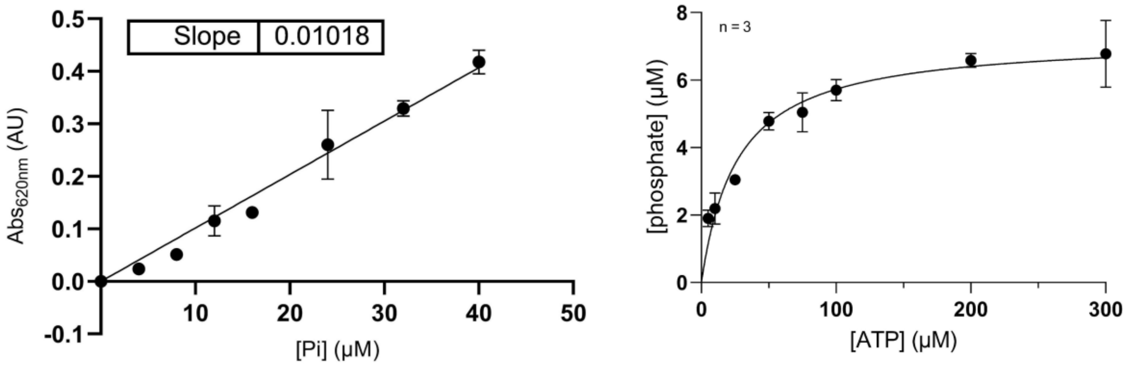

Supplementary Figure 12. Monitoring inorganic phosphate production using Malachite green assay. Calibration of the inorganic phosphate adduct with Malachite Green dye and Michaelis-Menten curve describing the K_M of ATP for COQ8B. All data are presented as mean values ± s.d. The error bars correspond to the standard deviations in n = 3 independent measurements for each datum.

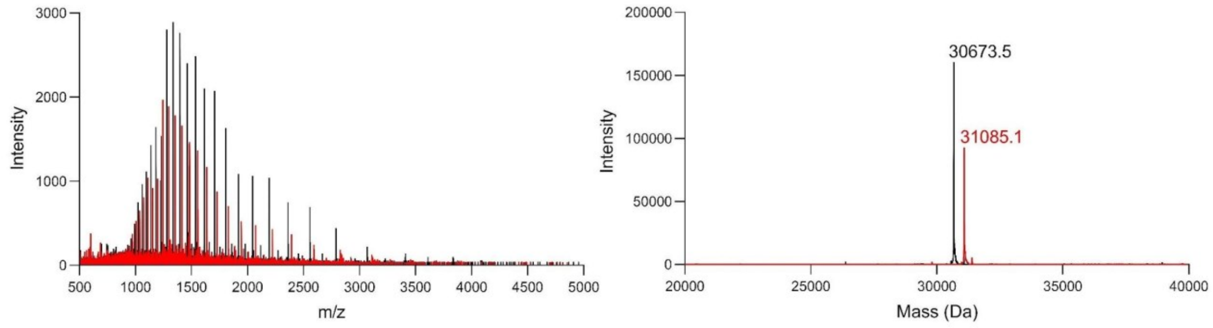

Supplementary Figure 13. COQ3 is phosphorylated by COQ8B. Intact protein mass-spectrometry analysis shows that COQ3 is phosphorylated by COQ8B in five positions. Raw and deconvoluted mass spectra collected after the injection of 50 µg of the reaction (red) and of the negative control (black) are shown.

COQ6

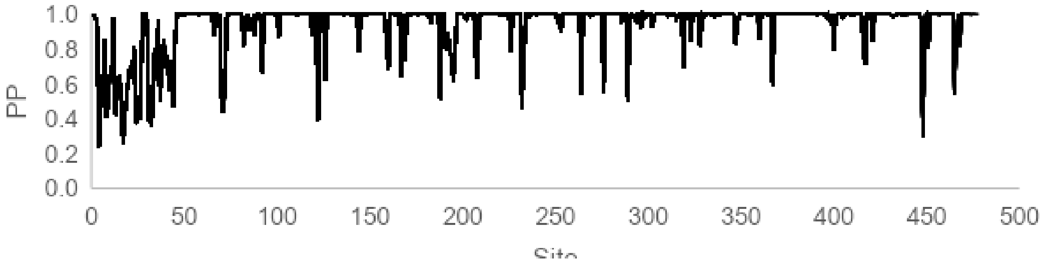

$\underline{PP} = 0.94$

Length= 478 amino acids

Ambiguously reconstructed sites (alternative state PP > 0.2) = 41 (8.6%)

Sequence identity to human COQ6 = 75.3%

Deposited sequence – tAncCOQ6_tr – with the N-terminal cleavage site shown in bold and red.

Accession code: OQ859713

>Tetrapod ancestor COQ6

MAARLGLSGWGRRRLRLRCGARLSLARRGLARCCRRR**S**SSGGPAVYDVVISGGGMVGTAMACALGYDPHFQDKKILLLEA
GHKKVFDQLPESYSNRVSSITPGSATLLSSFGAWDHICNMRFKPFRRMQVWDACSDAMITFDKENLEDMGYIVENDVIM
AALTKQLEAVSDRVEVLYRSRAVGYTWPLPYHTAEANPWVQIELADGRRLQTKLLIGADGQNSMVRKAAGIQNVQWNY
DQSAVVATLHLSEATDNNVAWQRFLPTGPIALLPLSDTVSSLVWSTSHEHAAELLSMDEESFVDAINSAFWSNENHSEFID
TAGSMFRSALSLLMPSGTSARQLPPSVARVDPKSRAMFPLGLGHATEYVRHRVALIGDAAHRVHPLAGQGVNMGFGDV
ACLTHHLSQAAFNGKDLGSTRHLLEYETERQRHNLPLMAAIDLLKRLYSTKVAPFVLLRTLGLQATNALSPVKEQIMAFASK

COQ3

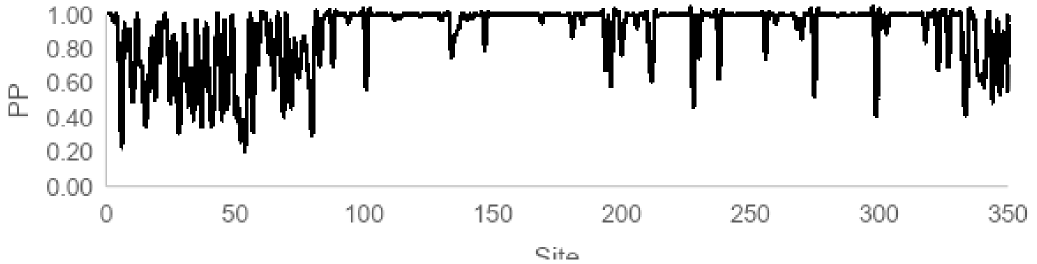

$\underline{PP} = 0.89$

Length= 362 amino acids

Ambiguously reconstructed sites (alternative state PP > 0.2) = 44 (12.15%)

Sequence identity to human COQ3 = 64.7%

Deposited sequence – tAncCOQ3_tr – N-terminal cleavage site shown in bold and red.

Accession code: OQ859710

>Tetrapod ancestor COQ3

MWGGGRGSRAGRLLVALRGRSRGRGAGCRRLSLAAAAGNHYGWTLQMAPRFKSSNRTMWLKSNSTTFA

SLTKMKSSRSAVKRM**Y**STSQTTVDPKEMKKFQALAHKWWDEQGEYAALHSMNDLRVPFIRDTLLNMSGDH

QLGSPLSGMKILDVGCGGGLLSEPLGRLGASVTGIDPLEDNIRTAELHKSFDPVLDKRIQYKACSLEEIVEEAT

ETFDAVVASEVVEHVADVETFIKCCYQVLKPGGSLFITTINKTQLSYALGIVVAERIMGIVPAGTHDWEKFISPE
ELERLLESNGFSVETVNGMLYNPLSGSWSWIENTSINYALHAVKSKVQEQSDSTEPPSEQEQEQHQAETST
STTV

COQ4

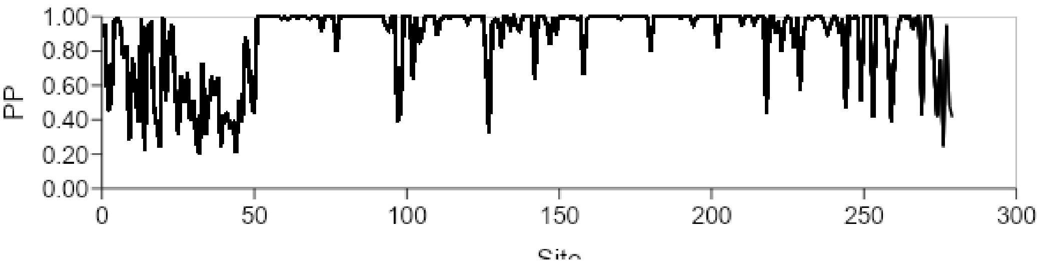

$\underline{PP} = 0.88$

Length= 279 amino acids

Ambiguously reconstructed sites (alternative state PP > 0.2) = 21 (7.5%)

Sequence identity to human COQ4 = 77%

Deposited sequence – tAncCOQ4_tr – N-terminal cleavage site shown in bold and red.

Accession code: OQ859711

>Tetrapod ancestor COQ4

MATLLRRARGLLPLLRPVLGSPGLTGRALCMRQA**P**AADSPLRAAEEGYGPLYPGHIPTSPLQKALLAAGSAC
MALYNPYRHDMVAVLGETTGHLALQNLRDRMRNDPEGYQILQERPRIRLSTLDLAKLRSLPDGSFGREYVR
FLDDNRVSPDTRAPVKFVDDEELAYVIQRYREVHDLLHTLLGMPTNMLGEVVVKWFEAVQTGLPMCILGAAF
GPIRLSARKLQVLVTELIPWAVQNGRNARCVLNIYYERRWEQSLESLREELGITPPPIRVTGLA

COQ5

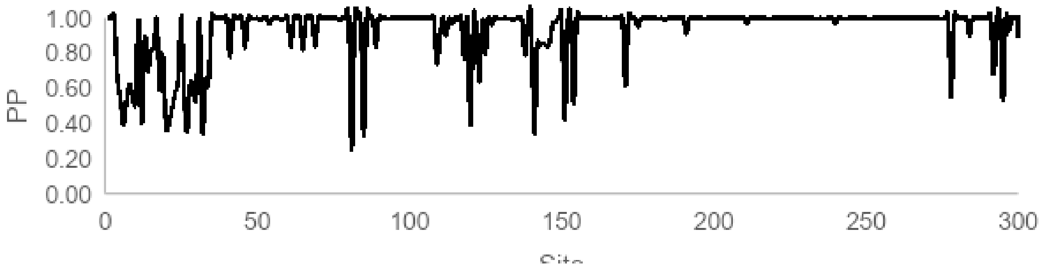

$\underline{PP} = 0.93$

Length= 312 amino acids

Ambiguously reconstructed sites (alternative state PP > 0.2) = 23 (7.4%)

Sequence identity to human COQ5 = 75%

Deposited sequence – tAncCOQ5_tr – N-terminal cleavage site shown in bold and red.

Accession code: OQ859712

>Tetrapod ancestor COQ5

MAASMRCACSRALCGCRSGARVCCRAHSTE**A**AEKETHFGFQTVSEEEKGEKVYQVFENVAKKYDIMNDAM
SLGIHRLWKDALLHQMNPYPGTQLLDVAGGTGDIAFRFINYVRSQRERQVRQELKSHQNLSWQEISKSYQE
EEQDSLGGSQAVICDINKEMLKVGKQKAQQLGYSEGLSWVVGNAEELPFDDDKFDVYTIAFGIRNVTHIDQA
LQEAYRVLKPGGRFLCLEFSQVNNPLISRLYDLYSFQVIPVLGEVIAGDWKSYQYLVESIRRFPSQEEFKAMIE
DAGFFKVEYHNLTSGIVAIHSGFKL

COQ7

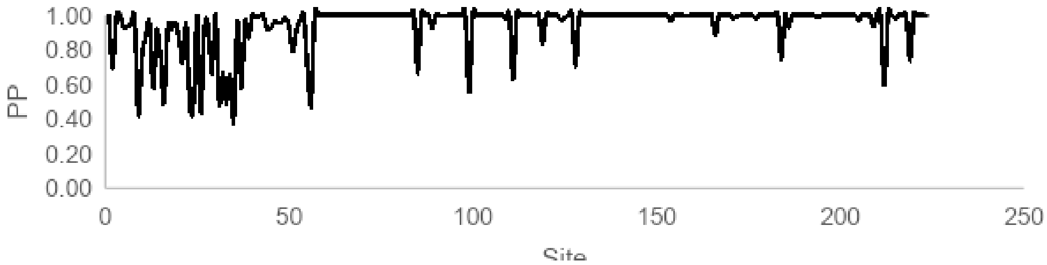

$\underline{PP} = 0.94$

Length= 224 amino acids

Ambiguously reconstructed sites (alternative state PP > 0.2) = 20 (8.9%)

Sequence identity to human COQ7 = 77.7%

Deposited sequence – tAncCOQ7_tr – N-terminal cleavage site shown in bold and red.

Accession code: OQ859714

>Tetrapod ancestor COQ7
MERAAAAAVRRGWRAHCRRLRLGAGPRRPCCAQARRTSVR**F**CSTGMTLDNVDKAVIDRII
RVDHAGEYGANRIYAGQMAVLGRTSVGPVIQQMWDQEKEHLKKFNELMVAHRVRPTILMP
FWNVAGFVLGAGTALLGKEGAMACTVAVEESISEHYNNQIRTLMEEDPEKYKELLQIIKK
FRDEELEHHDTGLEHDAELAPAYSLLKNVIQIGCKAAIYLSERI

COQ9

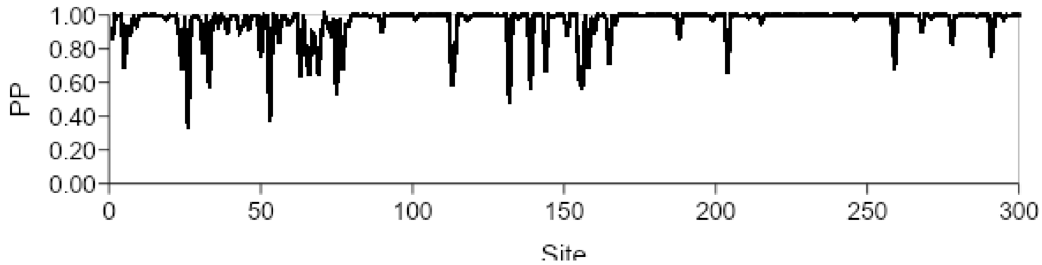

$\underline{PP} = 0.96$

Length= 318 amino acids

Ambiguously reconstructed sites (alternative state PP > 0.2) = 18 (5.7%)

Sequence identity to human COQ9 = 84.3%

Deposited sequence – tAncCOQ9_tr_N79 – N-terminal cleavage site shown in bold and red.

Accession code: OQ859717

>Tetrapod ancestor COQ9

MMAAAVAGLLRRAGWRLLQLRCRVVVRCQLSPVQRAFHASAVLRRVSDEQKQQPPPSSSQQHSESQPTE
EPDPESQRS**H**PSYTDQGGEESEDYESEEQLQHRILTAALEFVPEHGWTAEAIAEGAKSLGLSAAAAGMFGN
DGSDLILHFVSQCNSKLSELLEEEHKLVQLGQAEKKKTDQFLRDAVEARLRMLIPYIEKWPQALSILLLPHNIP
ASLNLLTSMVDDMWHYAGDQSTDINWYTRRAVLAGIYNTTELVMMQDSSPDFEDTWRFLENRINDAMNMG
HTAKQVKSTGEALVQGLMGAAVTLKNLTGLNQRR

COQ8A

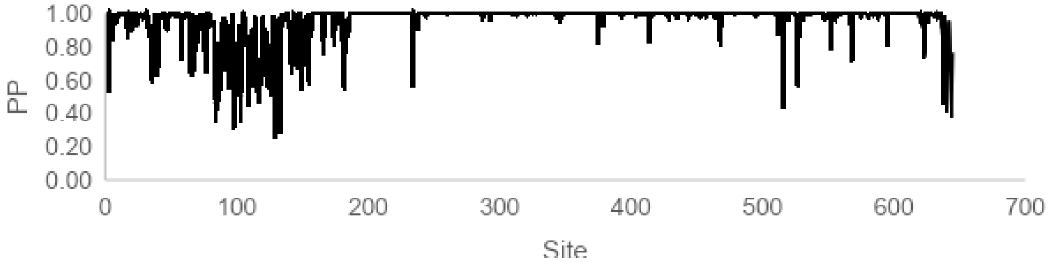

$\underline{PP} = 0.95$

Length= 645 amino acids

Ambiguously reconstructed sites (alternative state PP > 0.2) = 47 (7.3%)

Sequence identity to human COQ8A = 73.9%

Deposited sequence – tAncCOQ8A_tr – N-terminal cleavage site shown in bold and red.

Accession code: OQ859715

>Tetrapod ancestor COQ8A

MAGDAIMLMRGLAKLSKAVLETQAGQLRLGGEAVAIARTWQATAEEGFSAAMGKMQELGKQQENLSDIGE
DFGSEYDFSGPESSSANKDFSSPSGQPHEHSGAEGPAYSYATNGPFRNTGDSSRADSPVSAKGNGKLFG
GFRDPGNPFAAA**F**GQTRAFHQDHSSVGGLTAEDIEKAREAKANPENKPHKQMLSERARERKVPVTRIGRLA
NFGGLAVGLGIGALAEVAKKSLRPEERNGKKAVLDSSPFLSEANAERIVRTLCKVRGAALKLGQMLSIQDDAF
INPQLQKIFERVRQSADFMPIKQMMKTLNNDLGPNWRDKLEFFEERPFAAASIGQVHLARLKDGREVAMKIQ
YPGVAQSINSDVNNLMTVLSMSNALPEGLFPEHLIEVLSRELALECDYKREAACAKKFKELLKDHPFFYVPAV
VDELCSQHVLTTELVSGFPLDQAEGLSQEIRNEICHNILVLCLRELFEFRFMQTDPNWSNFFYDPQLHKVALL
DFGATRGFDEDFTDIYIEVIKAAADQDRERVLKKSIEMKFLTGYESKAMENAHLDAVLILGEAFASEEPFDFGS
QSTTERIHGLIPVMLKHRLVPPPEETYSLHRKMGGSFLICSKLKAKIPCKNMFQEAYSKYWSRRAKKQEQ

COQ8B

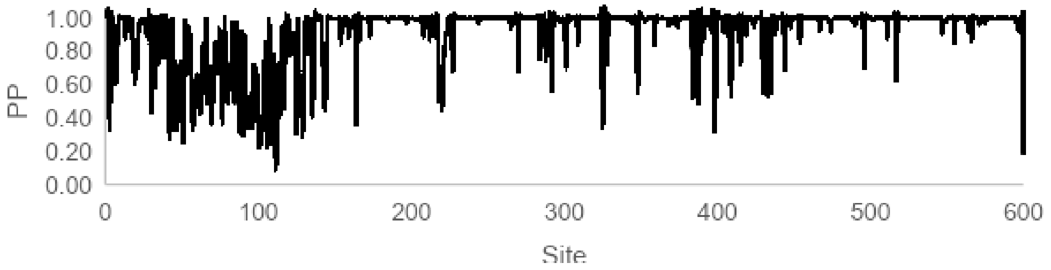

$\underline{PP} = 0.89$

Length= 629 amino acids

Ambiguously reconstructed sites (alternative state PP > 0.2) = 77 (12.2%)

Sequence identity to human COQ8B = 63.8%

Deposited sequence – tAncCOQ8B_tr – N-terminal cleavage site shown in bold and red.

Accession code: OQ859716

>Tetrapod ancestor COQ8B

MWSEVGSVLRGAGRVGQAFAETQGEQLRLMARSSALGAGLKRAQESVEQCLSSLLASRQRGARDEFSEA
SEEEDASRWGVASEMPPDISLPEAAAGAGSAQSPGGRPHPPAHGARGPGWPSGSPSFSGRGPGMGQTR
SFHQ**D**AAVRGLTAEDIKKAREAKQKQSKPPRQKLSERARERKVPASRISRLANFGGLAVSLGLGALAEVAKK
SLNGEQKPKDTRSLLDSSPFLSEANAERIVDTLCKVRGAALKIGQMLSIQDNSFISPQLQKIFERVRQSADFM
PAWQMMKVLAEELGPDWREKLASFEERPFAAASIGQVHLGVLRDGREVAMKIQYPGIAQSIRSDVDNLLSVL
KMSVVLPEGLFADNSIQVLQRELEWECDYKREAACARRFRQLLKDDPFFYVPEVIDELTTKRVLTMELVSGV
PLDQCVGLDQDIRNEICFNILRLCLRELFEFRFMQTDPNWSNFFYDAEKHKVTLLDFGASREFGKEFTDHYIE
VVKAAADGDRAKVLQKSKDLKFLTGFETKVFEEAHVDAVMILGEAFASPEPFDFGTQNTTRRIQNLIPVMLKH
RLTPPPEESYSLHRKMAGSFLICAKLGAVIPCREMFQEIYGRYWARERAAPLEAATA

FDXR

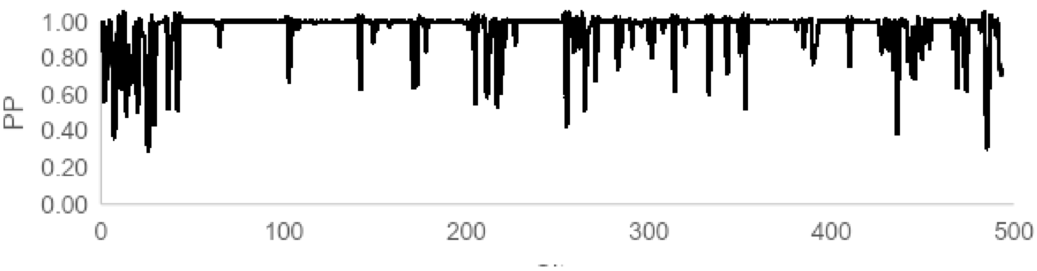

$\underline{PP} = 0.94$

Length= 494 amino acids

Ambiguously reconstructed sites (alternative state PP > 0.2) = 47 (9.5%)

Sequence identity to human FDXR = 73%

Deposited sequence – tAncFDXR_tr – N-terminal cleavage site shown in bold and red.

Accession code: OQ859718

>Tetrapod ancestor FDXR

MGAPRGAVCWLWGVRSLARSLPRAGSPGVRRL**L**STASPTPQICIVGSGPAGFYTAQHLLKHHKQAQVDIYE
KLPVPFGLVRFGVAPDHPEVKNVINTFTQTAHSDRCSFYGNVTVGKDVTVEELQQAYHAVVLSYGAEDNRT
LGIPGENLPGVYSARAFVGWYNGLPENRDLNPDLSSETAVILGQGNVALDVARILLSPLDLLKKTDITQHSLEA
LAQSKVKRVWLVGRRGPLQVAFTIKELREMINLPGTRPLLDPSDFEGLGDAIKDLPRPRKRLTELMIKTALEK
PGEKEAARWASATREWGLRFLRSPVEVLPSADGKRAAGIRLAVTRLEGSGESARAVPTGEVEDIECGLILSSI

GYKSLPIDPSVPFDPKQGIIPNSMGRVQGAPGLYCSGWVKRGPTGVIITTMNDSFDTAQSVLEDLQSGVLDV
SAPKPGFQAIRALLQQRGVHPVSFSDWEKIDAAETARGKAVGKPREKILDVEEMLQLASQ

FDX2 - tAncFDX2

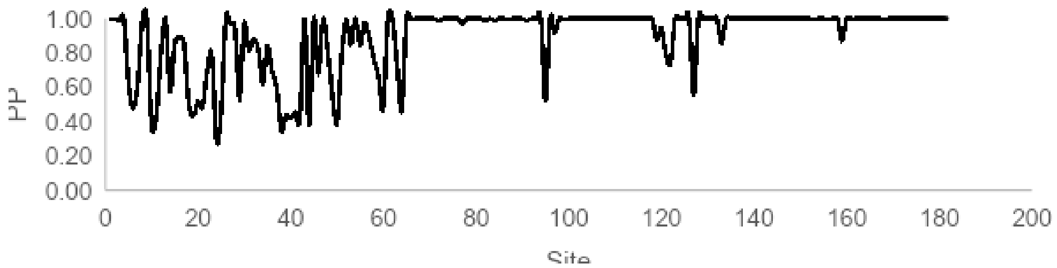

$\underline{PP} = 0.89$

Length= 182 amino acids

Ambiguously reconstructed sites (alternative state PP > 0.2) = 22 (12.1%)

Sequence identity to human FDX2 = 70.5%

Deposited sequence – tAncFDX2_tr – N-terminal cleavage site shown in bold and red.

Accession code: OQ859719

>Tetrapod ancestor FDX2

MAASMVARGVAAGCLLRACGGCLFSRPGGCRRRSRAASRAPARGFQTA**G**AHQAEEESQAPEPSDDVVNV
VFIDRSGQRIPVKGKVGDNVLYLAHKHGIDLEGACEASLACSTCHVYVSEEFLDKLPEPDEREDDMLDMAPL
LQENSRLGCQIILTKELEGAEFTLPKITRNFYVDGHVPKPH

Supplementary Figure 14. List of reconstructed sequences. Each ancestral sequence used in this study is shown separately including: the posterior probability for reconstruction, final amino acid length, the number of ambiguously reconstructed sites, sequence identity compared to the human orthologue, accession code and sequence description, and final sequence. The amino acid labelled in red corresponds to the final residue removed in the truncated construct. The graphs shown illustrate the posterior probability for the reconstructed amino acid at each site across the polypeptide. The final posterior probability for the entire reconstruction is shown as PP.

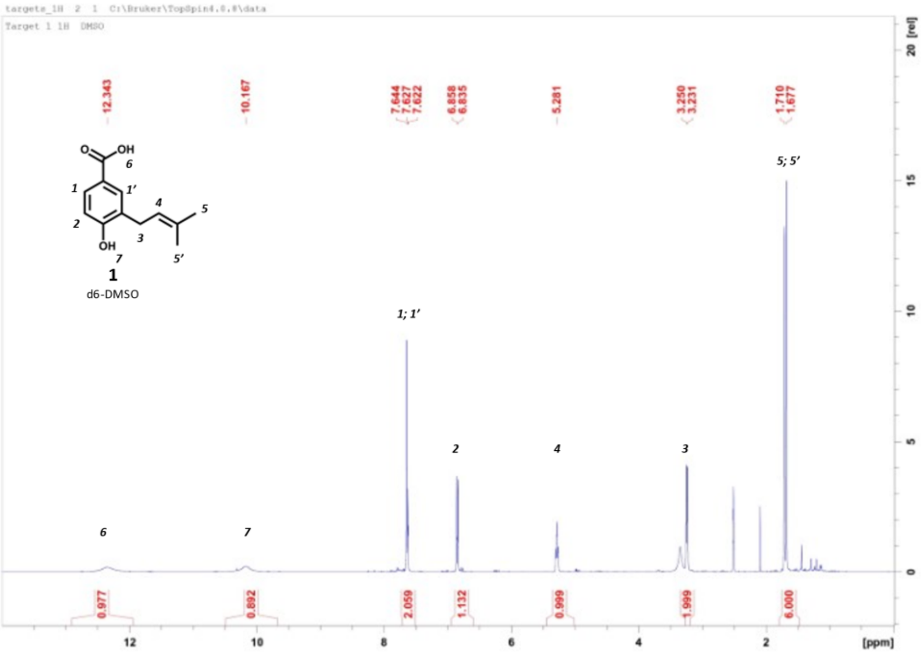
Target 1 1H DMSO
12.343
10.167
7.644
7.627
7.622
6.858
6.835
5.281
3.250
3.231
1.710
1.677
1
d6-DMSO
1; 1'
2
4
3
5; 5'
6
7
0.977
0.892
2.059
1.132
0.999
1.999
6.000
[ppm]
[rel]

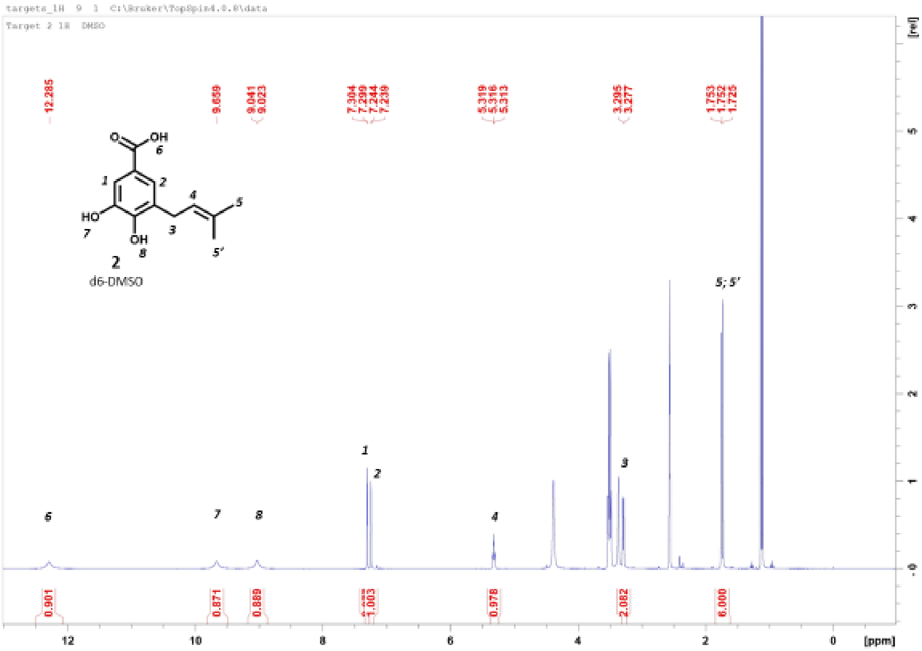
Target 2 1H DMSO
12.285
9.659
9.041
9.023
7.304
7.299
7.244
7.239
5.319
5.316
5.313
3.295
3.277
1.753
1.752
1.725
2
d6-DMSO
5; 5'
1
2
3
4
6
7
8
0.901
0.871
0.889
1.003
0.978
2.082
6.000
[ppm]
[rel]

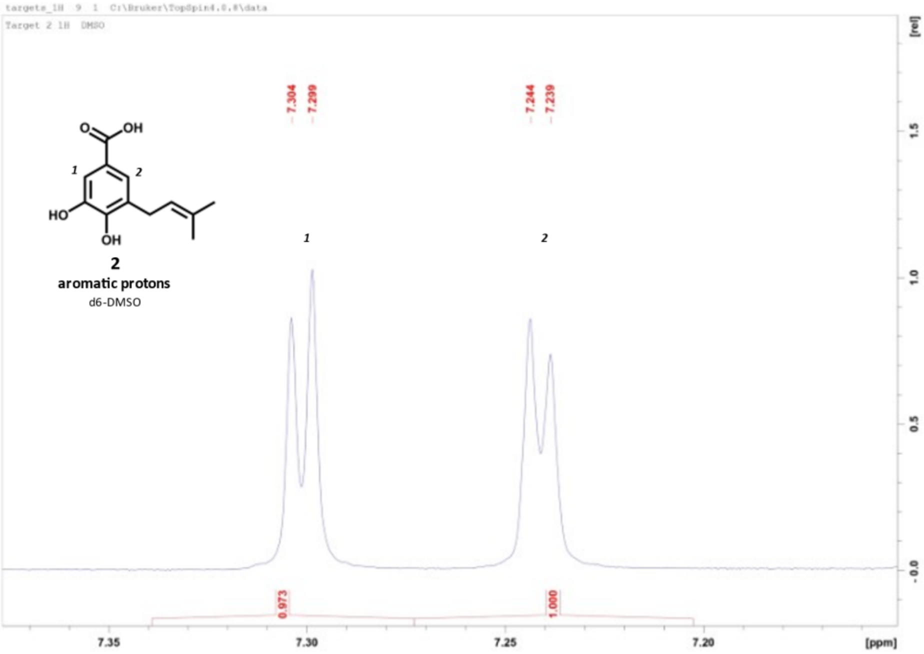
targets_1H 9 1 C:\Bruker\TopSpin4.0.8\data
Target 2 1H DMSO
2
aromatic protons
d6-DMSO
7.304
7.299
7.244
7.239
0.973
1.000
[ppm]
[rel]

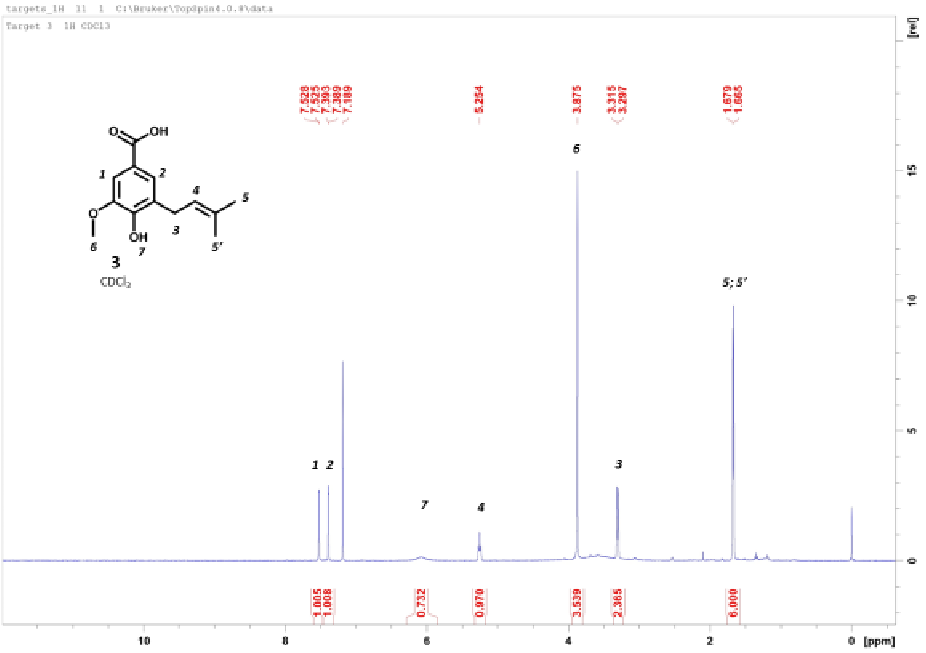
targets_1H 11 1 C:\Bruker\TopSpin4.0.8\data
Target 3 1H CDCl3
3
$CDCl_3$
7.528
7.525
7.393
7.389
7.189
5.254
3.875
3.315
3.297
1.679
1.665
1.005
1.008
0.732
0.970
3.539
2.365
6.000
[ppm]
[rel]

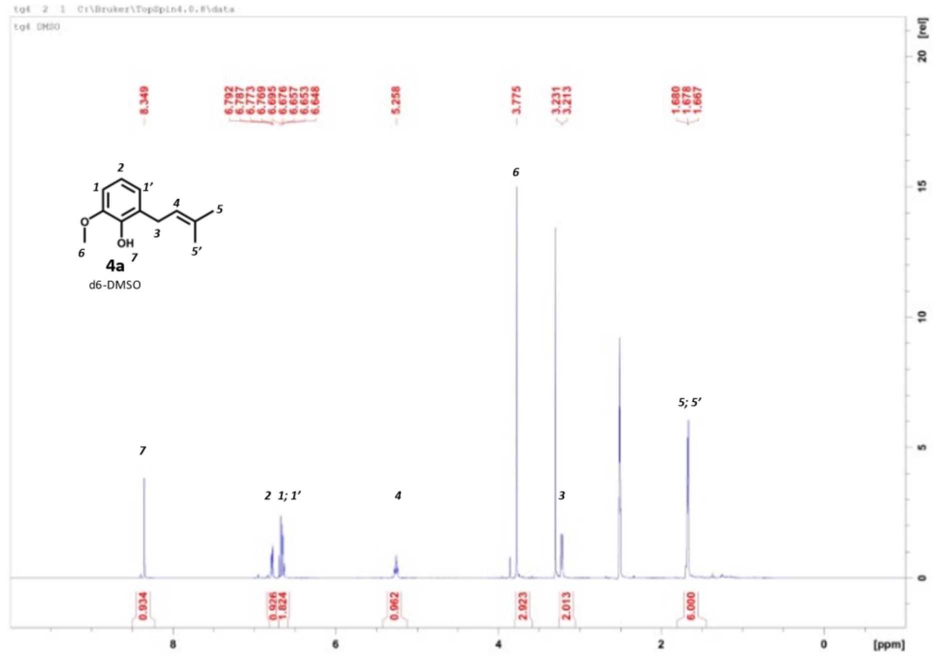

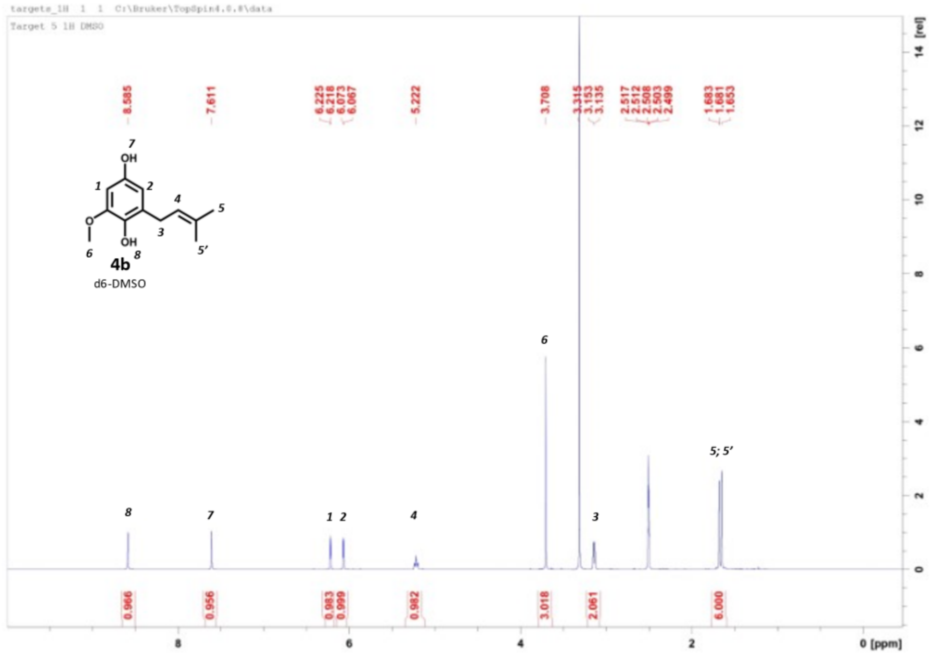

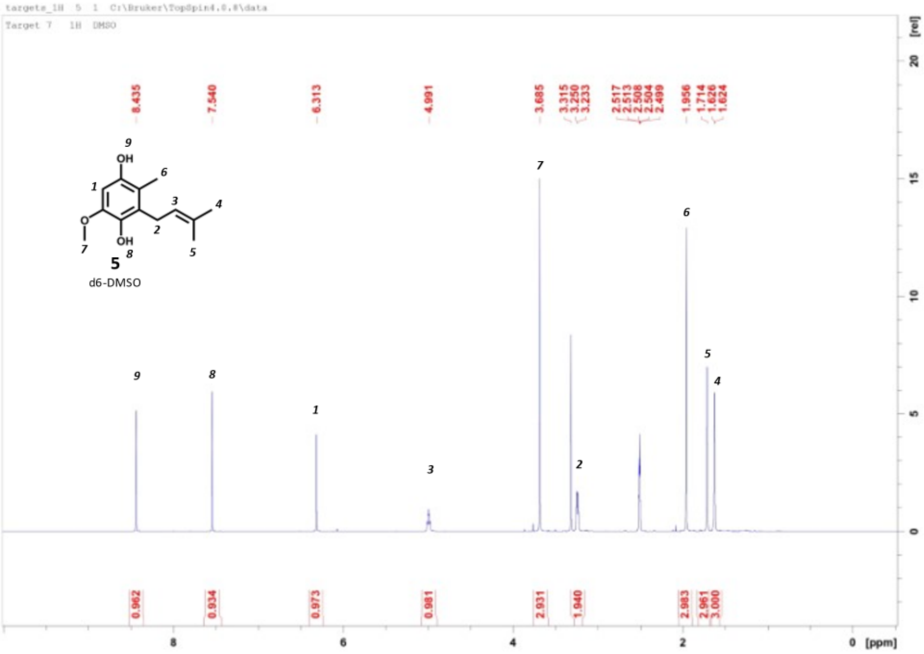
targets_1H 5 1 C:\Bruker\TopSpin4.0.8\data
Target 7 1H DMSO
5
d6-DMSO
[ppm]
[rel]

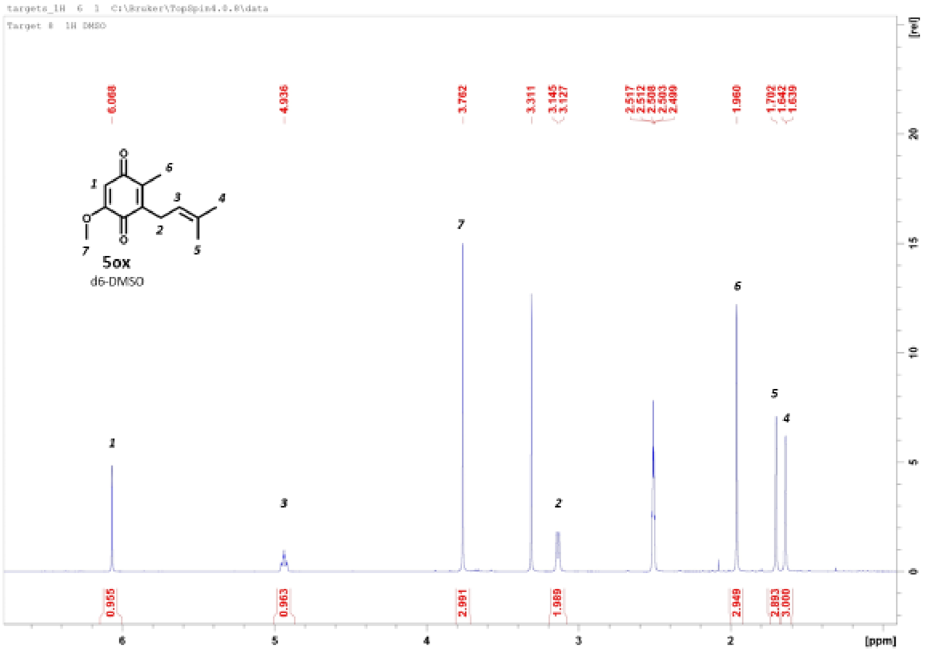
targets_1H 6 1 C:\Bruker\TopSpin4.0.8\data
Target 8 1H DMSO
5ox
d6-DMSO
[ppm]
[rel]

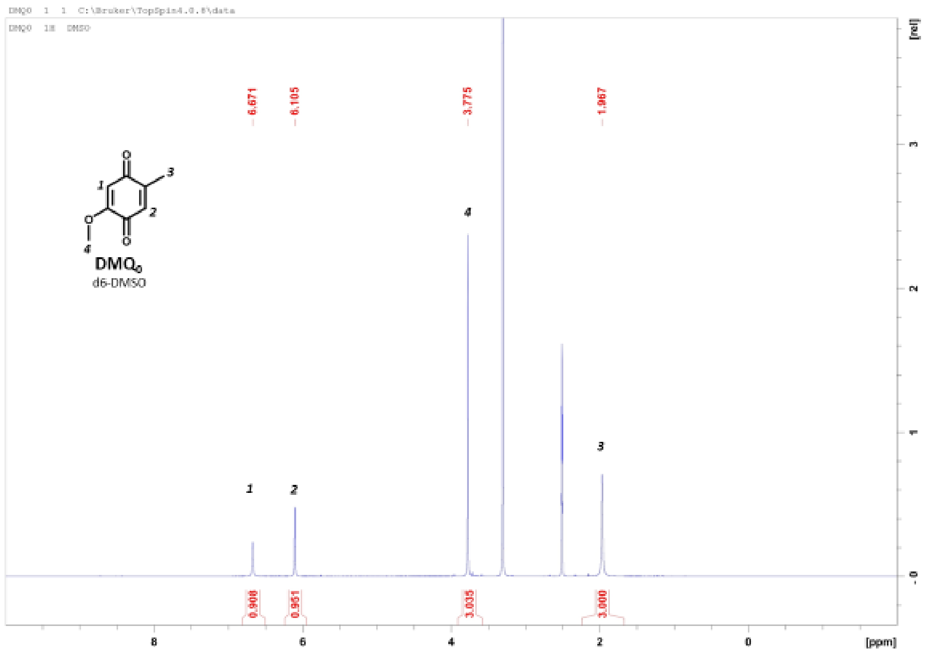

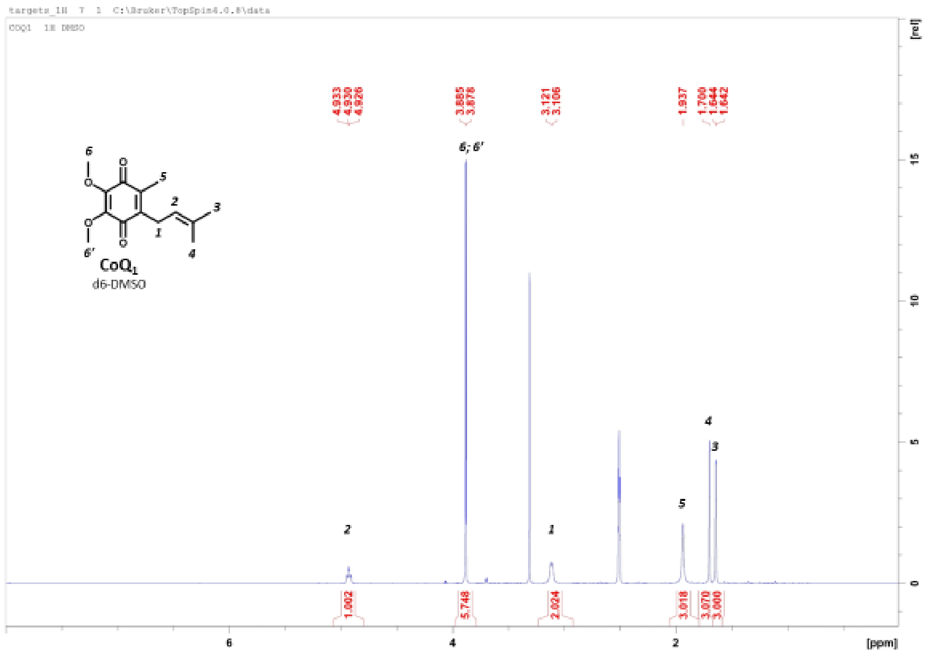

Supplementary Figure 15. NMR spectra of the CoQ compounds. NMR spectra of all compounds employed in this study: **1**; **2**; close-up spectrum for the aromatic H atoms illustrating their *J* coupling (*J* – 2 Hz); **3**; **4a**; **4b**; **5**; $\mathbf{5_{ox}}$; $\mathbf{5_{ox}}$ non-prenylated (denoted as $\mathbf{5_{ox0}}$); CoQ$_1$.

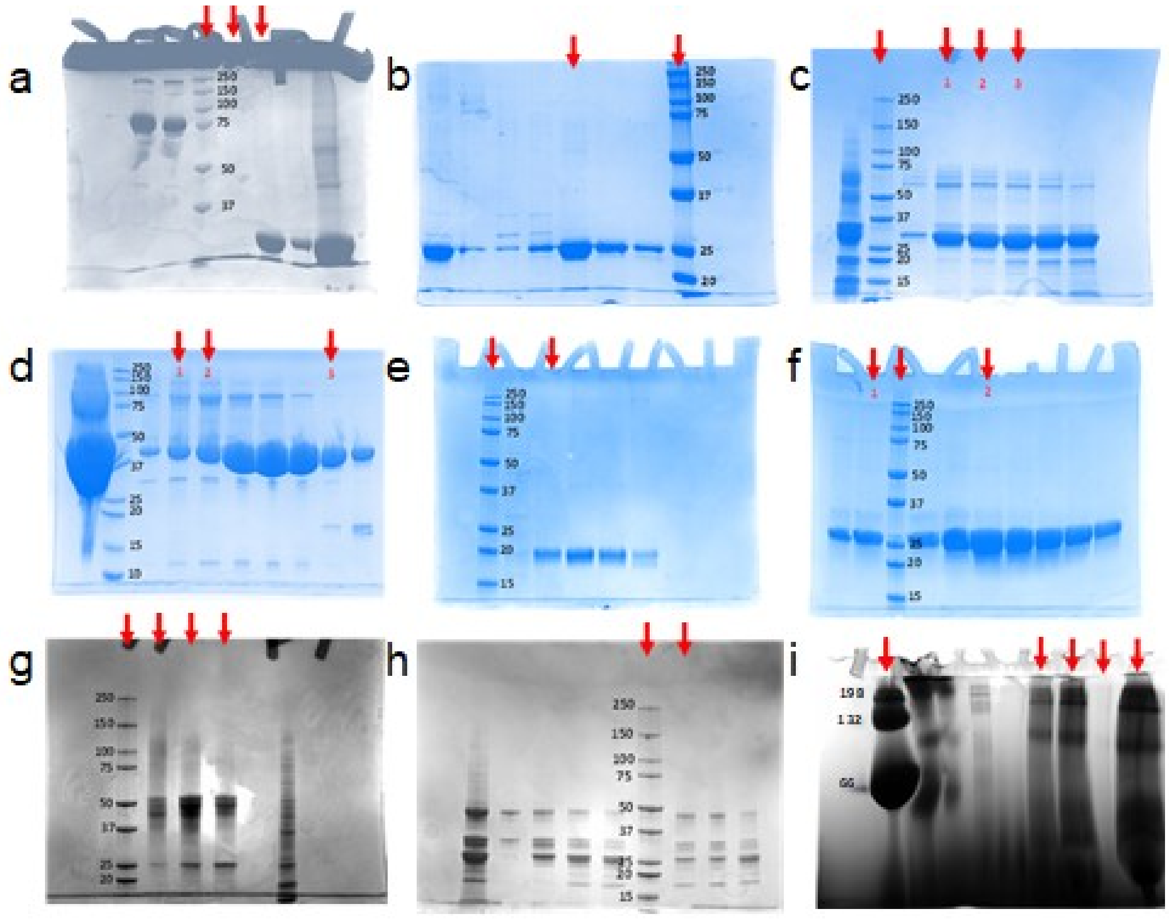

Supplementary Figure 16. SDS- and Blue Native-PAGE gels. Each lane used in a separate figure is indicated with a red arrow. The molecular weight ladder has its bands labelled with their individual respective molecular weights (kDa) shown. Where necessary, gels that have individual lanes cut out and reassembled for a separate figure are labelled and numbered in red in accordance with their respective figure panel. Expected molecular weights for each COQ protein are shown in Supplementary Table 2, except for COQ8B which has an expected molecular weight of 55.553 kDa. Panels a-g can be found in Extended Data Fig. 1; Panel h is displayed in Extended Data Fig. 7; Panel i is present in Supplementary Fig. 8. **a.** SDS-PAGE analysis of COQ3 after size exclusion chromatography. **b.** SDS-PAGE analysis of COQ4 after size exclusion chromatography. **c.** SDS-PAGE analysis of COQ5 after size exclusion chromatography. **d.** SDS-PAGE analysis of COQ6 truncated after size exclusion chromatography. Due to the presence of a large band skewing the molecular weight ladder, in Extended Data Fig. 1d an identical ladder is used to depict the molecular weights **e.** SDS-PAGE analysis of COQ7 after size exclusion chromatography. **f.** SDS-PAGE analysis of COQ9 after size exclusion chromatography. **g.** SDS-PAGE analysis of COQ8B after size exclusion chromatography. **h.** SDS-PAGE analysis of the COQ metabolon after size exclusion chromatography. **i.** Blue Native-PAGE analysis of the COQ metabolon after size exclusion chromatography.

Supplementary Tables

Target mutation	Mutagenesis cycle number	Target strand	Sequence
H142A	1	F	AGAGAAGTAGCGGACTTATTGCATACGTTACTTGG
H142A	1	R	CAATAAGTCCGCTACTTCTCTATAACGCTGGATCAC
H146A	2	F	GACTTATTGGCGACGTTACTTGGCATGCCAACTAA
H146A	2	R	AAGTAACGTCGCCAATAAGTCGTGTACTTCTCTAT
D143A	1	F	GAAGTACACGCGTTATTGCATACGTTACTTGGCATG
D143A	1	R	ATGCAATAACGCGTGTACTTCTCTATAACGCTGG
E158A	2	F	ATGTTAGGAGCGGTCGTCGTAAAGTGGTTTGAAGC
E158A	2	R	TACGACGACCGCTCCTAACATATTAGTTGGCATG
Sequencing primer		F	AACAGAGTCTCGCCAGAC

Supplementary Table 1. List of primers employed for site-directed mutagenesis of COQ4 (GST construct).

Protein	m/z	z	Peptide	Mass (Da)	Mr (Calc)	Delta	Start	End
**COQ6**<br>Score 99% Coverage 32.3%<br>48.295 kDa	384.2254	2	RLYSTK	766.4362	766.4337	0.002502441	404	409
	425.743	2	FKPFRR	849.4715	849.49725	-0.025756836	84	89
	457.7953	2	VAPFVLLR	913.5761	913.5749	0.001159668	410	417
	511.7891	2	VALIGDAAHR	1021.564	1021.56683	-0.003234863	333	342
	528.2665	3	KVFDQLPESYSNR	1581.778	1581.7786	-0.000976563	46	58
	535.6498	3	HNLPLMAAIDLLKR	1603.928	1603.9233	0.004394531	391	404
	554.6213	3	AMFPLGLGHATEYVR	1660.842	1660.8396	0.002441406	316	330
	559.6355	3	QLEAVSDRVEVLYR	1675.885	1675.8892	-0.004394531	127	140
	561.3557	2	ILLLEAGHKK	1120.697	1120.6968	0	37	46
	595.2949	2	HLLEYETER	1188.575	1188.5776	-0.002319336	380	388
	687.3651	2	LLIGADGQNSMVR	1372.716	1372.7131	0.002441406	175	187
	695.8727	2	SALSLLMPSGTSAR	1389.731	1389.7285	0.002319336	288	301
	706.9097	2	TLGLQATNALSPVK	1411.805	1411.8032	0.001708984	418	431
	724.92	2	HNLPLMAAIDLLK	1447.826	1447.8223	0.003295898	391	403
	727.8485	2	VFDQLPESYSNR	1453.682	1453.6836	-0.001220703	47	58
	806.7721	3	TLGLQATNALSPVKEQIMAFASK	2417.294	2417.2986	-0.004150391	418	440
	838.95	2	QLEAVSDRVEVLYR	1675.885	1675.8892	-0.00378418	127	140
**COQ3**<br>Score 99%<br>Coverage 32.2%<br>31.086 kDa	316.2001	2	VPFIR	630.3857	630.3853	0.000366211	45	49
	349.6957	2	TAELHK	697.377	697.37585	0.001098633	103	108
	359.5301	3	SFDPVLDKR	1075.569	1075.5662	0.002441406	109	117
	407.7319	2	FQALAHK	813.4492	813.4497	-0.000488281	20	26
	518.5967	3	IMGIVPAGTHDWEK	1552.768	1552.7708	-0.002319336	190	203
	557.2978	3	LGASVTGIDPLEDNIR	1668.872	1668.8682	0.003417969	87	102
	560.2889	2	FISPEELER	1118.563	1118.5608	0.002563477	204	212
	760.4287	2	TQLSYALGIVVAER	1518.843	1518.8403	0.002441406	176	189
	835.4404	2	LGASVTGIDPLEDNIR	1668.866	1668.8682	-0.001953125	87	102
	1051.008	2	DTLLNMSGDHQLGSPLSGMK	2100.001	2099.998	0.002441406	50	69
**COQ4**<br>Score 99%<br>Coverage 23.7%<br>27.871 kDa	430.7578	2	LSTLDLAK	859.5011	859.5014	-0.000305176	91	98
	468.2332	2	SLPDGSFGR	934.4518	934.45087	0.000976563	101	109
	557.2978	3	NARCVLNIYYERR	1668.872	1668.8518	0.01977539	212	224
	664.8837	2	CVLNIYYERR	1327.753	1327.6707	0.08215332	215	224
	742.3789	3	AAEEGYGPLYPGHIPTSPLQK	2224.115	2224.1165	-0.001708984	13	33
	798.8998	2	FVDDEELAYVIQR	1595.785	1595.783	0.002075195	130	142
	835.4404	2	NARCVLNIYYERR	1668.866	1668.8518	0.014404297	212	224
**COQ5**<br>Score 99%<br>Coverage 20.3%<br>32.653 kDa	363.7337	2	VLKPGGR	725.4529	725.4548	-0.00189209	194	200
	553.2802	3	NVTHIDQALQEAYR	1656.819	1656.822	-0.003417969	180	193
	598.8177	2	VYQVFENVAK	1195.621	1195.6238	-0.002929688	26	35
	629.3242	2	SYQYLVESIR	1256.634	1256.6401	-0.006347656	239	248
	842.4482	3	LYDLYSFQVIPVLGEVIAGDWK	2524.323	2524.3252	-0.002441406	217	238
**COQ7**<br>Score 91%<br>Coverage 20.3%<br>20.581 kDa	589.8202	2	IYAGQMAVLGR	1177.626	1177.6277	-0.001831055	32	42
	790.0207	2	YKELLQIIKKFR	1578.027	1577.9656	0.061279297	130	141
**COQ9**<br>Score 15%<br>Coverage 22.6%<br>26.686 kDa	553.2802	3	INDAMNMGHTAKQVK	1656.819	1656.8076	0.010986328	198	212
	559.6355	3	KKTDQFLRDAVEAR	1675.885	1675.9004	-0.015625	97	110
	621.3946	2	LVQLGQAEKKK	1240.775	1240.7502	0.024414062	88	98
	838.95	2	KKTDQFLRDAVEAR	1675.885	1675.9004	-0.015014648	97	110
	1051.008	2	QVKSTGEALVQGLMGAAVTLK	2100.001	2100.1614	-0.16088867	210	230

Supplementary Table 2. Peptide mapping analysis of the purified COQ metabolon. The table lists the peptides obtained from the labelled band excised from the Blue Native Gel shown in Supplementary Figure 8. COQ9 shows a weaker signal suggesting that it could be less well retained by the metabolon during migration.

Supplementary Methods

Construct design, cloning, mutagenesis and expression of the COQs, FDXR and FDX2 - The ancestral sequences were submitted to the Mitofates server for inspection [1]. Ancestral COQ systems - COQ6, COQ3, COQ4 and COQ7 - were all truncated at the end of their mitochondrial targeting sequences. Differently, COQ9 was aligned against the human construct, COQ9_N79 [2], as a reference to determine the cleavage site in the highly disordered N-terminal region. FDXR, FDX2, COQ5, COQ8A and COQ8B were all designed after the release of AlphaFold2 [3]. They were constructed based on a combination of both AlphaFold2 and Mitofates to predict the mitochondrial targeting sequences and additional disordered regions at N-terminus or C-terminus. All sequences have been deposited in the NCBI database (see Data Availability and Supplementary figure 14).

COQ6 was constructed as two forms: COQ6, accounting for the full-length sequence and truncated COQ6 bearing a 35 residue C-terminal truncation. The determination of this truncation was based on limited proteolysis by trypsin digestion experiments that suggested this region was highly flexible. Additionally, the truncation was very similar to that introduced for the successfully crystallized bacterial homologue UbiI [4].

All genes were synthesised, codon optimized for expression in *E. coli* and cloned directly into several different destination vectors (see below) by Genscript. COQ4, truncated COQ6, COQ6 and COQ7 were synthesized with an N-terminal His_6-SUMO tag and cloned into pET-11d pre-destination vectors bearing ampicillin resistance. COQ3, COQ4 and COQ9 were synthesised with N-terminal His_6-SUMO tag and cloned into pET-24a(+) pre-destination vectors possessing Kanamycin resistance. COQ5 was synthesised with an N-terminal Twinned-Strep tag bearing a PreScission protease cleavage site and cloned into a kanamycin-resistant pET-24(a)+ pre-destination vector. COQ3, COQ4, COQ5, COQ7, COQ8A, COQ8B, FDXR and FDX2 were all synthesized as tag-less proteins and inserted into a pGEX-6P-1 destination vector possessing an N-terminal GST-tag and PreScission protease cleavage site together with ampicillin resistance.

COQ4 double mutants were obtained by two subsequent cycles of site-directed mutagenesis PCR using the pGEX-6P-1 construct as template. Primers were purchased from Metabion (Supplementary Table 1). Mutagenesis was performed using the QuickChange methodology, as reported [5]. Briefly a PCR mix (25 µl) was prepared as follows: 12.5 µl PFuUltra II Hotstart PCR Master Mix (Agilent), 1 µl primer forward 10 µM, 1 µl primer reverse 10 µM, 1 µl of template plasmid 100 ng μl^{-1}, 0.4 µl DMSO and 9.1 µl MQ water. Mutations were checked at each cycle by Sanger sequencing (Mycrosynth).

Plasmids containing COQ6 (both full-length and truncated), COQ3, COQ4 (wild-type and double point mutants), COQ7 and COQ9, were transformed by heat shock into *E. coli* BL21 cells (25 s, 42 °C). COQ5, FDXR, FDX2, COQ8A and COQ8B were transformed by heat shock into *E. coli* BL21-CodonPlus-RP cells (25 s, 42 °C). Cells from the resulting colonies were pre-inoculated into 100 ml of LB broth containing 100 µg ml^{-1} of ampicillin and grown overnight at 37 °C. The following morning, 10 mL of the precultures were then transferred to 1 L Terrific Broth and grown at 37 °C, 200 r.p.m. for *ca.* 3 h until the optical density (OD_{600}) reached 0.5-0.7. The protein expression was then induced with isopropyl β-D-1-thiogalactopyranoside, (0.1 mM final), and incubated for 16 h at various temperatures at 200 r.p.m. More specifically, COQ6 full-length, truncated COQ6, COQ3, COQ4 (wild type and double point mutants), COQ7 and COQ9 were expressed at 30 °C; COQ8B, FDXR and FDX2 were expressed at 24 °C; COQ5 and COQ8A were expressed at 16 °C. Cells were harvested by centrifugation (5,000 *g*, 15 min, 10 °C), flash frozen in liquid nitrogen, and stored at −80 °C.

Purification of GST-tagged FDXR, FDX2, COQ3, COQ4, COQ7, COQ8A and COQ8B - Cells (*ca*. 25 g) were resuspended 1:5 in Buffer A (50 mM HEPES pH 7.2, 250 mM NaCl, 10% (v/v) glycerol) supplemented with protease inhibitors (1 mM phenylmethylsulphonyl fluoride, 10 µM leupeptin, 10 µM pepstatin) and 5 µg/g of cells of DNAse I. The cell resuspension was incubated at 4 °C under stirring for 30 min prior to cell lysis performed with a high-pressure homogeniser (Emulsiflex c-3, ATA Scientific) in three cycles.

For soluble proteins (FDXR, FDX2, COQ3) the cell lysate was centrifuged (4 °C, 56,000 *g*, 1 h) using an Avanti J-26 XP centrifuge equipped with a JA-25.15 rotor (Beckman Coulter). The resulting supernatant was filtered through a 0.45 µm filter and loaded onto a gravity column containing Glutathione Sepharose 4B resin (Cytiva) pre-equilibrated with Buffer A. The crude extract was incubated with the resin for 1 h at 4 °C on a rotating plate. Subsequently, the resin was washed with at least five column volumes of Buffer A. The protein was then eluted out with at least ten column volumes of Buffer A supplemented with 50 mM of freshly prepared reduced glutathione. The purified protein sample was concentrated using an Amicon Ultra (Merck) with an appropriate cut-off to 2.5 ml. The reduced glutathione was then removed with a PD-10 desalting column (Cytiva) pre-equilibrated with Buffer A. To cleave the GST-tag the desalted sample was concentrated down to 1 ml and 1 U of GST-tagged PreScission Protease (Cytiva) was added and the sample was incubated overnight at 4 °C on a rotating wheel. The sample was then loaded onto an Äkta Pure system (Cytiva) equipped with a GSTrap-HP 5 ml column (Cytiva). The elution of the tag-less protein

was monitored by following the absorbance at 280 nm for all the proteins. To monitor the flavin cofactor incorporation, the absorbance at 370 nm and 450 nm for FDXR was monitored. The 2Fe-2S cluster absorption at 340 nm and 414 nm for FDX2 was measured.

For membrane proteins (COQ4, COQ7, COQ8A and COQ8B), the cell lysate was exposed to a low-speed centrifugation step (4 °C, 1,200 g, 10 min) to remove cell debris using a C0650 rotor and an Allegra X-30R centrifuge (Beckman Coulter). The supernatant was centrifuged (4 °C, 56,000 *g*, 45 min) to the collect membrane pellet. Pellets were homogenised in Buffer A with a Potter-Elvehjem homogeniser. The total protein concentration was assayed with Biuret reagent based on a calibration line with BSA and adjusted to 10 mg/ml. *n*-Dodecyl β-D-maltoside (DDM, Anatrace) was added to the membrane suspension at 1% (w/v) final concentration and incubated overnight at 4 °C. The detergent solubilised extract was obtained by centrifugation (4 °C, 56,000 *g*, 45 min) collecting the supernatant. The recombinant GST-tagged membrane COQ proteins were then purified as described above for soluble systems, supplementing 0.05% (w/v, final) DDM to all buffers.

The purified proteins were then concentrated down to about 150 µM, assessing the concentration using a NanoDrop ND-100 UV/Vis spectrophotometer (Thermo Scientific) based on the absorption of the prosthetic groups (ε450 nm = 10.9 $mM^{-1}cm^{-1}$ for FDXR, ε414 nm = 11 $mM^{-1}cm^{-1}$ for FDX2) or on the absorption at 280 nm for the other proteins (ε280 nm predicted by ProtParam tool - Expasy). Purity of the samples were evaluated by SDS-PAGE. The purified proteins were then aliquoted, flash frozen in liquid nitrogen and stored at -80 °C.

Purification of HisSUMO tagged COQ6, COQ3, COQ4, COQ7 and COQ9 - Cells (*ca*. 25 g) were resuspended 1:5 in Buffer A (50 mM HEPES pH 7.2, 250 mM NaCl, 10% (v/v) glycerol) supplemented with protease inhibitors (1 mM phenylmethylsulphonyl fluoride, 10 µM leupeptin, 10 µM pepstatin) and 5 µg/g of cells of DNAse I. The cell resuspension was incubated at 4 °C under stirring for 30 min prior to cell lysis performed with a high-pressure homogeniser (Emulsiflex c-3, ATA Scientific) in three cycles.

For soluble proteins (truncated COQ6, COQ3, and COQ9) the cell lysate was centrifuged (4 °C, 56,000 *g*, 1 h) using an Avanti J-26 XP centrifuge equipped with a JA-25.15 rotor (Beckman Coulter). The resulting supernatant was filtered through a 0.45 µm filter and loaded onto a gravity column containing Nickel Sepharose High Performance IMAC resin (Cytiva) pre-equilibrated with Buffer A. The crude extract was incubated with the resin for 1 h at 4 °C on a rotating plate. Subsequently, the

resin was washed with at least five column volumes of Buffer A. The protein was then washed with 5 column volumes of Buffer A supplemented with increasing concentrations of imidazole including 5- and 30-mM wash steps. The protein was then eluted out with at least ten column volumes of Buffer A supplemented with 300 mM imidazole. The purified protein sample was concentrated using an Amicon Ultra (Merck) with an appropriate cut-off to 2.5 ml. The imidazole was then removed with a PD-10 batch desalting column (Cytiva) pre-equilibrated with Buffer A. To cleave the His-SUMO tag the desalted sample was concentrated down to 1 ml and the sample was then mixed with a 6×His-tagged SUMO protease (1.2 mg ml^{-1}) to a volume ratio of 100:1 and incubated overnight at 4 °C on a rotating well. The sample was then loaded onto an Äkta Pure system (Cytiva) equipped with a Ni HiTrap-HP 5 ml column (Cytiva) pre-equilibrated in Buffer A. Proteins were eluted out using an imidazole concentration gradient of 2% buffer B (Buffer A supplemented with 300 mM imidazole) corresponding to 6 mM imidazole. The elution of the tag-less protein was monitored with a multiwavelength detector following the absorbance at 280 nm for all the proteins. For COQ6, the absorption at 370 nm and 450 nm for COQ6 were also measured to account for FAD retention.

For membrane proteins (COQ3, COQ4, COQ6, COQ7) the cell lysate was exposed to a low-speed centrifugation step (4°C, 1,200 *g*, 10 min) to remove cell debris using a C0650 rotor and an Allegra X-30R centrifuge (Beckman Coulter). The supernatant was centrifuged (4 °C, 56,000 *g*, 45 min) to the collect membrane pellet. Pellets were homogenised in Buffer A with a Potter-Elvehjem homogeniser. The total protein concentration was assayed with Biuret reagent based on a calibration line with BSA and adjusted to 10 mg/ml. *n*-Dodecyl β-D-maltoside (DDM, Anantrace) was added to the membrane suspension at 1% (w/v) final concentration and incubated overnight at 4 °C. The detergent solubilised extract was obtained by centrifugation (4 °C, 56,000 *g*, 45 min) collecting the supernatant. The recombinant His-SUMO-tagged membrane COQ proteins were then purified as described above for soluble systems, supplementing 0.05% (w/v, final) DDM to all buffers.

The purified proteins were then concentrated down to about 150 µM, assessing the concentration using a NanoDrop ND-100 UV/Vis spectrophotometer (Thermo Scientific) based on the on the absorption at 280 nm (ε280 nm predicted by ProtParam tool - Expasy). Purity of the samples was evaluated by SDS-PAGE. The purified proteins were then aliquoted, flash frozen in liquid nitrogen and stored at -80 °C.

Purification of Strep tagged COQ5 - Cells (*ca*. 15 g) were resuspended 1:5 in Buffer A (50 mM HEPES pH 7.2, 250 mM NaCl, 10% (v/v) glycerol) supplemented with protease inhibitors (1 mM phenylmethylsulphonyl fluoride, 10 µM leupeptin, 10 µM pepstatin) and 5 µg/g of cells of DNAse I. The cell resuspension was incubated at 4 °C under stirring for 30 min prior to cell lysis performed with a high-pressure homogeniser (Emulsiflex c-3, ATA Scientific) in three cycles.

The cell lysate was exposed to a low-speed centrifugation step (4°C, 1,200 *g*, 10 min) to remove cell debris using a C0650 rotor and an Allegra X-30R centrifuge (Beckman Coulter). The supernatant was centrifuged (4 °C, 56,000 *g*, 45 min) using an Avanti J-26 XP centrifuge equipped with a JA-25.15 rotor (Beckman Coulter) to the collect membrane pellet. Pellets were homogenised in Buffer A with a Potter-Elvehjem homogeniser. The total protein concentration was assayed with Biuret reagent based on a calibration line with BSA and adjusted to 10 mg/ml. FOS-choline 12 (Anatrace) was added to the membrane suspension at 1% (w/v) final concentration and incubated overnight at 4 °C. The detergent solubilised extract was then obtained the following day by centrifugation (4 °C, 56,000 *g*, 45 min) using an Avanti J-26 XP centrifuge equipped with a JA-25.15 rotor (Beckman Coulter) and collecting the supernatant. The crude extract was loaded on 1 ml of Strep-Tactin XT Sepharose (Cytiva) after an incubation of approximately 1 h. Subsequently, the resin was washed with at least five column volumes of Buffer A + 0.05% (w/v, final) DDM. The protein was then eluted with 5 column volumes of Buffer A supplemented with 0.05% (w/v) DDM and 5 mM Biotin. The purified protein sample was concentrated using an Amicon Ultra (Merck) with an appropriate cut-off to 2.5 ml. The Biotin was then removed with a PD-10 batch desalting column (Cytiva) pre-equilibrated with Buffer A + 0.05% (w/v, final) DDM.

To cleave the Twinned-Strep-Prescission tag the desalted sample was concentrated down to 1 ml and the sample was then mixed with 1 U of GST-tagged Prescission Protease (Cytiva) and incubated overnight at 4 °C on a rotating well. The sample was then loaded onto an Äkta Pure system (Cytiva) equipped with a GSTrap-HP 5 ml column (Cytiva) attached *in-tandem* to a Strep-XT-Trap 5 mL column (Cytiva). The tag-less protein eluted in the flow-through using Buffer A + 0.05% (w/v) DDM and monitored with a multi-wavelength detector following the absorbance at 280 nm. The purified protein was then concentrated down to about 150 µM, assessing the concentration using a NanoDrop ND-100 UV/Vis spectrophotometer (Thermo Scientific) based on the on the absorption at 280 nm (ε280 nm predicted by ProtParam tool - Expasy). Purity of the sample was evaluated by SDS-PAGE. The purified protein was then aliquoted, flash frozen in liquid nitrogen and stored at -80 °C.

UV/Visible Spectrometry - UV/Vis spectra of FDXR and FDX2 were recorded using a Diode Array 8453 UV/Vis Spectrophotometer (Agilent) equipped with 10.00 mm quartz cuvettes (Hellma). About 30 µM FDXR was reduced with a five-fold excess of sodium dithionite. Oxidation was attempted by adding oxygenated buffer. After 30 minutes an equimolar concentration of FDX2 was added to attempt FDXR oxidation. Separately, about 30 µM FDX2 was reduced with a five-fold excess of sodium dithionite. Oxidation was elicited by adding oxygenated buffer. UV/Vis spectra were recorded at each step.

4-aminoantipyrine spectrophotometric method of phenols/quinones analysis (HRP assay) - HRP endpoint assay was performed after 10 min reaction of 5 µM truncated COQ6 final volume of 150 µl in Buffer A with 5 µM FDXR, 5 µM FDX2, 1 mM **4a**, 50 µM FAD and 1 mM NADPH at 30 °C 200 rpm in a bench-top incubator. The reaction mixture was supplemented with 2 mM hydrogen peroxide, 0.01 mg ml^{-1} HRP, 0.1 M 4-aminoantipyrine. The quenching reaction was carried out at 30 °C 200 rpm for 30 min. The assay was exploited to catalyse the formation of a quinone-imide adduct between the amide moiety of 4- aminoantipyrine and the 4-hydroxy group of the CoQ_1 intermediates using HRP. The various adducts produced various chromophores: Adducts with 4-hydroxybenzoate (such as **3**) or phenols (such as **4a**) produce chromophores with maxima at λ = 490 nm; Adducts formed with immature hydroquinones (such as **4b**) produce chromophores with λ = 450 nm. Activity was assessed by comparing the shift of the adduct peaks after COQ6 reaction compared to the spectra of the adducts obtained with standard solutions of **4a** and **4b** (Supplementary Fig. 7b). Spectra were recorded using a Diode Array 8453 UV/Vis Spectrophotometer (Agilent) equipped with 10.00 mm quartz cuvettes (Hellma).

Malachite Green ATPase assay - Malachite Green assay was performed after 10 min of reaction of 1 µM COQ8B with 0.1 mM ATP at 30 °C 200 rpm in a bench-top incubator. Activity was tested in the presence of 0.1 mM of each intermediate, or of 1 µM of individual COQ proteins, or a combination of both. Moreover, it was assayed in the presence of the following COQ protein multimers: COQ6-COQ3, COQ7-COQ9 and the COQ metabolon. Basal ATPase activity of COQ8B was assayed by incubating with ATP only. The reactions were quenched by adding 2/3 final of the dye provided by the Malachite Green Phosphate Assay Kit (Sigma-Aldrich). After 10 min of incubation at RT in the dark the absorbance at 620 nm was measured using a ClarioStar plate reader (BMG Labtech) with Greiner 96 transparent flat wells plates. A dilution of the dye in the assay buffer was used as blank.

Absorbance values were converted into µM concentration of phosphate by building a calibration line using 4-40 µM of phosphate analytical standard provided with the kit (Supplementary Fig. 12). Data were analysed by subtracting the basal activity of COQ8B in the absence of any ligand. Linear regression of the standards and data analysis was performed with GraphPad Prism 9. To determine the K_M of ATP for COQ8B, we incubated the enzyme (1 µM) with a range of ATP concentrations (5 – 300 µM) for 10 minutes at 30 °C at 200 rpm (Supplementary Fig. 12). The analysis was performed as described above. GraphPad Prism 9 was used to perform non-linear regression.

Limited proteolysis and peptide mapping - Protein bands were isolated, cut into small fragments and transferred into Eppendorf tubes. The sample was then incubated with (200 µl) of 100 mM ammonium bicarbonate buffer pH 7.8, 50% acetonitrile, to destain the gel fragments. Acetonitrile (100 µl) was then added to dehydrate the gel pieces. The organic solvent was removed by air-drying and 100 µl of 10 mM dithiothreitol solution was added to reduce the sample (30 min at 37 °C). 100 µl of 55 mM iodoacetamide was added in place of dithiothreitol and incubated for 45 min at 60 °C. The solution containing gel pieces was decanted and then immersed twice with 200 µl of 100 mM ammonium bicarbonate with 10 minute incubation periods in between. This solution was decanted, and gel pieces re-dehydrated with acetonitrile (100 µl) until opaque-white in colour. The organic solvent was then removed and the fragments air-dried. Gels were immersed and rehydrated using 100 mM ammonium bicarbonate buffer pH 7.8 (100 µl), with the addition of sequencing grade trypsin (20 ng/µl) (Promega, Madison, WI, USA) for digestion was left overnight at 37 °C. The resultant peptides were extracted using 50% acetonitrile, 5% formic acid solution in water (100 µl). This was performed twice with each step consisting of vortexing for 15 mins. The original supernatant and sequential extractions were collected, dried, and stored at -20 °C. MS analyses were conducted with peptides solubilized in water and 0.1% formic acid (50 µl).

A LC unit (ExionLC TM AD) was employed for all analyses. The unit possessed an accompanying column oven set at 40 °C, in addition to a temperature controlled autosampler (10 °C) and a binary gradient pump system. MS instrument comprises a high resolution QTOF mass spectrometer (AB Sciex X500B) with an accompanying Turbo V Ion source and a Twin Sprayer ESI (electrospray ionization) probe, controlled by SCIEX OS 2.1 software. Peptides were separated using Reverse phase (RP) HPLC using a Hypersil Gold (Thermo Fisher Scientific, USA) C18 column (150 × 2.1 mm, 3 µm particle size, 175 Å pore size) using a linear gradient (2-50% solvent B in 15 min), flow rate of 0.2 ml min^{-1}, with solvents A and B being a 0.1% aqueous FA solution in water and a 0.1% FA solution

made up in acetonitrile, respectively. Positive polarity was employed for mass spectra generation using the following instrument parameters throughout: declustering potential 100 V, curtain gas 30 psi, temperature 350 °C, ion source gas 1 40 psi, ion source gas 2 45 psi, ion spray voltage 4500 V, collision energy 10 V. SCIEX OS 2.1 software was used to acquire spectra, and processed with Peaks studio 4.5 software for protein identification. Resulting masses were then initially searched and screened against the SwissProt and *ad hoc* databases, followed by specific peptide mapping against the ancestral sequences.

Intact protein mass spectrometry – 10 mg ml^{-1} COQ3 was incubated O/N at 30 °C 200 rpm in a bench-top incubator with 1 µM COQ8B and 1 mM ATP in a final volume of 50 µl Buffer A with 0.05% DDM (w/v, final). A negative control reaction was performed by removing ATP from the mixture. Sample was diluted up to 2.5 ml Buffer A; buffer was exchanged to Buffer A with a PD10 batch desalting column (Cytiva) to remove any residual detergent. Sample was then concentrated up to 50 mg ml^{-1} with Amikon Ultra 10K (Merck). Proteins were unfolded by diluting the sample up to 10 mg ml^{-1} in water: acetonitrile 1:1 containing 0.1% FA. A LC unit (ExionLC TM AD) was employed for all analyses. The unit possessed an accompanying column oven set at 40 °C, in addition to an autosampler (10 °C) and a binary gradient pump system. MS instrument comprises a high resolution QTOF mass spectrometer (AB Sciex X500B) with an accompanying Turbo V Ion source and a Twin Sprayer ESI (electrospray ionization) probe, controlled by SCIEX OS 2.1 software. Injection volume was 5 µl (50 µg). Chromatographic separation was carried out with a bioZenTM WidePore C4 column (100 mm length X 2.1 mm diameter, 2.6 µm particle size; Phenomenex). The mobile phase consisted of water (A) and acetonitrile (B) both including 0.1% (v/v) formic acid. Flow rate was set at 0.35 ml min^{-1}. Gradient elution was performed as follows: 15% B at 0.0-0.1 minutes, 15-98% B at 0.1-5.0 minutes, 98% B at 5.0-15.0 minutes, 98-15% B at 15.0-18.0 minutes, 15% B at 18.0-23.0 minutes. Positive polarity was employed for mass spectra generation using the following instrument parameters throughout: declustering potential 100 V, curtain gas 30 psi, temperature 350 °C, ion source gas 1 40 psi, ion source gas 2 45 psi, ion spray voltage 4500 V, collision energy 10 V. Mass deconvolution was performed using BiotoolKit extension of SciexOS 2.1 software: input m/z range 500-5000 Da, mass range 20-70 kDa, isotope resolution 30000.

Micro-Scale Thermophoresis - COQ3 was labelled with a 2nd generation RED-NHS dye (NanoTemper GmbH) following the kit instructions. Briefly, 10 µM protein was incubated at RT in the dark with

300 µM dye. The excess of dye was removed with a B-column pre-equilibrated in buffer A with 0.05% DDM (w/v, final). The labelled protein concentration and the degree of labelling were evaluated based on the absorbance at 280 and 650 nm. Binding assays were carried out in Buffer A supplemented with 0.05% DDM (w/v, final), 0.05% (v/v, final) Tween-20 and 0.1% (w/v, final) Pluronic acid. Final protein concentration was 40 nm. **2** was titrated in serial dilution in the 5 mM - 150 nM range. Binding experiments were performed in the presence and absence of 150 µM $MgCl_2$ and in the presence of 1 mM EDTA as a control.

Nano Differential Scanning Fluorimetry - Thermo-stability analyses were carried out using a TychoTMNT.6 system (NanoTemper GmbH). The proteins were diluted to 1 mg/ml in Buffer A. The protein was pre-incubated with ligands in ice for 15 minutes prior to experiments. Melting curves were obtained following the intrinsic fluorescence of Tryptophan and Tyrosine residues (emission 350 nm and 330 nm, respectively) applying a temperature gradient from 35 °C to 95 °C for a time range of three minutes. Data were analysed as F_{350}/F_{330} ratio and derived to determine the inflection temperature (T_m). All measurements were performed in duplicate. The following condition were tested: COQ3 in the presence of 150 µM $CuSO_4$, $FeSO_4$, $MnSO_4$, $MnCl_2$, $CaSO_4$, $CaCl_2$, $ZnSO_4$, $ZnCl_2$, $MgSO_4$, $MgCl_2$ and 1 mM EDTA; COQ4 in the presence of 25 µM $CuSO_4$, $FeSO_4$, $MnSO_4$, $MnCl_2$, $CaSO_4$, $CaCl_2$, $ZnSO_4$, $ZnCl_2$, $MgSO_4$, $MgCl_2$ and 1 mM EDTA; COQ4 in the presence of $ZnCl_2$ in the 0.095-24.4 µM range. EC_{50} values were determined by plotting the difference between melting temperature in presence and absence of ligand versus the logarithm of the molarity of the ligand and analysing the data by non-linear regression.

References

1. Fukasawa Y, Tsuji J, Fu SC, Tomii K, Horton P, Imai K. MitoFates: improved prediction of mitochondrial targeting sequences and their cleavage sites. *Mol. Cell Proteomics* **14**, 1113-1126 (2015).
2. Lohman D. C. et al. Mitochondrial COQ9 is a lipid-binding protein that associates with COQ7 to enable coenzyme Q biosynthesis. *Proc. Natl. Acad. Sci. U. S. A.* **111**, e4697-705 (2014).
3. Jumper, J., Evans, R., Pritzel, A. et al. Highly accurate protein structure prediction with AlphaFold. *Nature* ***596,*** 583–589 (2021)**.**
4. Hajj C.M. et al. UbiI, a new gene in Escherichia coli coenzyme Q biosynthesis, is involved in aerobic C5-hydroxylation. *J. Biol. Chem*. **288**, 20085-20092 (2013).
5. Kunkel, T. A. Rapid and efficient site-specific mutagenesis without phenotypic selection. *Proc. Natl. Acad. Sci. U. S. A.* **82,** 488– 492 (1985).
